# The Effects of Lead and Cross-Talk Between Lead and Pea Aphids on Defence Responses of Pea Seedlings

**DOI:** 10.3390/ijms252111804

**Published:** 2024-11-02

**Authors:** Iwona Morkunas, Agnieszka Woźniak, Waldemar Bednarski, Adam Ostrowski, Jacek Kęsy, Paulina Glazińska, Julia Wojciechowska, Jan Bocianowski, Renata Rucińska-Sobkowiak, Van Chung Mai, Zbigniew Karolewski, Mateusz Labudda, Anielkis Batista, Philippe Jeandet

**Affiliations:** 1 Department of Plant Physiology, Faculty of Agriculture, Horticulture and Biotechnology, Poznań University of Life Sciences, Wołyńska 35, 60-637 Poznań, Poland; agnieszkam.wozniak@gmail.com (A.W.); julia.wojciechowska95@gmail.com (J.W.); anyelkis@gmail.com (A.B.); 2 Institute of Molecular Physics, Polish Academy of Sciences, Smoluchowskiego 17, 60-179 Poznań, Poland; waldemar.bednarski@ifmpan.poznan.pl (W.B.); adam.ostrowski@ifmpan.poznan.pl (A.O.); 3 Department of Plant Physiology and Biotechnology, Faculty of Biological and Veterinary Sciences, Nicolaus Copernicus University in Toruń, Lwowska 1, 87-100 Toruń, Polandpaulina.glazinska@umk.pl (P.G.); 4 Department of Mathematical and Statistical Methods, Faculty of Agriculture, Horticulture and Biotechnology, Poznań University of Life Sciences, Wojska Polskiego 28, 60-637 Poznań, Poland; jan.bocianowski@up.poznan.pl; 5 Department of Plant Ecophysiology, Faculty of Biology, Adam Mickiewicz University, Uniwersytetu Poznańskiego 6, 61-614 Poznań, Poland; renatar@amu.edu.pl; 6 Department of Research and International Affairs, Vinh University, Le Duan 182, Vinh 43108, Nghe An Province, Vietnam; chung.uni@gmail.com; 7 Department of Phytopathology, Seed Science and Technology, Faculty of Agriculture, Horticulture and Biotechnology, Poznań University of Life Sciences, Collegium Zembala, Dąbrowskiego 159, 60-594 Poznań, Poland; 8 Department of Biochemistry and Microbiology, Institute of Biology, Warsaw University of Life Sciences—SGGW, Nowoursynowska 159, 02-776 Warsaw, Poland; mateusz_labudda@sggw.edu.pl; 9 Polytechnic Institute of Huila, Universidade Mandume ya Ndemufayo, Lubango 3FJP+27X, Angola; 10 Research Unit “Induced Resistance and Plant Bioprotection”, RIBP-USC INRAe 1488, University of Reims Champagne-Ardenne, 51100 Reims, France; philippe.jeandet@univ-reims.fr

**Keywords:** hormesis, lead, *Acyrthosiphon pisum*, *Pisum sativum*, semiquinone radicals, phytohormones, pisatin, soluble sugars, invertases

## Abstract

The main goal of this study was to investigate the effect of lead (Pb) at various concentrations, as an abiotic factor, and the cross-talk between Pb and pea aphid (*Acyrthosiphon pisum* (Harris)) (Hemiptera: Aphididae), as a biotic factor, on the defence responses of pea seedlings (*Pisum sativum* L. cv. Cysterski). The analysis of growth parameters for pea seedlings demonstrated that Pb at a low concentration, i.e., 0.025–0.0625 mM Pb(NO_3_)_2_, caused a hormesis effect, i.e., stimulation of seedling growth, whereas Pb at higher concentrations, i.e., 0.01–0.325 mM Pb(NO_3_)_2_, inhibited growth, which manifested as the inhibition of length and fresh biomass. The differences in the level of the main defence-related phytohormones, such as abscisic acid (ABA), jasmonic acid (JA) and salicylic acid (SA), and indole-3-acetic acid (IAA)—an auxin stimulating plant cell growth—depended on the dose of Pb, aphid infestation and direct contact of the stress factor with the organ. A high accumulation of soluble sugars in the organs of pea seedlings both at sublethal doses and hormetic doses at early experimental time points was observed. At 0 h and 24 h of the experiment, the hormetic doses of Pb significantly stimulated invertase activities, especially in the roots. Moreover, an increase was observed in the pisatin concentration in pea seedlings growing in the presence of different concentrations of Pb and in the case of cross-talk between Pb and *A. pisum* in relation to the control. Additionally, a significant induction of the expressions of *isoflavone synthase (IFS)* and *6α-hydroxymaackiain 3-O-methyltransferase (HMM)* genes, which participate in the regulation of the pisatin biosynthesis pathway, in pea seedlings growing under the influence of sublethal 0.5 mM Pb(NO_3_)_2_ and hormetic 0.075 mM Pb(NO_3_)_2_ doses of Pb was noted. The obtained results showed that the response of *P. sativum* seedlings depends on the Pb dose applied, direct contact of the stress factor with the organ and the duration of contact.

## 1. Introduction

The dose–response relationship is the basis of toxicology and one of the most discussed scientific issues over the 20th century [1]. Moreover, as reported by Calabrese [2], the hormetic dose–response model is the most common and fundamental model in the biological and biomedical sciences. Currently, hormesis as a dose–response phenomenon is not only incredibly important in toxicology, but it is an intensively researched area of biology because exposure to a toxic substance in a hormetic dose upregulates adaptive mechanisms of the organism, protecting against subsequent exposures to stress factors. Heavy metals, including lead (Pb) as an abiotic stress factor used in these studies, belong to the group of the most dangerous pollutants in ecotoxicology. Lead-contaminated soil originating from anthropogenic sources is widespread [3] and poses serious hazards to environment and human health. Moreover, Yu et al. [4] presented that road dust containing Pb can act as a temporary sink of metals from a variety of sources and as a source of metals contributing to atmospheric pollution through resuspension. Pb phytotoxicity has been well documented in many plant species [5]. Pb can be absorbed by plants and mainly accumulates in root tissues [6]. Our earlier review work reported that the presence of heavy metals at a hormetic dose in the surrounding environment affects the response of plants to biotic stressors [7]. The hormesis phenomenon, also called ‘priming’, results in a faster and stronger induction of basal defence mechanisms upon subsequent biotic stress factors. The induction of this adaptive response of plants to moderate environmental stressors involves several steps, such as the perception and transduction of the stress-signal, stimulation of the hormetic response at the transcriptional and post-transcriptional levels, and increase in the metabolic status of plant cells [8]. Our latest research also shows the changes in the expression of genes encoding enzymes of phytohormone biosynthesis under the influence of Pb at hormetic doses and sublethal doses [9]. From the literature data, we know that the hormetic dose response can occur as a direct stimulatory response, after an initial disruption in homeostasis followed by a modest overcompensation response, or as a response to an ‘adapting’ or ‘pre-conditioning’ dose that is followed by a stronger challenging dose [2,10].

The perception of aphid infestation induces highly coordinated and sequential defensive reactions in plants at the cellular and molecular levels [11]. For example, elevated levels of semiquinone radicals may contribute to the build-up of a defensive barrier against aphids, i.e., these radicals may be incorporated into polymers such as lignins by combining with reactive free radicals (ROS) and could prevent the breakdown of associated cell walls [12]. The sealing of plant cell walls is extremely important because aphids insert stylets of their sucking–piercing mouthparts into leaf tissues and penetrate cells to follow the apoplastic pathway until they reach phloem sap [13]. As reported by Tjallingii and Esch [14], aphid stylets pierce through the epidermis, mesophyll, as well as other parenchymatous tissues until they reach the phloem. The factors responsible for the activation of plant defences include mechanical damage and elicitors, which are present in aphid saliva [15]. Furthermore, in defence response signal cascades, apart from hydrogen peroxide (H_2_O_2_) and nitric oxide (NO), defence-related phytohormones also take part, such as JA, SA, ethylene (ET), ABA and gibberellic acid (GA). It should also be mentioned that pisatin, a 6a-hydroxyl-pterocarpan phytoalexin in peas, is an essential element of the plant defence system. Pisatin is relatively unique among naturally occurring pterocarpans by virtue of the (+) stereochemistry of its 6a–11a C–C bond [16]. As reported by Cruickshank and Perrin [17], this pterocarpan was the first chemically identified phytoalexin. Furthermore, Dixon [18] showed that it is a product of the isoflavonoid pathway. As reported by Jeandet [19], plants use phytoalexins as defensive substances in response to pests and pathogens. In turn, soluble sugars, such as sucrose and its monosaccharides, can not only be the donors of the carbon skeletons for secondary metabolism but also signalling molecules regulating the expression of genes encoding isoflavonoid biosynthesis enzymes [11] and references cited in this paper. Bolouri Moghaddam and Van den Ende [20] pay attention to the dual role of sucrose as a metabolisable transport sugar and a potential ‘priming’ molecule in plant defence reactions.

Foyer et al. [21] noted that the plants exposed to one stressor are more susceptible to attack by herbivores and pathogens. However, in many cases, even mild exposures to abiotic stresses trigger innate immune responses and enhance plant defences. Therefore, in the case of impact, one stressor followed by another stressor triggers cross-tolerance phenomena [21]. Furthermore, Rossatto et al. [22] suggest the occurrence of a phenomenon associated with memory in plants, where the pre-exposure of plants to potentially stressful conditions can stimulate tolerance to subsequent stresses.

The main goal was to determine the effect of Pb in a concentration range of 0.025–0.325 mM Pb(NO_3_)_2_ and cross-talk between Pb and *A. pisum* on growth parameters of pea seedlings (*Pisum sativum* L. cv. Cysterski) as well as molecular and metabolic responses under the influence of these stress factors.

Therefore, apart from growth analysis, one of the goals was to determine the effect of Pb in the above range and cross-talk between Pb and *A. pisum* on early defensive responses, i.e., concentrations of semiquinone radicals and paramagnetic manganese (Mn^2+^) ions and phytohormones. The novelty of these studies is the correlation between the applied doses of Pb, cross-talk between Pb and *A. pisum* infestation and saccharide levels and invertase activities. Moreover, the effects of Pb and *A. pisum* on the expression of genes encoding enzymes that are involved in the regulation of the pisatin biosynthetic pathway, i.e., *IFS* and *HMM*, in pea seedlings were determined. HMM is an enzyme that catalyses the reaction of the direct transformation of 6α-hydroxymaackiain into pisatin. Additionally, in the present work, the normality of distribution of the investigated traits was demonstrated, and relationships between the values of individual traits in organs using correlation analysis were examined.

We can hypothesise in our studies that Pb at low concentrations can trigger hormetic responses, enhancing plant growth and the activities of invertases, which are enzymes that metabolise sucrose into its monosaccharides. In turn, soluble sugars are carbon skeleton donors for secondary metabolism via which pisatin biosynthesis occurs. Moreover, a hormetic dose can cause molecular and metabolic changes in relation to semiquinone radicals and paramagnetic Mn^2+^ ions, phytohormones, soluble sugars and pisatin levels. Additionally, changes in the soluble sugar level in plant cells will affect gene expression encoding *IFS* and *HMM*, which play a key role in the biosynthesis of pisatin.

Our research is important because the pea aphid (*A. pisum*) as a biotic stress factor is widely used in laboratory studies and is one of the key pests of pulse crops worldwide [23]. The broad host (pea, clover, alfalfa and broad bean) range of the pea aphid [24,25,26], complex life cycle, including both sexual and parthenogenetic reproduction [27], and flexibility in adapting to different environmental conditions [28] make it difficult to control this pest. The pea aphid is closely related to important crop pests, including the green peach aphid (*Myzus persicae*) and the Russian wheat aphid (*Diuraphis noxia*). *A. pisum* is a serious concern for commercial pulse producers because it can injure the crops directly by removing sap from leaves, stems and pods and indirectly by acting as a vector for over 30 plant viruses, including cucumber mosaic virus, beet yellows virus, pea enation mosaic virus and bean leafroll virus [29,30,31]. The International Aphid Genomics Consortium [32] analysis of the pea aphid genome has begun to reveal the genetic underpinnings of this animal’s complex ecology, including its capacity to parasitise agricultural crops, its association with microbial symbionts and its developmental patterning.

Air and soil pollution, including heavy metals, can affect plant–insect relationships directly or indirectly, can be toxic or cause the hormesis effect and can change their behaviour and metabolism [7]. Therefore, this study is valuable, especially in the era of global climate change (GCC), where an increase in the temperature accelerates the development of crop pests.

## 2. Results

### 2.1. Effects of Lead and A. pisum on the Growth of Pea Seedlings

The results of measurements of the length and fresh weight of the epicotyls of pea seedlings exposed to low concentrations of Pb showed that this heavy metal causes the hormetic effect (Table 1; Figure S1). Therefore, hormetic concentrations of Pb in the range of 0.025–0.0625 mM Pb(NO_3_)_2_ stimulate the growth of the epicotyls, i.e., their length and fresh weight, which is clearly visible at 24 and 48 h of the experiment. However, these changes in the above variants are only significant in comparison to the control at 24 h of the experiment. Also, stimulation of the growth of 24 h epicotyls as a result of the exposure to cross-talk between 0.05 and 0.065 mM Pb(NO_3_)_2_ and pea aphid feeding was found. In turn, sublethal concentrations in the range of 0.1–0.325 mM Pb(NO_3_)_2_ led to the inhibition of the growth of the epicotyls and roots of pea seedlings. Inhibition of the growth of the epicotyls and roots of pea seedlings at the mentioned concentrations was visible both at the beginning of the experiment, i.e., after 4 days from the application of Pb(NO_3_)_2_, before the aphids were transferred to the pea seedlings (at 0 h) and at subsequent time points, i.e., at 24, 48 and 72 h of the experiment. Moreover, a slight increase in the length of the epicotyls and the roots of pea seedlings growing on the media with 0.025–0.0625 mM Pb(NO_3_) with the time of the experiment was observed. The highest values of the length of the epicotyls and roots for the 0.0625 mM Pb(NO_3_) and 0.05 mM Pb(NO_3_) variants at 72 h of the experiment, respectively, were found. However, these changes were not significant in comparison to the control. Furthermore, at 24, 48 and 72 h, the fresh weight of the roots from a hormetic dose (0.0625 mM Pb(NO_3_) variant) was higher than in the control; these changes were significant in comparison to the control.

Hypotheses on the equality of means were verified using the two-sample *t*-test. To account for multiple testing, we used the Bonferroni correction. Comparisons between particular levels of the analysed factor in the roots (Table S1) and leaves (Table S2) of pea seedlings at different times (independently) used the two-sample *t*-test for equal means for all observed traits. Mean squares from the two-way analysis of variance of the observed traits in the roots and leaves are shown in Tables S3 and S4, respectively. In turn, the correlation coefficients and *p*-values for the values of individual traits in the roots and the leaves are shown in Table S5.

Significant inhibition of the growth of both the epicotyls as well as the roots of pea seedlings growing on a medium with a sublethal concentration, such as 0.25–0.325 mM Pb(NO_3_)_2_, was found. The lowest values of the growth of the epicotyls and roots resulted from the effects of the highest sublethal concentration of Pb, i.e., 0.325 mM Pb(NO_3_)_2_ and cross-talk between 0.325 mM Pb(NO_3_)_2_ and pea aphid infestation. These changes were significant in comparison to the control. Moreover, under the combined effect of Pb and the pea aphid, a slight growth of the epicotyls and the roots of the pea seedlings compared with time was found (Table 1; Figure S1). It was also shown that the roots are much more sensitive to the effects of Pb than the epicotyls because the roots of the pea seedlings at 0, 24, 48 and 72 h treated with a sublethal dose, such as 0.325 mM Pb(NO_3_)_2_, were 32–55% shorter and had 59–72% lower fresh weight than the control, while the epicotyls of the pea seedlings growing on a medium with 0.325 mM Pb(NO_3_)_2_ were 4–18% shorter and had 22–39% lower fresh weight than the epicotyls from the control.

### 2.2. Effects of Lead and A. pisum on Semiquinone Radical and Manganese Ion Concentrations in Pea Seedlings

In electron paramagnetic resonance (EPR), spectra lines from Mn^2+^ ions (sextet) and semiquinone radicals (single, slightly asymmetric line) can be distinguished. For the leaves of the pea seedlings, the radical EPR line is characterised by the g-value 2.0029 (±0.0003) and the line width 7.0 (±0.2) Gs. In turn, in the roots of the pea seedlings, free radicals give a signal with the g-value 2.0050 (±0.0005) and the linewidth 8.0 (±0.4) Gs.

**Figure 1:**
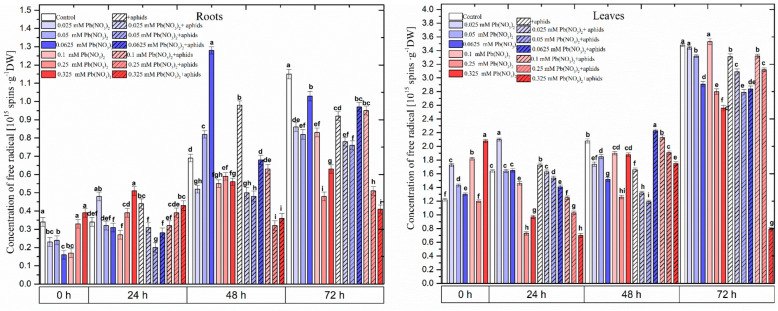

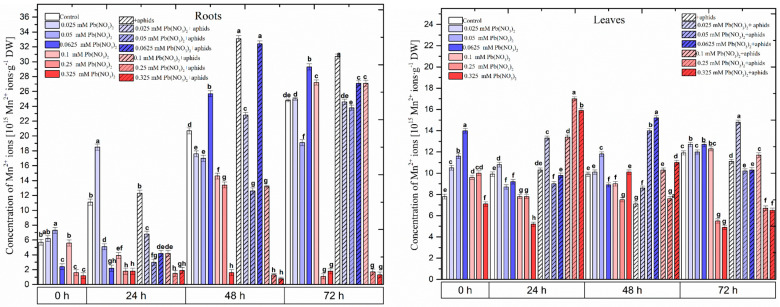
The concentrations of semiquinone radicals and the Mn^2+^ ions in the roots and leaves of pea seedlings growing on the Hoagland medium with varied concentrations of Pb, i.e., 0.025 Pb(NO_3_)_2_, 0.05 Pb(NO_3_)_2_, 0.0625 Pb(NO_3_)_2_, 0.1 Pb(NO_3_)_2_, 0.25 Pb(NO_3_)_2_ and 0.325 Pb(NO_3_)_2_, in the organs of pea seedlings growing on the Hoagland medium with varied concentrations of Pb and colonised by pea aphids, *A. pisum*, and in the organs of pea seedlings growing on the Hoagland medium colonised by pea aphids, *A. pisum*. The data were obtained in three independent experiments and statistically analysed using ANOVA (*p*-values at α = 0.05). In the figures, for individual times, the means denoted by the same letters are not significantly different.

The results from EPR spectroscopy show that in the case of Pb-treated variants, a remarkable increase in the levels of semiquinone radicals in the roots of the pea seedlings growing on the media with 0.025, 0.050 and 0.0625 mM Pb(NO_3_)_2_ in a period from 0 h to 48 h of the experiment was noted (Figure 1; Table S6). The highest level of semiquinone radicals, i.e., 1.283 × 10^15^ spin·g^−1^ DW, was observed at 48 h in the roots of pea seedlings treated by 0.0625 mM Pb(NO_3_)_2_, which was 1.85- and 3.63-fold higher than in the control at the same time point and at the beginning (0 h), respectively. Moreover, it should be emphasised that sublethal concentrations of Pb (i.e., 0.1, 0.25 and 0.325 mM Pb(NO_3_)_2_) induced the generation of semiquinone radicals to a lesser extent than hormetic concentrations of Pb (Figure 1; Table S6). For the Pb- and aphid-treated variants, we observed a strong generation of semiquinone radicals, which remained at high levels in the roots of the pea seedlings from 48 h to 72 h post-infestation (hpi). It should also be mentioned that at 72 h of the experiment, the concentration of these radicals in the roots of the pea seedlings from all experimental variants was lower than in the control.

In turn, the semiquinone radical contents in the leaves were higher than in the roots (Figure 1; Table S6). At 0 h of the experiment, i.e., four days after Pb administration and prior to transferring the aphids onto the pea seedlings, a strong generation of semiquinone radicals in the leaves was observed both for the variant that received a hormetic Pb concentration (0.025 Pb(NO_3_)_2_) and for the sublethal Pb concentration variants (0.1 and 0.325 mM Pb(NO_3_)_2_). At 24 h, the high concentration of these radicals was also seen in the variant with a hormetic dose (0.025 mM Pb(NO_3_)_2_), which was higher than in the control.

However, at 72 h, the highest level of semiquinone radicals in pea seedling leaves treated with 0.10 mM Pb(NO_3_)_2_ was 3.523 × 10^15^ spin·g^−1^ DW. At the same time point, a high level of these radicals in pea seedling leaves for the 0.10 mM Pb(NO_3_)_2_ + aphid variant, i.e., 3.330 × 10^15^ spin·g^–1^ DW, was noted. However, the levels of semiquinone radicals in all investigated variants were lower than in the control (Figure 1; Table S6).

As early as at 24 h, a strong accumulation of Mn^2+^ ions in the roots of the pea seedlings growing with a hormetic dose of 0.025 mM Pb(NO_3_)_2_ was observed; the concentration of Mn^2+^ ions in the above roots was 58% higher than in the control (Figure 1; Table S6). Moreover, at 48 h, a very strong increase in the level of the Mn^2+^ ions, especially in the case of the hormetic dose of 0.0625 mM Pb(NO_3_)_2_ and during cross-talk between 0.0625 mM Pb(NO_3_)_2_ and aphid infestation, was recorded. The levels of Mn^2^ ions were 24% and 57% higher in these roots than in the control, respectively. Also, aphid infestation alone caused a very strong accumulation of Mn^2+^ ions in the roots. At the next time point, i.e., at 72 h, the high concentration of Mn^2+^ ions in the above variants was maintained. An interesting result is the very low level of Mn^2+^ ions in the roots of the pea seedlings growing on the medium with sublethal doses, i.e., 0.25 and 0.325 mM Pb(NO_3_)_2_ and during cross-talk between 0.25 Pb(NO_3_)_2_ + aphids and 0.325 mM Pb(NO_3_)_2_ +aphids. Furthermore, the level of Mn^2+^ ions was higher in the roots than in the leaves (Figure 1; Table S6). At 0 h of the experiment, in the leaves of the pea seedlings from the 0.025 to 0.0625 mM Pb(NO_3_)_2_ variants, an increase in Mn^2+^ ions was observed in comparison to the control (Figure 1; Table S6). In turn, at 24 hpi, an accumulation of Mn^2+^ ions in the leaves from the variants 0.025 mM Pb(NO_3_)_2_ +aphids, 0.1 mM Pb(NO_3_)_2_ +aphids, 0.25 mM Pb(NO_3_)_2_ +aphids and 0.325 mM Pb(NO_3_)_2_ +aphids was noted; the levels of these ions were higher by 38%, 75%, 112% and 100%, respectively. At 24 hpi, in the leaves of the pea seedlings cultured on the medium with a hormetic dose of 0.025 mM Pb(NO_3_)_2_ and infested by aphids as well as with sublethal doses, such as 0.1, 0.25 and 0.325 mM Pb(NO_3_), and infested by aphids, higher concentrations of Mn^2+^ ions in relation to the control were observed. Moreover, in the 48 leaves of the pea seedlings from the 0.05 mM Pb(NO_3_), 0.05 mM Pb(NO_3_)_2_ +aphids and 0.0625 mM Pb(NO_3_)_2_ +aphids variants, higher concentrations of Mn^2+^ ions were recorded than in the control seedlings. At 24 and 72 h of the experiment, a low concentration of Mn^2+^ ions in the leaves for the variant with a sublethal dose (0.325 mM Pb(NO_3_)) was demonstrated.

### 2.3. Effects of Lead and A. pisum on Concentrations of Phytohormones

**Figure 2:**
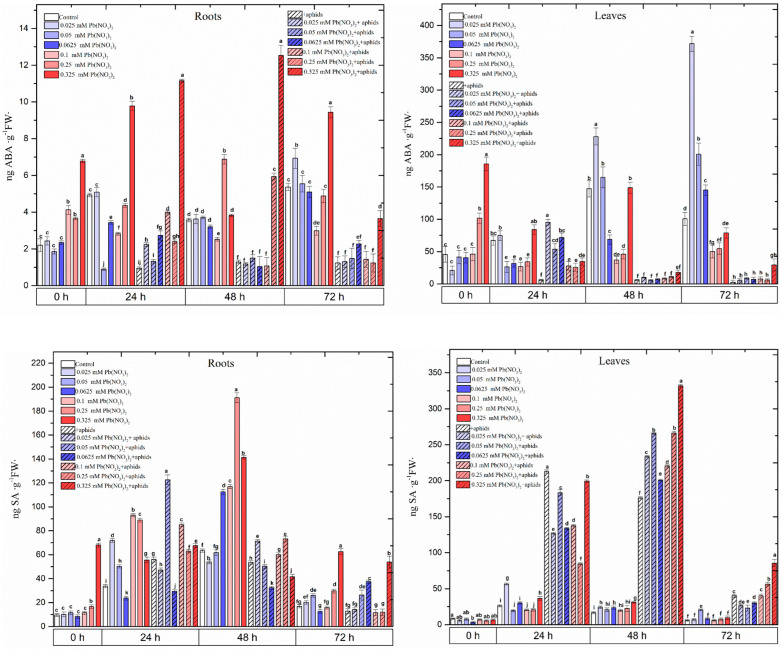

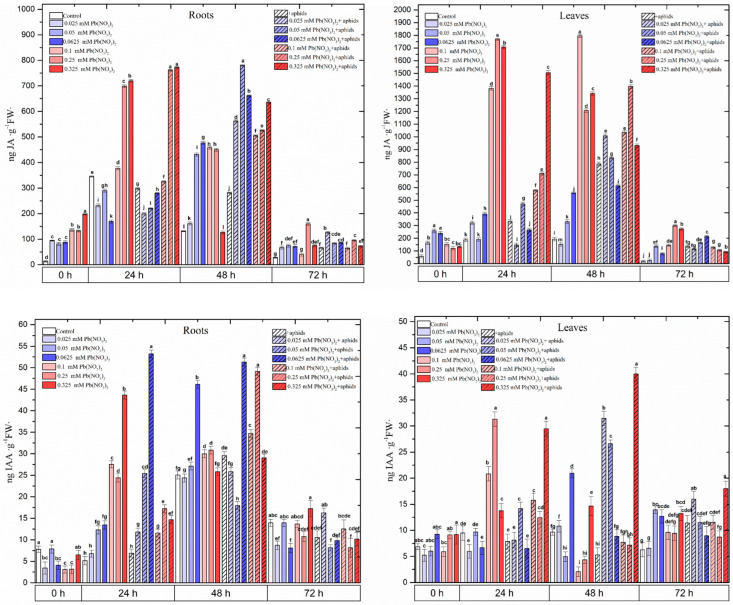
The concentrations of phytohormones in the organs of pea seedlings growing on the Hoagland medium with varied concentrations of Pb, i.e., 0.025 Pb(NO_3_)_2_, 0.05 Pb(NO_3_)_2_, 0.0625 Pb(NO_3_)_2_, 0.1 Pb(NO_3_)_2_, 0.25 Pb(NO_3_)_2_ and 0.325 Pb(NO_3_)_2_, in the organs of pea seedlings growing on the Hoagland medium with varied concentrations of Pb and colonised by pea aphids, *A. pisum*, and in the organs of pea seedlings growing on the Hoagland medium colonised by pea aphids, *A. pisum*. The data were obtained in three independent experiments and statistically analysed using ANOVA (*p*-values at α = 0.05). In the figures, for individual times, the means denoted by the same letters are not significantly different.

The analytical results of the QuEChERS method showed a significant accumulation of ABA in the roots and leaves of the pea seedlings growing on the medium with a hormetic dose of 0.025 Pb(NO_3_)_2_ and a sublethal dose of 0.325 mM Pb(NO_3_)_2_ (Figure 2; Table S7). The contents of ABA in these variants were significantly higher than in the control and others. It should be emphasised that the ABA levels were significantly higher in the leaves than in the roots of the pea seedlings. The highest level of ABA in the 72 h leaves of the pea seedlings growing on the medium with a hormetic dose (0.025 mM Pb(NO_3_)_2_) was noted; this level was 371.72 ng·g^−1^ FW. Also, in the 72 h leaves from the 0.05 and 0.0625 mM Pb(NO_3_)_2_ variants, the concentration of ABA was significantly higher than in the control. In addition to this, a high level of ABA was also recorded at 48 h in the leaves from the hormetic dose (0.025 mM Pb(NO_3_)_2_). Moreover, in both the roots and the leaves of the pea seedlings growing on the media with hormetic doses as well as with sublethal doses and infested by aphids, the concentrations of pisatin were generally lower than in the control (Figure 2; Table S7).

At the beginning of the experiment (at 0 h), in the roots growing on the medium with a sublethal dose, such as 0.325 mM Pb(NO_3_), four times higher SA accumulation in comparison to the control was detected (Figure 2; Table S7). At all time points, a significantly higher concentration of SA was observed in the roots of the pea seedlings growing on the media with sublethal doses than in the control roots. The highest level of SA in the 48 h roots of the pea seedlings from 0 to the 1 mM Pb(NO_3_)_2_ variant was 190 ng SA·g^−1^ FW. Pea aphid infestation also enhanced the accumulation of SA in the 24 h pea seedling roots, both in the +aphids variant and in variants such as 0.025 mM Pb(NO_3_)_2_ +aphids, 0.05 mM Pb(NO_3_)_2_ +aphids, 0.1 mM Pb(NO_3_)_2_ +aphids, 0.25 mM Pb(NO_3_)_2_ +aphids and 0.325 mM Pb(NO_3_)_2_ +aphids in relation to the control. In turn, the contents of this phytohormone in the leaves of the investigated variants were not significant in comparison to the control (exception for the 0.025 mM Pb(NO_3_)_2_ variant) (Figure 2; Table S7). It should be emphasised that at 24 and 48 h, very high concentrations of SA in the pea seedling leaves infested by aphids or growing on the medium with Pb(NO_3_)_2_, both at hormetic doses as well as sublethal doses and infested by aphids, were observed. The highest level of SA in the 48 h leaves from the 0.325 mM Pb(NO_3_)_2_ variant was noted; this level was 325 ng SA·g^−1^ FW.

Even at 0 h, a significant accumulation of JA was recorded in the pea seedlings growing on Hoagland medium with all tested concentrations of Pb(NO_3_)_2_ (Figure 2; Table S7). The content of JA in the roots and leaves of the pea seedlings growing on the medium with Pb(NO_3_)_2_ was much higher than in the control. Additionally, pea aphid infestation induced the accumulation of JA in the variants cultured on the medium with Pb(NO_3_)_2_ at both the hormetic and sublethal doses; these levels were significantly higher than in the control. Therefore, the highest contents of JA were noted in the roots from the 0.05 mM Pb(NO_3_)_2_ +aphids and 0.325 mM Pb(NO_3_)_2_ +aphids variants. Furthermore, at 24 h and 48 h, high levels of JA were determined in both the roots and the leaves of the pea seedlings cultured on the media with sublethal doses of Pb. Hormetic doses also stimulated the accumulation of JA in the above organs of the pea seedlings. The levels of JA in the leaves were significantly higher than in the roots of the pea seedlings.

From 0 h to 48 h of the experiment, the concentration of IAA in the pea seedling roots growing on the media with the hormetic doses (0.05 and 0.0625 mM Pb(NO_3_)) and the sublethal doses (0.1–0.325 mM Pb(NO_3_)) increased (Figure 2; Table S7). High IAA concentrations, especially in the roots from the 0.325 mM Pb(NO_3_) and 0.0625 mM Pb(NO_3_) variants, were detected. Additionally, pea aphid infestation in the 0.0625 mM Pb(NO_3_) variant enhanced the accumulation of IAA in the 24 h and 48 h pea seedling roots.

In the 24 h leaves of the pea seedlings, the accumulation of IAA was detected for sublethal doses of 0.1–0.25 mM Pb(NO_3_) (Figure 2; Table S7). Pea aphid feeding on the leaves of the pea seedlings growing on the medium with 0.325 mM Pb(NO_3_) significantly enhanced this level. In addition to this, a high concentration of IAA in the leaves from hormetic doses, such as the 0.025 and 0.05 mM Pb(NO_3_) variants, was revealed.

### 2.4. Effects of Lead and A. pisum on Pisatin Content in Pea Seedlings

**Figure 3:**
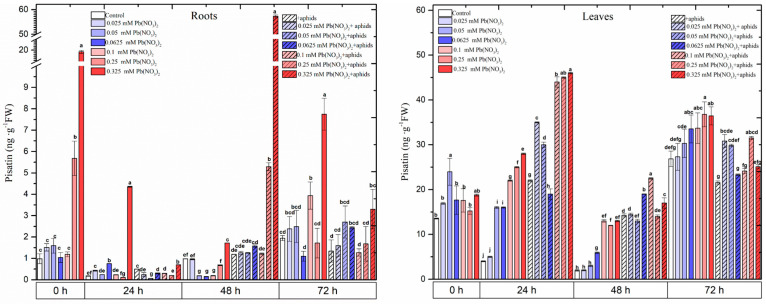
The concentrations of pisatin in the organs of pea seedlings growing on the Hoagland medium with varied concentrations of Pb, i.e., 0.025 Pb(NO_3_)_2_, 0.05 Pb(NO_3_)_2_, 0.0625 Pb(NO_3_)_2_, 0.1 Pb(NO_3_)_2_, 0.25 Pb(NO_3_)_2_ and 0.325 Pb(NO_3_)_2_, in the organs of pea seedlings growing on the Hoagland medium with varied concentrations of Pb and colonised by pea aphids, *A. pisum*, and in the organs of pea seedlings growing on the Hoagland medium colonised by pea aphids, *A. pisum*. The data were obtained in three independent experiments and statistically analysed using ANOVA (*p*-values at α = 0.05). In the figures, for individual times, the means denoted by the same letters are not significantly different.

Even at 0 h, the content of pisatin was strongly induced by sublethal doses of Pb (i.e., 0.25 mM and 0.325 mM Pb(NO_3_)_2_) and then remarkably reduced at 24 h and 48 h (Figure 3; Table S8). The level of pisatin at the above sublethal doses of Pb was over 5 and 20 times higher than in the control. Additionally, in 48 h roots infested by the pea aphid, especially in the variant with 0.325 mM Pb(NO_3_)_2_, a significant increase in the level of pisatin was observed. The concentration of this phytoalexin was 58 ng·g^−1^ DW. Moreover, at 24 and 72 h, an increase in the levels of pisatin in the roots of the pea seedlings growing on media with hormetic doses (0.0625 mM Pb(NO_3_)_2_, 0.025 mM Pb(NO_3_)_2_ and 0.05 mM Pb(NO_3_)_2_) was observed. In addition to this, the levels of pisatin in the 24 h pea seedling roots infested by the pea aphid (+aphids variants), the 48 h roots growing on the medium with a hormetic dose of 0.0625 mM Pb(NO_3_)_2_ and the 72 h roots growing on the media with 0.05 mM Pb(NO_3_)_2_ and 0.0625 mM Pb(NO_3_)_2_ were higher than in the control. In turn, the levels of pisatin were significantly higher in the leaves of the pea seedlings than in the roots (Tables S7 and S8). In the leaves of the pea seedlings growing on the media with the hormetic doses, increases in the pisatin content were observed. From 0 h to 72 h of the experiment, the concentrations of pisatin in the leaves with hormetic doses, especially 0.05 mM Pb(NO_3_)_2_ and 0.0625 mM Pb(NO_3_)_2_, were generally higher than in the control. Also, from 24 to 48 hpi in the leaves infested by the pea aphid (+aphids variants), increases in the pisatin content were noted. Additionally, the 24 and 48 h leaves of the seedlings growing on the media with hormetic doses (0.025 mM Pb(NO_3_)_2_ and 0.05 mM Pb(NO_3_)_2_) and infested by the pea aphid showed significantly higher levels of pisatin than the control. Moreover, sublethal doses of Pb significantly stimulated pisatin accumulation (Figure 3; Table S8). The highest contents of pisatin were recorded in the leaves of the seedlings growing on the media with 0.1 mM, 0.25 mM and 0.325 mM Pb(NO_3_)_2_ and infested by the pea aphid.

### 2.5. Effects of Lead and A. pisum on Soluble Sugar Content in Pea Seedlings

**Figure 4:**
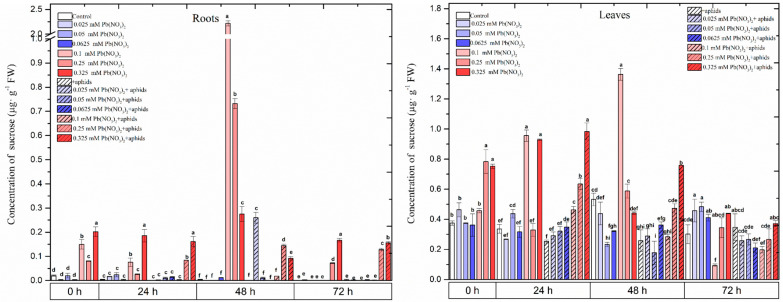

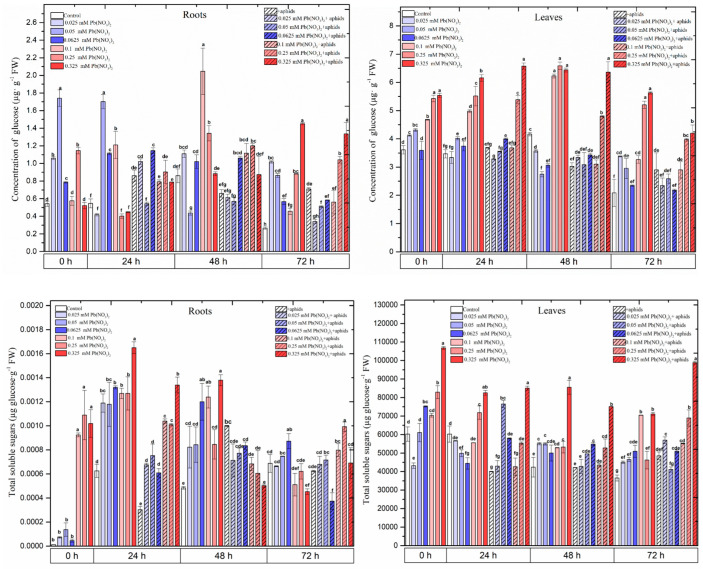
The concentrations of sucrose (a, b), glucose (c, d) and total soluble sugars (e, f) in the organs of pea seedlings growing on the Hoagland medium with varied concentrations of Pb, i.e., 0.025 Pb(NO_3_)_2_, 0.05 Pb(NO_3_)_2_, 0.0625 Pb(NO_3_)_2_, 0.1 Pb(NO_3_)_2_, 0.25 Pb(NO_3_)_2_ and 0.325 Pb(NO_3_)_2_, in the organs of pea seedlings growing on the Hoagland medium with varied concentrations of Pb and colonised by pea aphids, *A. pisum*, and in the organs of pea seedlings growing on the Hoagland medium colonised by pea aphids, *A. pisum*. The data were obtained in three independent experiments and statistically analysed using ANOVA (*p*-values at α = 0.05). In the figures, for individual times, the means denoted by the same letters are not significantly different.

From 0 to 72 h, in the roots and leaves of the pea seedlings cultured on the media with sublethal doses of Pb (0.1, 0.25 and 0.325 mM Pb(NO_3_)_2_), high concentrations of sucrose were detected (Figure 4; Table S9). The highest sucrose content was noted in the 48 h roots of the pea seedlings of the 0.1 mM Pb(NO_3_)_2_ variant. Also, at 48 h of the experiment, a high concentration of sucrose was observed in the roots of the pea seedlings of the 0.25 mM Pb(NO_3_)_2_ variant, which was seven times higher than in the control. The high contents of sucrose were induced by the high concentrations of Pb (i.e., 0.1–0.325 mM Pb(NO_3_)_2_), and the highest amount of sucrose was 1.3 µg × g^−1^ DW in the 48 h leaves of the pea seedlings growing on the medium with 0.1 mM Pb(NO_3_)_2_ (Figure 4; Table S9). From 0 h to 48 h, the level of sucrose in the leaves of the pea seedlings with sublethal doses of Pb was higher than in the control. Moreover, it was shown that the cross-talk between Pb and *A. pisum* infestation stimulated the accumulation of sucrose in the roots of the 0.25 Pb(NO_3_)_2_ +aphids and 0.325 mM Pb(NO_3_)_2_ +aphids variants, and these contents were higher than the control roots. A similar effect was observed in the 24 and 48 h leaves of the pea seedlings growing on the medium with sublethal doses of Pb(NO_3_)_2_. It should also be added that in the variant with a hormetic dose of 0.05 mM Pb(NO_3_)_2_ and *A. pisum* infestation, the accumulation of sucrose in the roots was noted.

In turn, in the case of glucose, high levels of this sugar were detected in the pea seedling roots for variants with hormetic doses (Figure 4; Table S9). From 0 h to 72 h of the experiment, in these roots, the level of glucose was generally higher than in the control roots. In turn, the accumulation of glucose was found in the leaves of the pea seedlings growing with sublethal doses of Pb. The level of glucose in the 0.1, 0.25 and 0.325 mM Pb(NO_3_)_2_ variants was higher than in the control leaves. Additionally, at all times after aphid infestation in the leaves of the pea seedlings infested by the aphids and cultured on the medium with the above concentrations of Pb, high contents of glucose were detected. The highest concentration of glucose in the leaves from the 0.325 mM Pb(NO_3_)_2_ +aphid variant was 6.5 µg × g^−1^ DW.

From 0 h to 72 h, high accumulations of total soluble sugars in the roots from both hormetic and sublethal doses of Pb occurred. The highest levels of total soluble sugars were detected in the 24 h roots of the pea seedlings for the 0.325 mM Pb(NO_3_)_2_ and 0.325 mM Pb(NO_3_)_2_ + aphids variants; these levels were over 2.5 and 2.0 times higher than the control, respectively. In addition to this, at 0 h, the highest total soluble sugar content was noted in the pea seedling leaves from the 0.325 mM Pb(NO_3_)_2_ variant; it was over 1.5 times higher than in the control leaves. Regarding the hormetic dose of 0.0625 mM Pb(NO_3_)_2_, the total soluble sugar content in the pea seedling leaves was 20% higher than in the control. These high levels of total soluble sugars in the above variant and the 0.0625 mM Pb(NO_3_)_2_ + aphids variant were maintained at all time points.

### 2.6. Effects of Lead and A. pisum on Acid Invertase and Alkaline Invertase Activities

**Figure 5:**
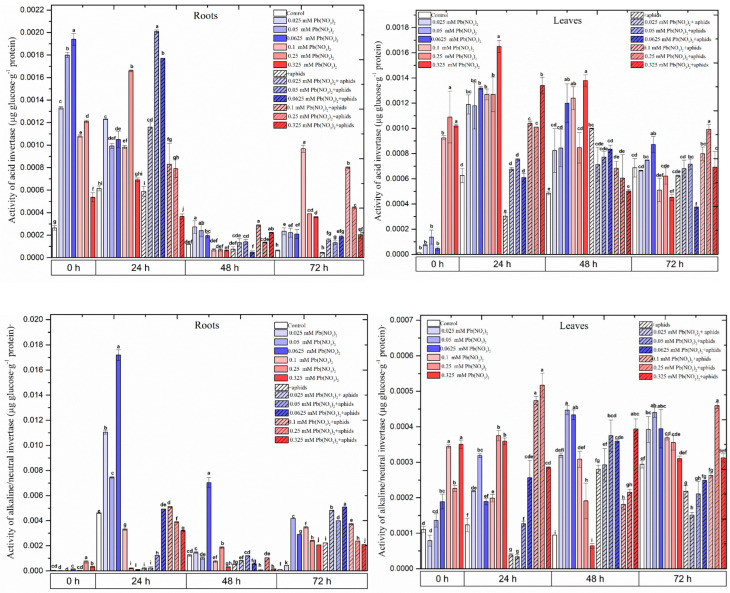
Effects of lead and *A. pisum* on acid invertase and alkaline invertase activities in the roots and leaves of pea seedlings. The data were obtained in three independent experiments and statistically analysed using ANOVA (α = 0.05). In the figures, for individual times, the means denoted by the same letters are not significantly different.

At the beginning of the experiment (at 0 h), a very high increase in the activity of acid invertase in the roots growing on the media with the hormetic doses and the sublethal doses in relation to the control was observed (Figure 5; Table S10). Strong stimulation of the acid invertase activity with hormetic doses—the 0.05 and 0.0625 mM Pb(NO_3_)_2_ variants—was noted. Also, high activity of the above enzyme in the roots from the sublethal doses of Pb was observed. For example, in the roots from the 0.1 and 0.25 mM Pb(NO_3_)_2_ variants, the activity of acid invertase was more than four times higher than in the control. Additionally, pea aphid infestation very strongly raised the activity of acid invertase in the hormetic dose variants. In turn, from 0 h to 48 h, a very high level of acid invertase was observed in the leaves of the pea seedlings growing on the media with the sublethal doses of Pb (Figure 5; Table S10). Pea aphid infestation in the variants with hormetic and sublethal doses of Pb caused increases in the activity of acid invertase. In turn, in the case of alkaline/neutral invertase, strong stimulation of the activity of this enzyme occurred, especially in the roots from the hormetic doses. The highest activity of the above enzyme was detected in the roots growing with the sublethal dose of 0.0625 mM Pb(NO_3_)_2_; it was four times higher than in the control. Also, at 48 h, high activity of this enzyme was observed in the mentioned variant; it was seven times higher than in the control. However, it should be mentioned that after 24 h of the experiment, a strong decrease in the activity of alkaline/neutral invertase was observed in comparison to 48 and 72 h of the experiment. At 72 h, the activity of this enzyme was higher in the roots of the stressed variants than in the control.

### 2.7. Correlation Coefficients Between Observed Traits in Relation to Roots and Leaves of Pea Seedlings in the Context of the Effects of Lead and A. pisum

**Figure 6:**
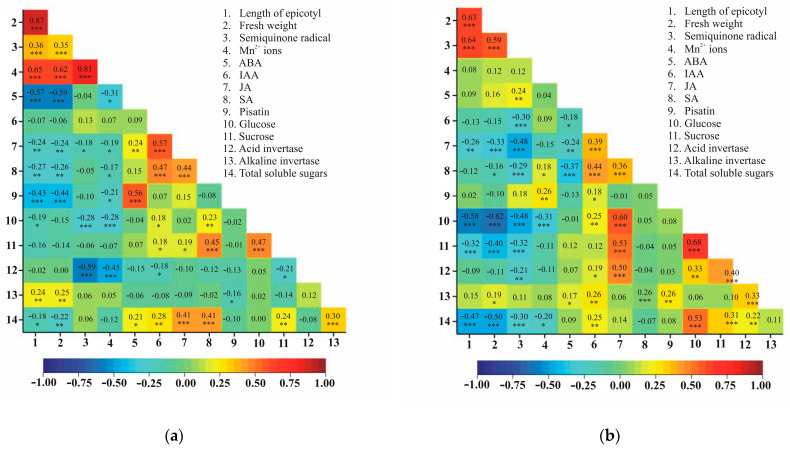
Heatmaps for Pearson’s correlation coefficients between the observed traits (*r*_0.001_ = 0.23) in the roots (**a**) and the leaves (**b**). Each cell denotes the correlation coefficient between a pair of observed traits. The correlation coefficients ranged from –1 (blue) to 1 (red). The correlation coefficients and *p*-values for the values of individual traits in roots and leaves, * *p* < 0.05; ** *p* < 0.01; *** *p* < 0.001.

Positive significant statistical correlations in both the roots and the leaves were observed between the length of the epicotyl and fresh weight, length of the epicotyl and semiquinone radical, fresh weight and semiquinone radical, IAA and JA, IAA and SA, JA and SA, IAA and glucose, IAA and sucrose, glucose and sucrose, fresh weight and alkaline invertase, IAA and total soluble sugars as well as sucrose and total soluble sugars. A negative correlation in both the roots and the leaves was observed between the length of the epicotyl and JA, fresh weight and JA, semiquinone radical and JA, fresh weight and SA, length of the epicotyl and glucose, semiquinone radical and glucose, semiquinone radical and acid invertase, length of the epicotyl and total soluble sugars as well as fresh weight and total soluble sugars. For the six pairs of traits, the correlation coefficients had different signs in the roots and the leaves. The correlation between ABA and JA was positive in the roots and negative in the leaves. An inverse correlation—negative in the roots and positive in the leaves—was observed for the following trait pairs: Mn^2+^ ions and SA, Mn^2+^ ions and pisatin, IAA and acid invertase, sucrose and acid invertase as well as pisatin and alkaline invertase (Figure 6).

### 2.8. Effects of Lead and A. pisum on Expression Levels of Isoflavone Synthase (IFS) and 6α-Hydroxymaackiain 3-O-Methyltransferase (HMM) in Roots and Leaves of Pea Seedlings

**Figure 7:**
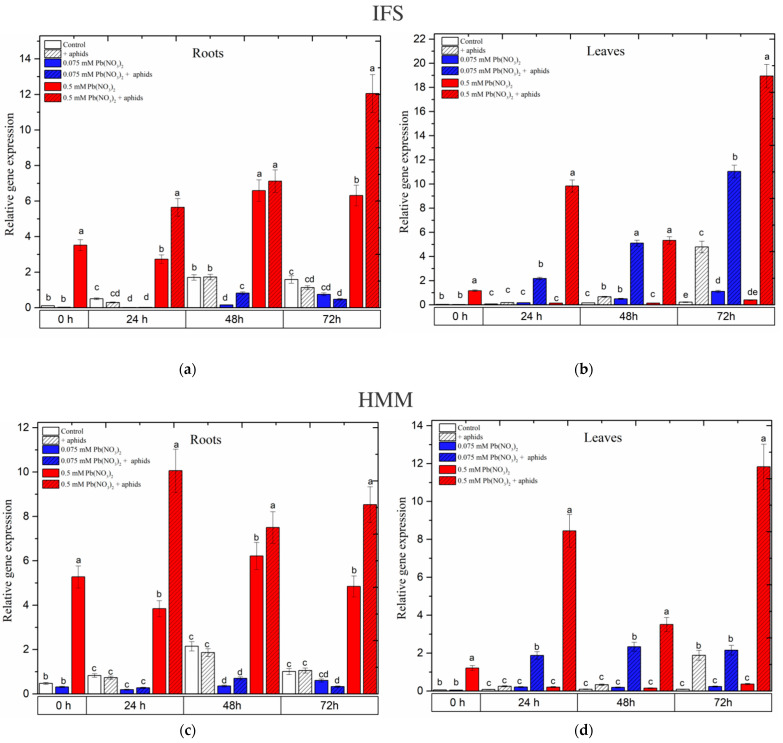
Expression of *IFS* (**a**,**b**) and *HMM* (**c**,**d**) in the roots and the leaves of pea seedlings (*P. sativum*): (**a**,**c**) roots; (**b**,**d**) leaves. Bars present standard errors. The data were obtained in three independent experiments and statistically analysed using ANOVA (α = 0.05). In the figures, for individual times, the means denoted by the same letters are not significantly different.

In the roots of the pea seedlings, the sublethal dose of Pb (0.5 mM Pb(NO_3_)_2_) induced *IFS* and *HMM* expression (Figure 7a,c). A high level of *IFS* and *HMM* transcripts in the roots from the 0.5 mM Pb(NO_3_)_2_ variant was maintained at all time points, i.e., from the start of the experiments to 72 h. Additionally, *A. pisum* infestation on the pea seedlings caused a generally strong upregulation of the above-mentioned genes in the roots growing at a sublethal Pb dose at some experimental time points. In turn, in the roots of the pea seedlings from a hormetic dose, such as 0.075 mM Pb(NO_3_)_2_, a low level of expression of the above genes was detected; the relative expressions of *IFS* and *HMM* were lower than that in the control (Figure 7a,c). In the leaves of the pea seedlings from both the sublethal and hormetic doses and infested by aphids (0.5 mM Pb(NO_3_)_2_ and 0.075 mM Pb(NO_3_)_2_ variants), very high expressions of the *IFS* and *HMM* genes were recorded at all time points. However, these levels of the transcripts for *IFS* and *HMM* in the leaves from the 0.075 mM Pb(NO_3_)_2_ variant were lower than in the leaves from the 0.05 mM Pb(NO_3_)_2_ variant. Also, pea aphid feeding alone enhanced the expression of these genes in the leaves of the pea seedlings (Figure 7b,d).

## 3. Discussion

The greatest novelty of this work is showing, for the first time, the involvement of sucrose, glucose and invertases in the defence response at the metabolic level in the edible pea (*P. sativum* L. Cysterski) on the exposure of the plant to varying concentrations of Pb, i.e., at low Pb doses, such as 0.025–0.0625 mM Pb(NO_3_)_2_, causing the hormesis effect, and at high Pb doses, such as 0.01–0.325 mM Pb(NO_3_)_2_, causing a sublethal effect, as well as during cross-talk between Pb and the pea aphid (*A. pisum*) (Harris) infestation. Additionally, we show here, for the first time, changes in the expressions of the *IFS* and *HMM* genes in pea seedlings growing under a hormetic or sublethal dose of Pb and during cross-talk between Pb and *A. pisum* infestation.

Simultaneously, we also demonstrate early defence responses, such as the generation of semiquinone radicals and phytohormones in the organs of pea seedlings, at the above Pb(NO_3_)_2_ concentrations. These studies are valuable because they are further evidence demonstrating the phenomenon of hormesis under experimental conditions. First, our results show a significant stimulation of pea seedling growth and raised metabolic status at hormetic doses, i.e., 0.025–0.0625 mM Pb(NO_3_)_2_. This effect is best visible at 24 and 48 h of the experiment (Table 1; Figure S1). Therefore, our research results are evidence of the phenomenon of hormesis, i.e., the stimulation of the length of the epicotyls as well as the fresh weight at hormetic doses of Pb and also during the combinatory effect of the two stressors Pb and *A. pisum*.

Salinitro et al. [8] demonstrated that urban concentrations of Pb, Cd and Cr do not inhibit plant growth in species such as hairy bittercress (*Cardamine hirsuta* L.), annual meadow grass (*Poa annua* L.) and chickweed (*Stellaria media* L.) but cause hormesis, leading to a considerable increase in plant growth. In the case of the highest Pb concentration, i.e., at 15 μM Pb-EDTA, a consistent increase in the biomass in *P. annua* and *S. media* was observed. Also, Tang [33] showed the highest plant biomass increase for both the shoots and roots in comparison to the control at a hormetic dose of 48 µM Pb(NO_3_)_2_.

Our research has shown that apart from the stimulation of growth at a hormetic dose, we also observed an increase in molecular and metabolic parameters. The results of our studies showed that at 0 and 24 h, the glucose contents in the roots were the highest for a hormetic dose, such as the 0.05 mM Pb(NO_3_)_2_ variant, among the tested variants, which may support epicotyl growth at the next time point, i.e., 48 h of the experiment. In parallel, the high glucose levels in hormetic variants at 0 h of the experiment may be related to high acid invertase activity. Sugars may not only perform a nutritional function but may also activate signalling systems that lead to altering a variety of plant defence responses that include resource allocation and growth. In parallel, apart from growth stimulation of epicotyls at 48 h of the experiment, a very distinct enhanced metabolic status of plant cells, i.e., an increase in the concentration of semiquinone radicals in the roots of the pea seedlings at a hormetic dose of 0.0625 mM Pb(NO_3_)_2_ (Figure 1; Table S6), was recorded. The above result indicates that an increase in the generation of free radicals may play a significant defence role, especially during the early plant–aphid interaction. It was also shown in this work that the level of semiquinone radicals in the leaves was higher than in the roots, which is certainly related to the higher metabolism of leaves than roots because both the processes of photosynthesis and respiration take place in the leaves. We provide further evidence raising the importance of semiquinone radicals in the context of the plant defence response to stress factors. As reported by Song and Buettner [34], semiquinones are free radicals resulting from the removal of one hydrogen atom with its electron during the process of dehydrogenation of a hydroquinone to a quinone or, alternatively, the addition of a single hydrogen atom with its electron to a quinone. The reduction of a quinone can occur in two sequential one-electron-transfer reactions to generate hydroquinone via semiquinone [35,36]. Molecules containing a quinone/semiquinone/hydroquinone moiety can serve as either one- or two-electron acceptors/donors. The intermediate semiquinone is a relatively stable free radical if we compare to highly reactive free radicals such as the hydroxyl radical. In turn, semiquinone radicals are relatively unstable species in comparison to quinones and hydroquinones. As reported by War et al. [37], quinones formed by the oxidation of phenols during the induction of the defence mechanism of plants against herbivorous insects bind covalently to leaf proteins and inhibit protein digestion in herbivores. Additionally, quinones may also exhibit direct toxicity to insects. As reported by Leszczyński et al. [38], such a wound-induced metabolism induces changes in both free oxygen radicals, cyanide and quinones as well as quantitative and qualitative changes in the endogenous metabolites of plant cells.

Regardless of the foregoing, the obtained results in the presented work revealed the highest levels of paramagnetic Mn^2+^ ions in the roots both at a hormetic dose of 0.0625 mM Pb(NO_3_)_2_ and during the cross-talk between 0.0625 mM Pb(NO_3_)_2_ and *A. pisum* (Figure 1; Table S6). Additionally, pea aphid infestation alone in the roots (+aphids variant) enhanced the Mn^2+^ ion content. Manganese is one of the essential micronutrients for the growth and development of plants. It should be emphasised that Mn plays crucial roles in the physiological and biochemical processes of plants, whose intensity is important for plant defence reactions. The high levels of Mn^2+^ ions in the roots from the hormetic variants and the combined effects of Pb at a hormetic dose and feeding by the pea aphid may indicate strong activation of the defence mechanisms aimed at overcoming stress. It is worth emphasising here that the very high level of Mn^2+^ ions could also be due to the sucking of phloem sap by aphids, which would suggest a systematic transmission of the signal from the leaves to the roots. It is known that the plant defence response to aphid feeding is the result of mechanical damage due to wounding by narrow piercing–sucking mouthparts called stylets and the subsequent secretion of elicitors that are present in the saliva [14,39,40,41,42,43].

The metabolism of plants constantly changes due to fluctuations in environmental factors [5]. It is well known that under stress conditions, one of the first biochemical responses is the generation of free radicals and defence-related phytohormones [12]. Our studies also revealed a high accumulation of defence-related phytohormones such as ABA, JA and SA as a result of exposure to sublethal doses, such as 0.01–0.325 mM Pb(NO_3_)_2_, and during cross-talk between Pb and the pea aphid infestation (Figure 2; Table S7). The high levels of these phytohormones may indicate their involvement in the defence strategy of pea seedlings against the above stress factors. These phytohormones play a crucial role in the regulation of various stress responses at the physiological, biochemical and molecular levels. It has also been revealed that a high accumulation of the above phytohormones was noted at the hormetic doses of Pb (0.025–0.0625 mM Pb(NO_3_)_2_), during cross-talk between these Pb doses and pea aphid infestation and *A. pisum* infestation alone (Figure 2, Table S7). The high level of defence-related phytohormones also suggests an induction of the signal cascade upregulating various stress responses in the organs of pea seedlings, e.g., pisatin biosynthesis. In the 24 h roots of the pea seedlings, both an increase in the level of SA in the hormetic variants as well as a higher level of pisatin in relation to the control were observed. Moreover, the result concerning the very high level of ABA in the leaves of the pea seedlings from the hormetic dose of 0.025 mM Pb(NO_3_)_2_ is noteworthy.

Dias et al. [44] raised an important role for JA and SA in the organs of four-week old *P. sativum* plants exposed to 10, 100 and 500 mg kg^−1^ Pb in the soil. It was revealed that JA was induced in *P. sativum* organs, even at the lowest Pb doses, while SA and ABA increased, particularly in the leaves, at high concentrations of Pb in the soil.

On the other hand, in our work, the highest levels of IAA, especially at the hormetic dose of 0.0625 mM Pb(NO_3_)_2_ and during cross-talk between Pb and pea aphid infestation (Figure 2; Table S7), were observed. As reported by Campanella et al. [45], IAA stimulates gene expression, cell division, cell elongation and differentiation in plant tissue. Our research results demonstrate the interdependence between a very high concentration of JA in both the roots and the leaves of pea seedlings from sublethal doses of Pb (0.25 and 0.35 mM Pb(NO_3_)_2_) and a high concentration of pisatin in the above variants and also during the combinatory effect of the two stressors Pb and *A. pisum*.

However, the novelty of this work is to show the level of soluble sugars in the organs of pea seedlings at the above hormetic and sublethal doses (Figure 4; Table S9). An interesting result in these studies was the low sucrose level, especially in the roots of the pea seedlings at hormetic doses. This result may indicate that sucrose is hydrolysed to monosaccharides. The low sucrose levels at the hormetic doses of Pb are associated with high invertase activities and a high level of glucose, especially in the roots. Additionally, aphid infestation enhanced acid invertase activities at the hormetic dose 0.05 mM Pb(NO_3_)_2_ in the roots of pea seedlings (Figure 5, Table S10). Moreover, our data show a significant increase in the content of sucrose in the leaves at sublethal doses of Pb and during pea aphid feeding, as well as increased invertase activity.

As reported by Van Rensburg and Van den Ende [46], fluctuations in sugar levels are clearly associated with metabolic responses. Sugars constitute the primary substrate providing energy and structural material for defence responses in plants, while they may also act as signal molecules interacting with the hormonal signalling network that regulates the plant immune system [47,48]. Numerous studies revealed that sugars can be directed to various biochemical pathways supporting plant defence reactions and play a key role as signalling molecules during plant development and stress conditions [20,49,50,51,52,53,54,55,56]. Glucose, sucrose and trehalose-6-phosphate (T6P) are the most studied sugars for their roles in metabolic signalling in plants [46]. UDP-glucose has been proposed as a potential intracellular mediator of ROS signalling and PCD. The above authors showed that soluble sugars are important participants in the plant stress tolerance mechanism. Also, Van den Ende and Peshew [57] proposed that soluble vacuolar sugars may take part in vacuolar antioxidant processes, which are intimately linked to the well-known cytosolic antioxidant processes induced by stress factors. As reported by Khan et al. [58], plants reallocate their resources, including energy use, to the adaptive response mechanism to stress tolerance while maintaining a balanced reduction–oxidation status. Fluctuations in the level of soluble sugars in the tested variants may indicate that these saccharides may be directed to the secondary metabolism via which pisatin is synthesised. It was also revealed in our work that high sublethal doses of Pb caused an increase in the concentration of pisatin (Figure 3; Table S8). Additionally, pea aphid feeding at sublethal doses enhanced this concentration. The highest level of pisatin (58 ng·g^−1^ FW) in the leaves cultured on the medium with 0.325 mM Pb(NO_3_)_2_ and infested by aphids may be the result of signal transmission from the leaves to the roots. The concentration of pisatin in most of the investigated variants was significantly higher than in the control plants, and the hormetic doses of Pb induced the higher content of pisatin in pea seedling organs. Preisig et al. [59] demonstrated that pterocarpan (+)pisatin is the main phytoalexin synthesised by *Pisum* sativum. Phytoalexins are antimicrobial substances synthesised in the plant defence response to biotic stress factors [60]. It is important to highlight that a hormetic dose of Pb(NO_3_)_2_ and *A. pisum* infestation upregulated the expressions of the *IFS* and *HMM* genes in the leaves of the pea seedlings (Figure 7, Table S11). Also, we noted the induction of the expressions of the *IFS* and *HMM* genes under conditions of a sublethal dose of Pb and during cross-talk between a sublethal dose and pea aphid infestation.

It is also worth emphasising the high activity of invertases, both acid and alkaline/neutral invertases, in the roots of the pea seedlings growing on the media with hormetic doses of Pb. Also, *A. pisum* infestation at hormetic doses (0.025–0.0625 mM Pb(NO_3_)_2_) stimulated the activities of invertases, especially at earlier time points (Figure 5; Table S10). Invertases are sucrose-hydrolysing enzymes that are often associated with plant tissues acting as physiological sinks. Invertases are essential enzymes for coordinating sugar metabolism, plant responses to stress and sugar signalling [61]. Furthermore, in our studies, strong inhibition of the growth of epicotyls of the pea seedlings exposed to sublethal doses (0.01–0.325 mM Pb(NO_3_)_2_) and pea aphid feeding results from the fact that the aphid, by taking plant sap containing assimilates, causes only small amounts of these compounds to reach the epicotyl growth zone [62].

## 4. Materials and Methods

### 4.1. Plant Material and Growth Conditions

Pea (*Pisum sativum* L. cv. Cysterski) seeds of the S-elite class (2017–2023, [https://phr.pl/en/products/sowing-pea/edible-sowing-pea-cysterski/]) were used in the experiments. They were obtained from the Plant Breeding Company in Tulce near Poznań in Poland.

The genotype of the edible sowing pea Cysterski is characterised by a very low height and is an early variety. It has white and fine seeds that are excellent for feeding to pigeons. In addition, the Cysterski variety of edible pea is resistant to diseases caused by fungal pathogens, such as pea fusarium wilt, ascochyta blight, powdery mildew and downy mildew. Moreover, this genotype has an early flowering and ripening date. Additionally, it has white flowers. It is an evenly ripening cultivar, recommended for cultivation both nationwide in Poland as well as very popular abroad. The plants have very good rigidity and resistance to lodging [https://phr.pl/en/products/sowing-pea/edible-sowing-pea-cysterski/, (accessed on 23 October 2024)].

In our experiment, surface sterilisation of the seeds was performed as described by Mai et al. [12,63] and Morkunas et al. [45]. After 6 h of imbibition, the seeds were transferred onto filter paper (in Petri dishes) and immersed in a small amount of water to support further absorption. After a subsequent 66 h, the seed coats were removed from the germinating seeds. Next, the germinating seeds (*n* = 35) were transferred to hydroponic grow boxes containing Hoagland medium (the control). The hydroponic boxes were covered with dark foil to mimic soil conditions. On the fifth day, the medium was replaced in all the hydroponic variants, and Pb was added to the medium at 0.025 mM Pb(NO_3_)_2_, 0.05 mM Pb(NO_3_)_2_, 0.0625 mM Pb(NO_3_)_2_, 0.1 mM Pb(NO_3_)_2_, 0.25 mM Pb(NO_3_)_2_ and 0.325 mM Pb(NO_3_)_2_. After the next four days, pea seedlings were infested by pea aphids. Regardless of the above, for the analysis of the expression of genes encoding the enzymes of pisatin biosynthesis, such as *IFS* and *HMM*, pea seedlings were grown with a hormetic dose (0.075 mM (PbNO_3_)_2_) or a sublethal dose (0.5 (Pb(NO_3_)_2_) of Pb. The experiment involved only adult insects. Samples for analyses were collected four days after Pb administration, prior to transferring the aphids onto pea seedlings (at 0 h) and then after 24, 48 and 72 h of both stress treatments. During the experiment, hydroponic cultures were aerated with an aeration system. Pea seedlings, both the control and Pb-treated samples, as well as the seedlings grown in the presence of Pb and infested by *A. pisum*, were cultured in glass aquariums (30 cm × 22 cm × 28 cm) and protected with gauze. The experiment was conducted in a phytotron at 22–23 °C, 65% relative humidity and light intensity of 130–150 μmol photons m^−2^s^−1^ with a 14/10 h (light/dark) photoperiod. The experimental variants were as follows: control pea seedlings cultured without Pb and not colonised by pea aphids (*A. pisum*), pea seedlings growing on the Hoagland medium with varied concentrations of Pb, i.e., 0.025 mM Pb(NO_3_)_2_, 0.05 mM Pb(NO_3_)_2_, 0.0625 mM Pb(NO_3_)_2_, 0.1 mM Pb(NO_3_)_2_, 0.25 mM Pb(NO_3_)_2_ and 0.325 mM Pb(NO_3_)_2_, pea seedlings growing on the Hoagland medium with varied concentrations of Pb and colonised by pea aphids, *A. pisum*, and pea seedlings growing on the Hoagland medium colonised by pea aphids, *A. pisum*. Moreover, in the case of the analysis of the expression of genes, such as *IFS* and *HMM*, the following experimental variants were applied: control pea seedlings cultured without Pb and not colonised by *A. pisum*, pea seedlings growing on the Hoagland medium with varied concentrations of Pb, i.e., 0.075 mM Pb(NO_3_)_2_ and 0.5 mM Pb(NO_3_)_2_, pea seedlings growing on the Hoagland medium with varied concentrations of Pb and colonised by *A. pisum* and pea seedlings growing on the Hoagland medium colonised by *A. pisum*. Furthermore, it should be stressed that the epicotyl and roots were used for measurements of the length and fresh weight. In turn, the roots and leaves of pea seedlings were used as objects for all other studies carried out in this work.

### 4.2. Aphids and Infestation Experiment

*A. pisum* (Harris), originally cultured at the Department of Plant Physiology, the Poznań University of Life Sciences, Poland, was reared on *Pisum sativum* L. cv. Cysterski in a growth chamber under conditions as specified above. On day 11 of culture, pea seedlings were infested with 20 apterous adult females of *A. pisum* using a fine paintbrush. The aphid populations were monitored throughout all the experiments [9]. The control pea seedlings were cultured with no addition of Pb and not colonised by pea aphids.

It is worth mentioning that *A. pisum* is colloquially known as the green dolphin [64]. The pea aphid is a model organism for biological studies whose genome has been sequenced and annotated [32]. The genotype of the pea aphid (*A. pisum*) can reproduce either sexually or asexually (parthenogenetically), giving rise, in each case, to almost identical adults [65]. Additionally, as reported by Duncan et al. [66], pea aphids, during the spring and summer, reproduce asexually and viviparously, giving birth to live young, or nymphs. In the case of asexually produced nymphs, oogenesis and embryonic development is initiated in late development, leading to fast, telescoped generations, which has an impact on the rapid growth of aphid populations [67] and their success as a pest species [32]. Additionally, pea aphids belong to biotypes that are either susceptible or resistant to the fungal entomopathogen *Erynia neoaphidis* [68,69], and this was influenced by the temperature [70].

### 4.3. Morphometric Measurements of the Epicotyl and Roots of Pea Seedlings

Pea seedlings for morphometric measurements, i.e., the length and fresh weight, were collected at 0 h, 24 h, 48 h and 72 h of culture. The results were then averaged based on the measurements of at least 20 organs and were expressed as per 1 organ, i.e., in cm organ^−1^ and fresh weight in g organ^−1^.

### 4.4. Determination of Semiquinone Radical and Manganese Ion Concentrations

Radicals and paramagnetic Mn^2+^ ions were detected in the leaves and roots of seedling peas using the electron paramagnetic resonance (EPR) technique. Fresh samples of pea organs (at least 1000 mg) were frozen in liquid nitrogen, and a Jouan LP3 freeze dryer was applied for lyophilisation. The lyophilised material was placed in quartz tubes (diameter: 4 mm) and then in an EPR spectrometer. Electron paramagnetic resonance spectra were recorded at room temperature using a Bruker ELEXSYS X-band spectrometer (Rheinstetten, Germany). The A of Al_2_O_3_:Cr^3+^ with a known number of paramagnetic centres was used as the concentration standard [12,62]. The concentrations of radicals and paramagnetic Mn^2+^ ions in the pea organs were determined after comparing double-integrated EPR lines from paramagnetic complexes in the sample and the standard. The concentrations of Mn^2+^ ions and semiquinone radicals were calculated as the number of paramagnetic complexes per 1 g of dry weight sample.

### 4.5. Detection of Phytohormones and Pisatin Concentrations

The salicylic acid (SA), abscisic acid (ABA), jasmonic acid (JA/MeJA), indole-3-acetic acid (IAA) and pisatin were determined using the modified QuEChERS method of Perestrelo et al. [71] using internal deuterated standards and followed by liquid chromatography tandem mass spectrometry (LC–MS/MS). In the case of pisatin, d_6_-ABA as IS was used. Approximately 100 mg of plant tissue was ground in liquid nitrogen and further homogenised with 1.6 mL of 80% (*v*/*v*) acetonitrile containing 1 mM 2,6-di-tert-butyl-4-methylphenol and 5% (*w*/*v*) formic acid.

The homogenates were agitated overnight at 4 °C; then, about 100 mg total of MgSO_4_ and NaCl (3:1) was added. Samples were subsequently vortexed and agitated for 30 min at room temperature. The next homogenates were centrifuged at 20,000× *g* for 10 min at 4 °C to separate the water and acetonitrile phases, and the upper acetonitrile phase was transferred to new tubes. Afterwards, the organic phase was evaporated to dryness under a nitrogen stream at 45 °C, and the remaining residue was suspended in 1 mL of 1 M formic acid and loaded into a Bakerbond SPE column pre-conditioned with 1 mL of methanol and 2 mL of 1 M formic acid. After sample application, the columns were washed with 2 mL of 1 M formic acid, and the retained analytes were eluted with 0.5 mL of 80% (*v*/*v*) methanol. The elutes were then lyophilised and suspended in 100 μL of 35% (*v*/*v*) methanol with 0.1% formic acid (*w*/*v*) and analysed using LC–MS/MS.

The analysis was performed on a Shimadzu Nexera XR UHPLC system (Shimadzu, Kyoto, Japan) with an Ascentis Express C-18 column (2.7 μm, 100 × 2.1 mm, Supelco, Bellefonte, PA, USA) as a stationary phase that was kept at 35 °C. The mobile phase consisted of (A) 0.1% formic acid and (B) methanol with 0.1% formic acid delivered as a binary gradient at a flow rate of 0.4 mL min^−1^. The gradient started at 35% B, then was raised linearly to 90% B over the next 4 min and then to 100% B over the next 2 min. The LC was interfaced with a triple quadrupole mass spectrometer (LCMS-8045, Shimadzu) operated in the positive and negative modes, with the electrospray as the ionisation source. Multiple-reaction monitoring (MRM) acquisition was carried out by monitoring the 211.3/133.30 *m*/*z* and 214.10/134.30 *m*/*z* transitions for JA and d_5_-JA, respectively, 137.30/93.00 and 141.40/97.10 for SA and d_4_-SA, 263.00/153.00 and 269.20/159.05 for ABA and d_6_-ABA, 176.20/130.30 and 178.20/132.30 for IAA and d_2_-IAA and, finally, 315.10/177.05 for pisatin (Arman, 2011). The peak area of the diagnostic product ions under optimised conditions was used for the phytohormone quantification as opposed to the peak area of the ions in the appropriate internal standards. Data acquisition and processing was performed with LabSolutions software 5.8 (Shimadzu, Kyoto, Japan).

### 4.6. Determination of Sucrose, Glucose and Total Soluble Sugar Contents

For determination of the sucrose and glucose contents in the samples, a mass spectrometer coupled to a liquid chromatographer (UPLC-MS/MS) was used [72]. About 100 mg of pea leaves or roots (samples) were homogenised in liquid nitrogen and then with 1.5 mL of 50% (*v*/*v*) of ethanol. The samples were then agitated for 2 h at 10 °C and centrifuged at 20,000× *g,* and 0.5 µL of each supernatant was injected into the system. The range used for sucrose and glucose was from 1 to 100 μg ml^–1^. The results were expressed as the mean and standard deviation of three replicates in mg g^–1^ of fresh weight. The LC-MS/MS analyses were performed using a Shimadzu Nexera XR UHPLC/LCMS-8045 system (Kyoto, Japan) with an Agilent Poroshell 120 HILIC-Z (Agilent) 2.1 × 100 mm, 2.7 μm column, maintained at 35 °C. The mobile phase was a mixture of (A) 0.1% ammonium hydroxide in water and (B) 0.1% ammonium hydroxide acetonitrile applied at a flow rate of 0.35 mL min^–1^ in an 11 min gradient as follows: 0–4 min, 85–60% B; 4–6 min, 60% B; 6–11 min, 85% B. The analytes were ionised in negative mode as [M-H]−. The mass spectrometer was operated under multiple-reaction monitoring (MRM) mode. The MRM transitions used for sugar quantitation were as follows: glucose and fructose: 179.10/89.00; sucrose: 341.00/88.90. The MS parameters for each analyte and analogue were optimised separately via the direct infusion of individual standard solutions.

The total soluble sugar content, both in the leaves and roots of pea seedlings (at least 500 mg), was determined using the anthrone method, introduced by Björnesjö in 1955 [73]. This method involves the dehydration of carbohydrates under the influence of concentrated sulfuric acid (H_2_SO_4_). As a result of this reaction, furfural derivatives are formed, which bind with anthrone, forming a green-blue complex. During the determinations, 0.020% anthrone reagent in concentrated H_2_SO_4_ was used. After preparing the anthrone reagent, 0.5 g of plant tissue was ground in a mortar with 5 cm^3^ of distilled water. The resulting homogenate was centrifuged for 20 min at 10,000× *g*, and the supernatant was poured off. Then, 1 mL of anthrone reagent was added using an automatic pipette to glass test tubes placed in a stand in an ice bath. In the next stage, 0.5 mL of the supernatant, diluted 5–10 times, was added to the test tubes. The rack with the test tubes was placed in a water bath at 90 °C for 15 min. During this time, the tests were shaken several times. The samples were then cooled in an ice bath, and their absorbance was measured using a Perkin Elmer Lambda 11 spectrophotometer at a wavelength of λ = 620 nm. The reference was a sample containing 0.5 mL of distilled water and 1 mL of anthrone reagent. The carbohydrate content in individual samples was read on the basis of a standard curve prepared for glucose and calculated to 1 g of fresh tissue weight.

### 4.7. Extraction and Activity Assay of Invertases

Invertases (EC 3.2.7.26) were extracted at 4 °C using 50 mM sodium phosphate buffer, pH 7.4 (2 mL per 500 mg of tissue), containing 1 mM mercaptoethanol. The samples were ground in a mortar and centrifuged at 15,000× *g* for 30 min according to Copeland (1990) [64] with modifications). The invertase activity was determined using a modification of the method of King et al. [65]. Soluble acid and alkaline invertases were measured via the incubation of 100 mL of extract with 100 mM sucrose in 100 mM sodium acetate acetic acid, pH 5.0 (acid invertase), or 100 mM sodium acetate acetic acid, pH 7.5 (alkaline invertase), in a total volume of 600 mL. The reactions were started by the addition of extract and incubated at 30 °C for 30 min. The reactions were stopped with 600 mL of 0.5 M Tricine-KOH, pH 8.3. Next, 850 mL of 0.14% (*w*/*v*) anthrone in 80% (*v*/*v*) H_2_SO_4_ was added. The resulting 5-hydroxymethylfurfural, formed from hexoses, creates a coloured solution with anthrone. The intensity of this coloration was determined using a PerkineElmer Lambda 11 spectrophotometer at a wavelength of λ = 620 nm. The protein content was assayed according to Bradford [74].

### 4.8. Extraction of Total RNA and Analysis Using Reverse Transcription Polymerase Chain Reaction (RT-qPCR)

The extraction of the total RNA from roots and leaves was carried out using an miRNeasy Mini Kit (Qiagen, Venlo, the Netherlands), with the additional step of on-column digestion using an RNase-Free DNase Set (Qiagen, Venlo, the Netherlands). The evaluation of the quality and quantity of the extracted total RNA was performed using a Nanodrop ND-1000 Spectrophotometer (Thermo Scientific Waltham, MA, USA), UV/VIS measurement and gel electrophoresis techniques. The synthesis of cDNA was performed with an NG dART RT Kit (EURx, Gdansk, Poland) using 0.5 µg of total RNA in a 20 µL reaction volume. The synthesised samples were then diluted with RNase-free water five times to a final volume of 100 µL before being used in the qPCR. Quantitative PCR was performed using a SensiFAST Probe No-ROX kit (Bioline Meridian Bioscience, Cincinnati, OH, USA) following the manufacturer’s protocol. Each 20 µL reaction contained the following: 5 µL of cDNA template, 1 µL of 10 µM qPCR forward primer, 1 µL of 10 µM reverse primer, 10 µL of 2× SensiFAST Probe No-ROX Mix, 0.2 µL of 10 µM specific UPL probe (Roche, Basel, Switzerland) and 2.8 µL of ddH_2_O. The qPCR was carried out on a LightCycler480 (Roche, Switzerland) under the following conditions: pre-incubation at 95 °C for 10 min followed by 45 cycles of 95 °C for 10 s, 60 °C for 30 s and 72 °C for 1 s. Each experiment, consisting of three biological replicates, was carried out in three technical replicates. The calculation of the relative expression levels was achieved using the 2^−∆∆Ct^ method, and the data were normalised to the CT values using the *PPA2* gene as a reference. The primers and UPL (Universal Probe Library) probes used in the qPCR were designed using ProbeFinder version 2.48 (Roche, Switzerland) and are listed in Table 2.

### 4.9. Statistical Analysis

The normality of the distribution of the 16 traits, e.g., length of the epicotyl, fresh weight, semiquinone radical, Mn^2+^ ions, ABA, IAA, JA, SA, pisatin, glucose, sucrose, acid invertase, alkaline invertase and carbohydrates, was tested using Shapiro–Wilk’s normality test [75] to verify whether the analysis of variance (ANOVA) met the assumption that the ANOVA model residuals followed a normal distribution [76,77,78]. The homogeneity of variance was tested using Bartlett’s test. Box’s M test tested the multivariate normality and homogeneity of the variance–covariance matrices. All the traits had a normal distribution. Two-way analyses of variance (ANOVAs) were carried out to determine the effects of the time and variant as well as the time × variant interaction on the variability of the observed traits independently for the roots and leaves. The relationships between the 14 observed traits were estimated using Pearson’s linear correlation coefficients independently for the roots and leaves. Relationships of the observed traits were presented using heatmaps [68]. In addition, the relationships between the values of the individual traits in the roots and leaves were examined using correlation analysis. The elementary comparisons between particular levels of the analysed factors were tested using the two-sample t-test for equal means [69,70] for all the observed traits, independently for the roots and leaves. To account for multiple testing, we used the Bonferroni correction. All analyses were performed independently for the leaves and roots. All analyses were performed using the GenStat v. 23 statistical software package [79].

## 5. Conclusions

**Figure 8:**
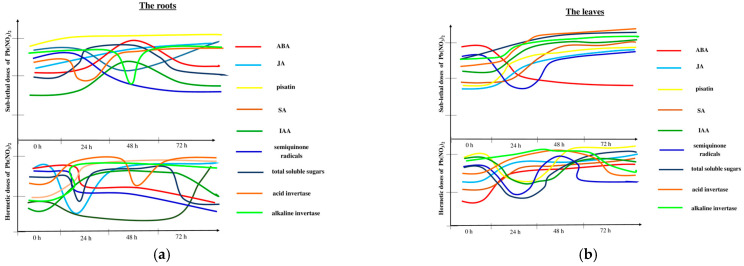
The model of the response of pea (*P. sativum* L.cv. Cysterski) seedling organs ((**a**), for the roots and (**b**), for the leaves) growing on hormetic doses or sublethal doses of lead and colonised by pea aphids (*A. pisum*) from 0 to 72 hpi.

Changes in the molecular and metabolic responses of pea seedlings depend on the duration of exposure to the stress factor, direct contact of the stress factor with the organ and its concentration (Figure 8; Table 3). We have demonstrated that Pb at a low concentration caused the hormesis effect, which was visible through the stimulated growth of the epicotyl. In turn, Pb at high concentrations caused a sublethal effect, which was manifested by the limitation of growth. The level of semiquinone radicals, paramagnetic Mn^2+^ ions and the main defence-related phytohormones, such as ABA, JA and SA, depended on the dose of Pb and the impact of a single stress factor or the combined impact of two stress factors. Pea aphid infestation caused signal induction and its transfer from leaves to roots. Additionally, pisatin levels increased under the influence of the sublethal doses of Pb and as a result of cross-talk between Pb and pea aphid infestation. This result was related to the high sucrose and glucose contents in the variants with sublethal doses. The high invertase activities in the hormetic dose variants and the high glucose levels at early time points indicate that this monosaccharide may be involved in the plant defence response against pea aphid feeding. In the present study, we also revealed a significant induction of the expression of genes encoding enzymes for the biosynthesis of pisatin, such as IFS and HMM. It should be emphasised that in the roots of pea seedlings, a sublethal dose of Pb and cross-talk between a sublethal dose of Pb and aphid infestation strongly upregulated the tested genes. In turn, in the leaves of pea seedlings from the hormetic and sublethal doses of Pb as well as during aphid feeding, high levels of transcripts for *IFS* and *HMM* were demonstrated. The results of our analyses presented in this study will improve understanding of plant–aphid interactions under various heavy metal (Pb ) contamination levels.

## Figures and Tables

**Table 1:** Effects of lead and *A. pisum* on the length and fresh weight of the roots and epicotyls of pea seedlings. The data were obtained in three independent experiments and statistically analysed using ANOVA (*p*-values at α = 0.05).

Time	Variant	Length						Fresh Weight					
		Epicotyl			Roots			Epicotyl			Roots		
		Mean		SE	Mean		SE	Mean		SE	Mean		SE
0 h	Control	6.58	ab *	0.363	11.2	a	0.276	0.415	a	0.1017	0.36	b	0.0011
	0.025 mM Pb (NO_3_)_2_	7.06	a	0.027	10.94	ab	0.375	0.455	a	0.0134	0.374	b	0.0006
	0.050 mM Pb (NO_3_)_2_	6.7	ab	0.176	10.76	ab	0.796	0.418	a	0.001	0.365	b	0.0202
	0.0625 mM Pb (NO_3_)_2_	6.64	ab	0.461	11.23	a	0.424	0.452	a	0.0239	0.411	a	0.0133
	0.1 mM Pb (NO_3_)_2_	6.09	b	0.137	10.51	ab	0.518	0.425	a	0.0353	0.318	c	0.0123
	0.25 mM Pb (NO_3_)_2_	6.29	b	0.018	9.53	b	0.08	0.385	a	0.0018	0.234	d	0.0056
	0.325 mM Pb (NO_3_)_2_	6.34	ab	0.104	7.51	c	0.468	0.351	a	0.0385	0.15	e	0.0136
LSD_0.05_		0.73			1.42			0.13			0.035		
24 h	Control	7.62	ab	0.092	11.69	abc	0.009	0.49	abc	0.0107	0.383	ab	0.0203
	0.025 mM Pb (NO_3_)_2_	7.87	a	0.379	12.03	ab	0.323	0.541	a	0.0402	0.408	a	0.0665
	0.050 mM Pb (NO_3_)_2_	8.11	a	0.134	11.99	ab	0.289	0.568	a	0.0349	0.408	a	0.032
	0.0625 mM Pb (NO_3_)_2_	7.78	a	0.067	12.47	a	0.365	0.512	ab	0.0161	0.415	a	0.0393
	0.1 mM Pb (NO_3_)_2_	7.78	a	0.273	11.57	abc	0.109	0.471	abcd	0.0322	0.36	abc	0.0325
	0.25 mM Pb (NO_3_)_2_	7.39	abc	0.557	10.4	bc	0.258	0.442	bcd	0.0265	0.294	c	0.0002
	0.325 mM Pb (NO_3_)_2_	6.53	cd	0.584	6.55	e	0.286	0.376	de	0.0341	0.145	d	0.0144
	Control + aphids	7.51	ab	0.309	11.7	abc	0.907	0.477	abc	0.009	0.347	abc	0.0078
	0.025 mM Pb (NO_3_)_2_ + aphids	7.41	abc	0.292	12.43	a	0.888	0.474	abc	0.0071	0.326	bc	0.0075
	0.050 mM Pb (NO_3_)_2_ + aphids	8.27	a	0.522	12.34	a	0.5	0.526	ab	0.0088	0.368	abc	0.0072
	0.0625 mM Pb (NO_3_)_2_ + aphids	7.7	a	0.319	12.92	a	0.861	0.492	abc	0.0302	0.358	abc	0.0262
	0.1 mM Pb (NO_3_)_2_ + aphids	7.44	abc	0.196	11.43	abc	0.827	0.412	cde	0.0838	0.316	bc	0.0136
	0.25 mM Pb (NO_3_)_2_ + aphids	6.71	bcd	0.034	10.09	c	0.646	0.401	cde	0.0326	0.294	c	0.0136
	0.325 mM Pb (NO_3_)_2_ + aphids	5.81	d	0.062	6.59	d	0.693	0.335	e	0.0209	0.127	d	0.0088
LSD_0.05_		0.95			1.672			0.097			0.077		
48 h	Control	8.45	a	0.08	12.37	a	0.225	0.591	bc	0.0061	0.406	abc	0.0095
	0.025 mM Pb (NO_3_)_2_	8.87	a	0.263	12.6	a	0.089	0.63	ab	0.0277	0.434	abc	0.0177
	0.050 mM Pb (NO_3_)_2_	9.32	a	0.019	12.88	a	0.299	0.67	a	0.0623	0.455	a	0.0482
	0.0625 mM Pb (NO_3_)_2_	9.38	a	0.075	13.17	a	0.195	0.625	ab	0.0276	0.45	ab	0.0448
	0.1 mM Pb (NO_3_)_2_	8.51	a	0.025	11.68	ab	0.298	0.544	cd	0.0061	0.383	c	0.0046
	0.25 mM Pb (NO_3_)_2_	8.3	ab	0.188	9.75	bc	0.552	0.482	de	0.0262	0.265	d	0.0199
	0.325 mM Pb (NO_3_)_2_	6.87	b	0.521	5.53	d	0.027	0.362	g	0.0029	0.113	e	0.0069
	Control + aphids	8.07	ab	0.769	12.64	a	1.247	0.583	bc	0.0136	0.395	abc	0.0146
	0.025 mM Pb (NO_3_)_2_ + aphids	8.51	a	0.25	12.71	a	0.522	0.596	abc	0.0136	0.413	abc	0.0136
	0.050 mM Pb (NO_3_)_2_ + aphids	8.68	a	0.659	13.25	a	0.888	0.594	abc	0.0325	0.396	abc	0.0071
	0.0625 mM Pb (NO_3_)_2_ + aphids	8.99	a	0.933	13.41	a	1.193	0.591	bc	0.0209	0.395	abc	0.0208
	0.1 mM Pb (NO_3_)_2_ + aphids	8.27	ab	0.764	11.32	ab	0.927	0.536	cd	0.0136	0.389	bc	0.0069
	0.25 mM Pb (NO_3_)_2_ + aphids	8.26	ab	0.483	9.08	c	1.474	0.444	ef	0.0382	0.289	d	0.0188
	0.325 mM Pb (NO_3_)_2_ + aphids	6.79	b	0.917	5.5	d	0.744	0.366	fg	0.0205	0.122	e	0.0072
LSD_0.05_		1.55			2.22			0.078			0.063		
72 h	Control	9.92	a	0.62	13.66	a	0.298	0.656	a	0.0137	0.426	ab	0.0291
	0.025 mM Pb (NO_3_)_2_	9.52	ab	0.7	13.35	a	0.556	0.656	a	0.0007	0.436	a	0.0011
	0.050 mM Pb (NO_3_)_2_	9.98	a	0.717	14.59	a	0.119	0.703	a	0.0387	0.447	a	0.007
	0.0625 mM Pb (NO_3_)_2_	10.19	a	0.95	14.35	a	0.54	0.671	a	0.2268	0.451	a	0.0237
	0.1 mM Pb (NO_3_)_2_	9.84	ab	0.401	12.5	ab	0.365	0.637	a	0.0258	0.389	bc	0.0087
	0.25 mM Pb (NO_3_)_2_	9.25	ab	1.017	10.88	b	0.14	0.582	ab	0.0045	0.318	d	0.0126
	0.325 mM Pb (NO_3_)_2_	8.23	ab	0.8	6.19	c	0.35	0.51	ab	0.0677	0.149	e	0.0202
	Control + aphids	9.82	ab	1.55	13.29	a	1.422	0.63	a	0.0211	0.391	bc	0.0072
	0.025 mM Pb (NO_3_)_2_ + aphids	9.85	ab	0.918	12.67	ab	1.549	0.641	a	0.0321	0.437	a	0.0088
	0.050 mM Pb (NO_3_)_2_ + aphids	9.96	a	0.687	14.44	a	0.809	0.653	a	0.0084	0.432	ab	0.0136
	0.0625 mM Pb (NO_3_)_2_ + aphids	10.1	a	0.587	14.77	a	0.729	0.699	a	0.0185	0.422	abc	0.0072
	0.1 mM Pb (NO_3_)_2_ + aphids	9.87	ab	0.585	12.95	ab	0.954	0.605	ab	0.0377	0.379	c	0.0072
	0.25 mM Pb (NO_3_)_2_ + aphids	9.02	ab	0.68	10.71	b	0.598	0.576	ab	0.0188	0.334	d	0.0208
	0.325 mM Pb (NO_3_)_2_ + aphids	7.59	b	0.115	6.65	c	1.027	0.427	b	0.0301	0.124	e	0.0148
LSD_0.05_		2.32			2.31			0.194			0.044		

* In the columns, for individual times, the means denoted by the same letters are not significantly different. SE—standard error.

**Table 2:** List of genes, primers and UPL probes used for RT-qPCR analysis.

Gene Symbol	Gene Name	Sequence Accession Number (NCBI GenBank)	EC Number	Forward Primer	Reverse Primer	UPL Probe No
Genes encoding pisatin biosynthesis						
*IFS*	*Isoflavone synthase*	AF532999.2	1.14.14.87	caagggtcttgttgtggatttct	tggcagagctgatcaacaatcc	88
*HMM*	*6α-hydroxymaackiain 3-O-methyltransferase*	U69554.1	2.1.1.270	gtcccttctgctgatgctgt	agaagcaatttcacacaaaggga	22
*Reference gene*						
*PP2A*	*Phosphoprotein phosphatase 2A*	Z25888	3.1.3.16	agctctgtgaagctgttggtc	cgaacatatgcaggaaccaat	31

**Table 3:** Expression of molecular and metabolic parameters in (a) roots and (b) leaves of pea seedlings (*P. sativum*). Arrows show an upward or downward trend (blue for hormetic dose; red for sublethal dose).

The Roots																
	Parameter	Time After Infestation	Semiquinone Radicals	Mn^2+^ Ions	IFS Genes	HMM Genes	ABA	SA	JA	IAA	Sucrose	Glucose	Total Soluble Sugars	Pisatin	Acid Invertase	Alkaline Invertase
Variant																
+aphids		24 h	↑	↑	↓	↓	↓	↑	↓	↑	↓	↑	↓	↑	↓	↓
		48 h	↑	↑	↑	↓	↓	↓	↑	↑	↑	↓	↑	↑	↓	↓
		72 h	↓	↑	↓	↑	↓	↓	↑	↓	↑	↑	↓	↓	↓	↓
+hormetic doses		0h	↓	↓	↓	↓	↑	↓	↑	↓	↓	↑	↑	↑	↑	↓
		24 h	↓	↓	↓	↓	↓	↓	↓	↑	↑	↑	↑	↑	↑	↑
		48 h	↑	↑	↓	↓	↓	↑	↑	↑	↑	↑	↑	↓	↓	↑
		72 h	↓	↑	↓	↓	↓	↓	↑	↓	↑	↑	↑	↓	↑	↑
+hormetic doses +aphids		24 h	↓	↓	↓	↓	↓	↓	↓	↑	↑	↓	↓	↑	↑	↑
		48 h	↓	↑	↓	↓	↓	↓	↑	↑	↑	↑	↑	↑	↓	↑
		72 h	↓	↑	↓	↓	↓	↑	↑	↓	↑	↑	↓	↑	↑	↑
+sublethal doses		0 h	↑	↓	↑	↑	↑	↑	↑	↓	↑	↑	↑	↑	↑	↑
		24 h	↑	↓	↑	↑	↑	↑	↑	↑	↑	↓	↑	↑	↑	↓
		48 h	↓	↓	↑	↑	↑	↑	↓	↑	↑	↑	↑	↑	↓	↓
		72 h	↓	↓	↑	↑	↑	↑	↑	↑	↑	↑	↓	↑	↑	↑
+sublethal doses +aphids		24 h	↑	↓	↑	↑	↑	↑	↑	↑	↑	↑	↑	↑	↓	↑
		48 h	↓	↓	↑	↑	↑	↓	↑	↑	↑	↑	↑	↑	↑	↓
		72 h	↓	↓	↑	↑	↓	↑	↑	↓	↑	↑	↓	↑	↑	↑
**The Leaves**																
	**Parameter**	**Time After Infestation**	**Semiquinone Radicals**	**Mn^2+^ Ions**	**IFS Genes**	**HMM Genes**	**ABA**	**SA**	**JA**	**IAA**	**Sucrose**	**Glucose**	**Total Soluble Sugars**	**Pisatin**	**Acid Invertase**	**Alkaline Invertase**
**Variant**																
+aphids		24 h	↑	↑	↑	↑	↓	↑	↑	↓	↓	↑	↓	↑	↓	↓
		48 h	↓	↓	↑	↑	↓	↑	↑	↓	↓	↓	↑	↑	↑	↑
		72 h	↓	↓	↑	↑	↓	↑	↑	↑	↑	↑	↑	↓	↓	↓
+hormetic doses		0h	↑	↑	↓	↓	↓	↓	↑	↑	↑	↑	↑	↑	↑	↑
		24 h	↑	↓	↑	↑	↓	↑	↑	↓	↑	↑	↓	↑	↑	↑
		48 h	↓	↓	↑	↑	↓	↑	↑	↑	↑	↓	↑	↑	↑	↑
		72 h	↓	↑	↑	↑	↑	↑	↑	↑	↑	↑	↑	↑	↑	↑
+hormetic doses +aphids		24 h	↓	↓	↑	↑	↑	↑	↑	↓	↓	↑	↓	↑	↑	↑
		48 h	↑	↑	↑	↑	↓	↑	↑	↑	↓	↓	↑	↑	↑	↑
		72 h	↓	↓	↑	↑	↓	↑	↑	↑	↑	↑	↑	↓	↓	↓
+sublethal doses		0 h	↑	↓	↑	↑	↑	↑	↑	↑	↑	↑	↑	↑	↑	↑
		24 h	↓	↓	↑	↑	↑	↑	↑	↑	↑	↑	↑	↑	↑	↑
		48 h	↓	↓	↑	↑	↑	↑	↑	↑	↑	↑	↑	↑	↑	↓
		72 h	↓	↓	↑	↑	↓	↑	↑	↑	↑	↑	↑	↑	↓	↑
+sublethal doses +aphids		24 h	↓	↑	↑	↑	↓	↑	↑	↑	↑	↑	↑	↑	↑	↑
		48 h	↓	↑	↑	↑	↓	↑	↑	↑	↑	↑	↑	↑	↑	↑
		72 h	↓	↓	↑	↑	↓	↑	↑	↑	↑	↑	↑	↑	↑	↑

## Data Availability

Data supporting the results are deposited with the first author and corresponding author of the manuscript.

## Supplementary Materials

The following supporting information can be downloaded at: https://www.mdpi.com/article/10.3390/ijms252111804/s1.
