# Supplementary material for: The Effects of Lead and Cross-Talk Between Lead and Pea Aphids on Defence Responses of Pea Seedlings
Source: Int J Mol Sci. 2024 Nov 2;25(21):11804. doi: 10.3390/ijms252111804 (PMC11546482; doi:10.3390/ijms252111804)
Supplement: Supplementary file 1 [file ijms-25-11804-s001.zip › ijms-3230044-supplementary.pdf]

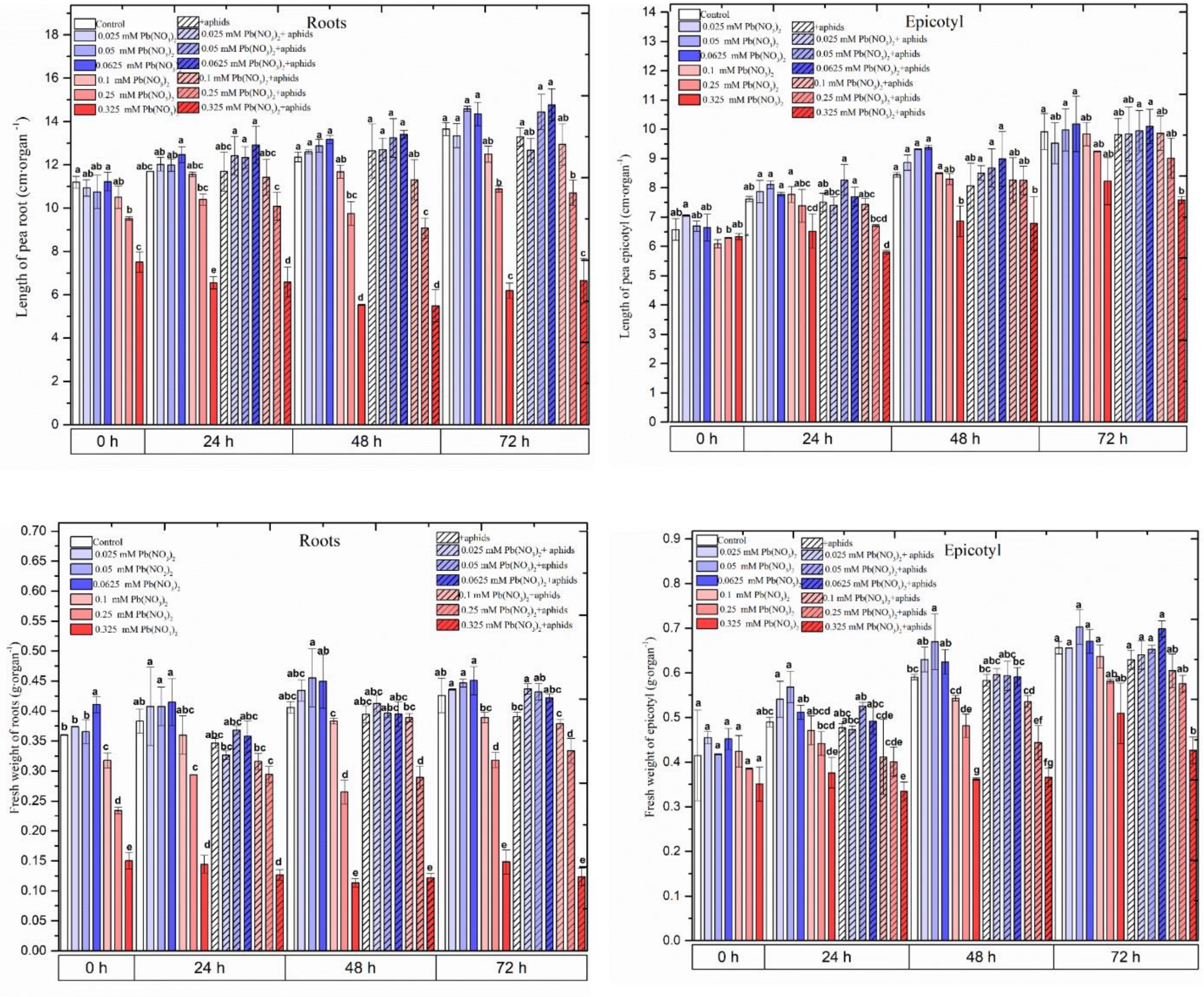

Figure S1. The length and fresh weight of the roots and the epicotyls of pea seedlings growing on the Hoagland medium with varied concentrations of Pb, i.e. 0.025 $Pb(NO_3)_2$, 0.05 $Pb(NO_3)_2$, 0.0625 $Pb(NO_3)_2$, 0.1 $Pb(NO_3)_2$, 0.25 $Pb(NO_3)_2$ and 0.325 $Pb(NO_3)_2$, and pea seedlings growing on the Hoagland medium with varied concentrations of Pb and colonised by pea aphids, *A. pisum*, and pea seedlings growing on the Hoagland medium colonised by pea aphids, *A. pisum*. The data were obtained in three independent experiments and statistically analysed using ANOVA (p-values at $\alpha = 0.05$).

Table S1. Comparisons between particular levels of analyzed factor in the roots of pea seedlings in different times (independently) using the two-sample *t*-test for equal means for all observed traits.

Contrasts		Length of epicotyl	Fresh weight	Semiquinone radical	Mn^{2+}	ABA	IAA	JA	SA	Pisatin	Glucose	Sucrose	Acid invertase	Alkaline/neutral invertase	Total soluble sugar
0 h	control vs. 0.025mM Pb2+	0.26	-0.014	0.11**	-0.5	-0.24	4.3**	-81.3***	-0.6	-0.53	-0.52***	0.017	-0.001066***	0.00002	-0.00006
0 h	control vs. 0.05mM Pb2+	0.44	-0.005	0.1**	-1.6**	0.34	-0.1	-68.9***	-1.9	-0.61	-1.2***	0	-0.001536***	0.00005	-0.00013
0 h	control vs. 0.0625mM Pb2+	-0.03	-0.051	0.18***	3.3***	-0.14	3.7*	-75.4***	1.3	-0.06	-0.24*	0.016	-0.001676***	-0.00012	-0.00003
0 h	control vs. 0.1 mM Pb2+	0.69	0.043	0.17***	0.1	-1.92***	4.7**	-123.5***	-1.9	-0.21	-0.03	-0.129***	-0.000816***	0	-0.00091***
0 h	control vs. 0.25 mM Pb2+	1.67	0.126***	0.01	4.1***	-1.46***	4.6**	-119.5***	-7**	-4.7***	-0.6***	-0.06***	-0.000946***	-0.00069***	-0.00108***
0 h	control vs. 0.325 mM Pb2+	3.69***	0.21***	-0.05	4.5***	-4.59***	1.3	-185.5***	-32***	-18.27***	0.02	-0.181***	-0.000273***	-0.00029*	-0.00101***
0 h	0.025mM Pb2+vs 0.05mM Pb2+	0.18	0.008	-0.01	-1.1*	0.58	-4.4**	12.4	-1.3	-0.08	-0.68***	-0.017	-0.00047***	0.00003	-0.00006
0 h	0.025mM Pb2+vs 0.0625mM Pb2+	-0.29	-0.037	0.07*	3.8***	0.09	-0.6	5.9	1.9	0.47	0.27**	-0.001	-0.00061***	-0.00014	0.00003
0 h	0.025mM Pb2+vs 0.1 mM Pb2+	0.43	0.056	0.06	0.6	-1.69***	0.4	-42.2***	-1.3	0.32	0.49***	-0.146***	0.00025***	-0.00001	-0.00085***
0 h	0.025mM Pb2+ vs. 0.25 mM Pb2+	1.41	0.14***	-0.1**	4.6***	-1.22**	0.3	-38.2***	-6.4*	-4.17***	-0.09	-0.077***	0.00012*	-0.00071***	-0.00102***
0 h	0.025mM Pb2+ vs. 0.325 mM Pb2+	3.43***	0.224***	-0.16***	5***	-4.35***	-3*	-104.2***	-31.4***	-17.74***	0.54***	-0.199***	0.000793***	-0.00031*	-0.00095***
0 h	0.05mM Pb2+vs 0.0625mM Pb2+	-0.48	-0.045	0.08*	4.9***	-0.48	3.8*	-6.5	3.2	0.55	0.95***	0.016	-0.00014*	-0.00017	0.00009
0 h	0.05mM Pb2+ vs 0.1 mM Pb2+	0.24	0.048	0.07*	1.7**	-2.26***	4.8**	-54.6***	0	0.4	1.17***	-0.13***	0.00072***	-0.00004	-0.00079***
0 h	0.05mM Pb2+ vs. 0.25 mM Pb2+	1.22	0.131***	-0.09*	5.7***	-1.8***	4.7**	-50.6***	-5.1*	-4.09***	0.59***	-0.061***	0.00059***	-0.00074***	-0.00095***
0 h	0.05mM Pb2+ vs. 0.325 mM Pb2+	3.25***	0.215***	-0.15***	6.1***	-4.93***	1.4	-116.6***	-30.1***	-17.67***	1.22***	-0.182***	0.001263***	-0.00034*	-0.00088***
0 h	0.0625mM Pb2+vs 0.1 mM Pb2+	0.72	0.093**	-0.01	-3.2***	-1.78***	1	-48.1***	-3.2	-0.15	0.21*	-0.146***	0.00086***	0.00012	-0.00088***
0 h	0.0625mM vs. 0.25 mM Pb2+	1.7	0.177***	-0.17***	0.8	-1.32***	0.9	-44.1***	-8.3***	-4.64***	-0.36***	-0.077***	0.00073***	-0.00057***	-0.00104***
0 h	0.0625mM vs. 0.325 mM Pb2+	3.72***	0.261***	-0.23***	1.2*	-4.44***	-2.4	-110.1***	-33.3***	-18.22***	0.26*	-0.198***	0.001403***	-0.00017	-0.00097***
0 h	0.1 mM Pb2+ vs 0.25 mM Pb2+	0.98	0.083**	-0.16***	4***	0.46	0	4	-5.1*	-4.49***	-0.57***	0.069***	-0.00013*	-0.00069***	-0.00017
0 h	0.1 mM Pb2+ vs 0.325 mM Pb2+	3**	0.167***	-0.22***	4.4***	-2.66***	-3.4*	-62***	-30.1***	-18.07***	0.05	-0.052**	0.000543***	-0.0003*	-0.0001
0 h	0.25 mM Pb2+vs 0.325 mM Pb2+	2.02*	0.084**	-0.06	0.4	-3.13***	-3.3*	-66***	-25***	-13.58***	0.62***	-0.121***	0.000673***	0.00039**	0.00007
24 h	control vs. 0.025mM Pb2+	-0.34	-0.025	-0.14***	-7.4***	-0.16	-1.6	113.6***	-38.1***	-0.25	0.13	-0.012	-0.000614***	-0.00644***	-0.00056***
24 h	control vs. 0.05mM Pb2+	-0.3	-0.025	0.02	6***	4.04***	-7.1***	55.3***	-16.6***	-0.07	-1.16***	-0.02	-0.000377***	-0.00284***	-0.00055***
24 h	control vs. 0.0625mM Pb2+	-0.79	-0.032	0.03	8.9***	1.49***	-8.3***	175.8***	9.9***	-0.58	-0.57***	0.001	-0.000434***	-0.01258***	-0.00069***
24 h	control vs. 0.1 mM Pb2+	0.12	0.023	0.07*	7.2***	2.11***	-22.4***	-32.1***	-59.3***	-0.06	-0.66***	-0.072***	-0.000366***	0.0013***	-0.00064***
24 h	control vs. 0.25 mM Pb2+	1.29	0.089**	-0.05	9.3***	0.57	-19.3***	-353.6***	-55.2***	0.07	0.14	-0.022	-0.001044***	0.00441***	-0.00064***
24 h	control vs. 0.325 mM Pb2+	5.13***	0.238***	-0.17***	9.3***	-4.85***	-38.5***	-375***	-21.9***	-4.17***	0.1	-0.182***	-0.000075	0.00452***	-0.00102***
24 h	0.025mM Pb2+vs 0.05mM Pb2+	0.04	0	0.16***	13.4***	4.2***	-5.5***	-58.3***	21.6***	0.18	-1.28***	-0.008	0.000237***	0.0036***	0.00001
24 h	0.025mM Pb2+vs 0.0625mM Pb2+	-0.45	-0.007	0.17***	16.3***	1.65***	-6.7***	62.3***	48***	-0.33	-0.69***	0.013	0.00018**	-0.00614***	-0.00013
24 h	0.025mM Pb2+vs 0.1 mM Pb2+	0.46	0.048	0.21***	14.6***	2.26***	-20.8***	-145.7***	-21.2***	0.19	-0.79***	-0.059***	0.000247***	0.00774***	-0.00008
24 h	0.025mM Pb2+ vs. 0.25 mM Pb2+	1.63	0.114***	0.09*	16.7***	0.73	-17.6***	-467.1***	-17.1***	0.32	0.02	-0.01	-0.00043***	0.01085***	-0.00008
24 h	0.025mM Pb2+ vs. 0.325 mM Pb2+	5.47***	0.263***	-0.03	16.7***	-4.7***	-36.9***	-488.5***	16.2***	-3.92***	-0.03	-0.169***	0.000538***	0.01096***	-0.00046***
24 h	0.05mM Pb2+vs 0.0625mM Pb2+	-0.48	-0.007	0.01	2.9***	-2.55***	-1.2	120.6***	26.5***	-0.51	0.59***	0.021	-0.000057	-0.00974***	-0.00014
24 h	0.05mM Pb2+ vs 0.1 mM Pb2+	0.42	0.048	0.05	1.2*	-1.94***	-15.2***	-87.4***	-42.8***	0.01	0.49***	-0.052**	0.00001	0.00414***	-0.00009
24 h	0.05mM Pb2+ vs. 0.25 mM Pb2+	1.59	0.114***	-0.07*	3.3***	-3.47***	-12.1***	-408.8***	-38.6***	0.14	1.3***	-0.002	-0.000667***	0.00725***	-0.00009
24 h	0.05mM Pb2+ vs. 0.325 mM Pb2+	5.44***	0.263***	-0.19***	3.3***	-8.89***	-31.4***	-430.2***	-5.3*	-4.1***	1.25***	-0.162***	0.000301***	0.00736***	-0.00047***

24 h	0.0625mM Pb2+vs 0.1 mM Pb2+	0.91	0.055	0.04	-1.7**	0.61	-14.1***	-207.9***	-69.3***	0.52	-0.1	-0.073***	0.000067	0.01388***	0.00005
24 h	0.0625mM vs. 0.25 mM Pb2+	2.07*	0.121***	-0.08*	0.4	-0.92*	-10.9***	-529.4***	-65.1***	0.65	0.71***	-0.023	-0.00061***	0.01699***	0.00005
24 h	0.0625mM vs. 0.325 mM Pb2+	5.92***	0.27***	-0.2***	0.4	-6.35***	-30.2***	-550.8***	-31.8***	-3.59***	0.66***	-0.183***	0.000358***	0.0171***	-0.00033**
24 h	0.1 mM Pb2+ vs 0.25 mM Pb2+	1.16	0.066*	-0.12***	2.1***	-1.53***	3.1*	-321.5***	4.1	0.12	0.81***	0.05**	-0.000677***	0.00311***	0
24 h	0.1 mM Pb2+ vs 0.325 mM Pb2+	5.01***	0.215***	-0.24***	2.1***	-6.96***	-16.1***	-342.9***	37.4***	-4.12***	0.76***	-0.11***	0.000291***	0.00322***	-0.00038**
24 h	0.25 mM Pb2+vs 0.325 mM Pb2+	3.85***	0.149***	-0.12***	0	-5.42***	-19.2***	-21.4***	33.3***	-4.24***	-0.05	-0.159***	0.000968***	0.00011	-0.00038**
24 h	control +aphids vs. 0.025mM Pb2+ +aphids	-0.73	0.02	0.13***	5.5***	-1.29***	-4.9**	98.5***	8.7***	0.26	-0.16	-0.002	-0.000572***	-0.00003	-0.00037**
24 h	control +aphids vs. 0.05mM Pb2+ +aphids	-0.64	-0.022	0.24***	9.3***	-0.38	-18.5***	77.7***	-66.6***	0.44	0.32**	-0.01	-0.001422***	-0.00102***	-0.00045***
24 h	control +aphids vs. 0.0625mM Pb2+ +aphids	-1.22	-0.011	0.16***	8.1***	-1.79***	-46.3***	18.1**	26.6***	0.2	-0.28**	-0.014	-0.001182***	-0.00472***	-0.00031*
24 h	control +aphids vs. 0.1 mM Pb2+ +aphids	0.27	0.031	0.12***	8.1***	-3.04***	-4.7**	-27.1***	-29.2***	0.2	0.07	-0.002	-0.000243***	-0.00488***	-0.00074***
24 h	control +aphids vs. 0.25 mM Pb2+ +aphids	1.61	0.052	0.05	10.8***	-1.45***	-10.3***	-463***	-7.1**	0.3	-0.04	-0.083***	-0.000203***	-0.00369***	-0.00071***
24 h	control +aphids vs. 0.325 mM Pb2+ +aphids	5.11***	0.22***	0.01	10.4***	-10.22***	-7.8***	-474.1***	-11.7***	-0.2	0.07	-0.161***	0.000222***	-0.00299***	-0.00104***
24 h	0.025mM Pb2+ +aphids vs 0.05mM Pb2+ +aphids	0.08	-0.042	0.11**	3.8***	0.92*	-13.6***	-20.7**	-75.3***	0.18	0.48***	-0.008	-0.00085***	-0.00099***	-0.00008
24 h	0.025mM Pb2+ +aphids vs 0.0625mM Pb2+ +aphids	-0.5	-0.031	0.03	2.6***	-0.49	-41.4***	-80.4***	17.9***	-0.06	-0.12	-0.012	-0.00061***	-0.00469***	0.00007
24 h	0.025mM Pb2+ +aphids vs 0.1 mM Pb2+ +aphids	0.99	0.011	-0.01	2.6***	-1.75***	0.2	-125.6***	-37.9***	-0.06	0.23*	0	0.000329***	-0.00485***	-0.00036**
24 h	0.025mM Pb2+ +aphids vs. 0.25 mM Pb2+ +aphids	2.34*	0.032	-0.08*	5.3***	-0.16	-5.4***	-561.5***	-15.8***	0.04	0.12	-0.082***	0.000369***	-0.00366***	-0.00033**
24 h	0.025mM Pb2+ +aphids vs. 0.325 mM Pb2+ +aphids	5.84***	0.2***	-0.12***	4.9***	-8.92***	-2.9	-572.5***	-20.4***	-0.46	0.23*	-0.159***	0.000794***	-0.00296***	-0.00066***
24 h	0.05mM Pb2+ +aphids vs 0.0625mM Pb2+ +aphids	-0.58	0.011	-0.08*	-1.2*	-1.41***	-27.8***	-59.7***	93.2***	-0.24	-0.6***	-0.004	0.00024***	-0.0037***	0.00014
24 h	0.05mM Pb2+ +aphids vs 0.1 mM Pb2+ +aphids	0.91	0.053	-0.12***	-1.2*	-2.67***	13.8***	-104.8***	37.4***	-0.24	-0.25*	0.008	0.001179***	-0.00386***	-0.00029*
24 h	0.05mM Pb2+ +aphids vs. 0.25 mM Pb2+ +aphids	2.26*	0.074*	-0.19***	1.5**	-1.07**	8.2***	-540.8***	59.5***	-0.14	-0.36***	-0.073***	0.001219***	-0.00267***	-0.00026*
24 h	0.05mM Pb2+ +aphids vs. 0.325 mM Pb2+ +aphids	5.75***	0.241***	-0.23***	1.1*	-9.84***	10.7***	-551.8***	54.9***	-0.64	-0.25*	-0.151***	0.001644***	-0.00197***	-0.00059***
24 h	0.0625mM Pb2+ +aphids vs 0.1 mM Pb2+ +aphids	1.49	0.042	-0.04	0	-1.26**	41.7***	-45.2***	-55.8***	0	0.35***	0.012	0.000939***	-0.00016	-0.00043***
24 h	0.0625mM +aphids vs. 0.25 mM Pb2+ +aphids	2.83**	0.064*	-0.11**	2.7***	0.34	36***	-481.1***	-33.7***	0.1	0.24*	-0.069***	0.000979***	0.00103***	-0.0004***
24 h	0.0625mM +aphids vs. 0.325 mM Pb2+ +aphids	6.33***	0.231***	-0.15***	2.3***	-8.43***	38.5***	-492.1***	-38.3***	-0.4	0.35***	-0.147***	0.001404***	0.00173***	-0.00073***
24 h	0.1 mM Pb2+ +aphids vs 0.25 mM Pb2+ +aphids	1.35	0.022	-0.07*	2.7***	1.59***	-5.7***	-435.9***	22.1***	0.1	-0.11	-0.081***	0.00004	0.00119***	0.00003
24 h	0.1 mM Pb2+ +aphids vs 0.325 mM Pb2+ +aphids	4.84***	0.189***	-0.11**	2.3***	-7.17***	-3.1*	-447***	17.5***	-0.4	0	-0.159***	0.000465***	0.00189***	-0.0003*
24 h	0.25 mM Pb2+ +aphids vs 0.325 mM Pb2+ +aphids	3.5***	0.167***	-0.04	-0.4	-8.77***	2.5	-11	-4.6	-0.5	0.11	-0.077***	0.000425***	0.0007***	-0.00033**
24 h	0.025mM Pb2+vs 0.05mM Pb2+ +aphids	-0.32	0.039	0.283***	15.5***	3.77***	-18.6***	11.2	-50.7***	0.37	-0.12	0.006	-0.00078***	0.00982***	0.00044***
24 h	0.025mM Pb2+vs 0.0625mM Pb2++aphids	-0.89	0.05	0.203***	14.3***	2.36***	-46.4***	-48.5***	42.4***	0.13	-0.73***	0.002	-0.00054***	0.00612***	0.00058***
24 h	0.025mM Pb2+vs 0.1 mM Pb2++aphids	0.6	0.092**	0.163***	14.3***	1.1**	-4.8**	-93.7***	-13.3***	0.13	-0.37***	0.014	0.000399***	0.00596***	0.00015
24 h	0.025mM Pb2+ vs. 0.25 mM Pb2++aphids	1.94*	0.114***	0.093**	17***	2.69***	-10.5***	-529.6***	8.8***	0.23	-0.48***	-0.067***	0.000439***	0.00715***	0.00018
24 h	0.025mM Pb2+ vs. 0.325 mM Pb2++aphids	5.44***	0.281***	0.053	16.6***	-6.07***	-7.9***	-540.7***	4.2	-0.27	-0.37***	-0.144***	0.000864***	0.00785***	-0.00015
24 h	0.05mM Pb2+vs 0.0625mM Pb2+ +aphids	-0.93	0.05	0.043	0.9	-1.84***	-40.9***	9.8	20.9***	-0.05	0.56***	0.01	-0.000777***	0.00252***	0.00057***
24 h	0.05mM Pb2+ vs 0.1 mM Pb2++aphids	0.56	0.092**	0.003	0.9	-3.1***	0.7	-35.4***	-34.9***	-0.05	0.91***	0.022	0.000162**	0.00236***	0.00014
24 h	0.05mM Pb2+ vs. 0.25 mM Pb2++aphids	1.9	0.113***	-0.067	3.6***	-1.5***	-4.9**	-471.3***	-12.8***	0.05	0.8***	-0.059***	0.000202***	0.00355***	0.00017
24 h	0.05mM Pb2+ vs. 0.325 mM Pb2++aphids	5.4***	0.281***	-0.107**	3.2***	-10.27***	-2.4	-482.4***	-17.4***	-0.45	0.91***	-0.137***	0.000627***	0.00425***	-0.00016
24 h	0.0625mM Pb2+vs 0.1 mM Pb2++aphids	1.04	0.099***	-0.007	-2***	-0.55	1.9	-155.9***	-61.4***	0.46	0.32**	0.001	0.000219***	0.0121***	0.00028*
24 h	0.0625mM vs. 0.25 mM Pb2++aphids	2.39*	0.121***	-0.077*	0.7	1.04**	-3.8*	-591.9***	-39.3***	0.56	0.21*	-0.08***	0.000259***	0.01329***	0.00031*
24 h	0.0625mM vs. 0.325 mM Pb2++aphids	5.88***	0.288***	-0.117***	0.3	-7.72***	-1.2	-602.9***	-43.9***	0.06	0.32**	-0.158***	0.000684***	0.01399***	-0.00002
24 h	0.1 mM Pb2+ vs 0.25 mM Pb2+ +aphids	1.48	0.066*	-0.117***	2.4***	0.43	10.3***	-384***	30***	0.04	0.31**	-0.008	0.000192***	-0.00059***	0.00026*
24 h	0.1 mM Pb2+ vs 0.325 mM Pb2++aphids	4.98***	0.233***	-0.157***	2***	-8.34***	12.8***	-395***	25.4***	-0.46	0.42***	-0.085***	0.000617***	0.00011	-0.00007
24 h	0.25 mM Pb2+vs 0.325 mM Pb2++aphids	3.81***	0.167***	-0.037	-0.1	-6.8***	9.7***	-73.5***	21.2***	-0.59	-0.39***	-0.135***	0.001294***	-0.003***	-0.00007
24 h	0.025mM Pb2++ aphids vs 0.05mM Pb2+ +aphids	0.08	-0.042	0.11**	3.8***	0.92*	-13.6***	-20.7**	-75.3***	0.18	0.48***	-0.008	-0.00085***	-0.00099***	-0.00008
24 h	0.025mM Pb2+ + aphids vs 0.0625mM Pb2++aphids	-0.5	-0.031	0.03	2.6***	-0.49	-41.4***	-80.4***	17.9***	-0.06	-0.12	-0.012	-0.00061***	-0.00469***	0.00007

24 h	0.025mM Pb2+ + aphids vs 0.1 mM Pb2++aphids	0.99	0.011	-0.01	2.6***	-1.75***	0.2	-125.6***	-37.9***	-0.06	0.23**	0	0.000329***	-0.00485***	-0.00036**
24 h	0.025mM Pb2+ + aphids vs. 0.25 mM Pb2++aphids	2.34*	0.032	-0.08*	5.3***	-0.16	-5.4***	-561.5***	-15.8***	0.04	0.12	-0.082***	0.000369***	-0.00366***	-0.00033**
24 h	0.025mM Pb2+ + aphids vs. 0.325 mM Pb2++aphids	5.84***	0.2***	-0.12***	4.9***	-8.92***	-2.9	-572.5***	-20.4***	-0.46	0.23*	-0.159***	0.000794***	-0.00296***	-0.00066***
24 h	0.05 mM Pb2+ + aphids vs 0.0625mM Pb2++ aphids	-0.58	0.011	-0.08*	-1.2*	-1.41***	-27.8***	-59.7***	93.2***	-0.24	-0.6***	-0.004	0.00024***	-0.0037***	0.00014
24 h	0.05mM Pb2+ + aphids vs 0.1 mM Pb2+ + aphids	0.91	0.053	-0.12***	-1.2*	-2.67***	13.8***	-104.8***	37.4***	-0.24	-0.25*	0.008	0.001179***	-0.00386***	-0.00029*
24 h	0.05mM Pb2+ + aphids vs. 0.25 mM Pb2++ aphids	2.26*	0.074*	-0.19***	1.5**	-1.07**	8.2***	-540.8***	59.5***	-0.14	-0.36***	-0.073***	0.001219***	-0.00267***	-0.00026*
24 h	0.05mM Pb2++ aphids vs. 0.325 mM Pb2++ aphids	5.75***	0.241***	-0.23***	1.1*	-9.84***	10.7***	-551.8***	54.9***	-0.64	-0.25*	-0.151***	0.001644***	-0.00197***	-0.00059***
24 h	0.0625mM Pb2++aphids vs 0.1 mM Pb2++aphids	1.49	0.042	-0.04	0	-1.26**	41.7***	-45.2***	-55.8***	0	0.35***	0.012	0.000939***	-0.00016	-0.00043***
24 h	0.0625mM +aphids vs. 0.25 mM Pb2++aphids	2.83**	0.064*	-0.11**	2.7***	0.34	36***	-481.1***	-33.7***	0.1	0.24*	-0.069***	0.000979***	0.00103***	-0.0004***
24 h	0.0625mM +aphids vs. 0.325 mM Pb2++aphids	6.33***	0.231***	-0.15***	2.3***	-8.43***	38.5***	-492.1***	-38.3***	-0.4	0.35***	-0.147***	0.001404***	0.00173***	-0.00073***
24 h	0.1 mM Pb2+ + aphids vs 0.25 mM Pb2++ aphids	1.35	0.022	-0.07*	2.7***	1.59***	-5.7***	-435.9***	22.1***	0.1	-0.11	-0.081***	0.00004	0.00119***	0.00003
24 h	0.1 mM Pb2+ + aphids vs 0.325 mM Pb2++ aphids	4.84***	0.189***	-0.11**	2.3***	-7.17***	-3.1*	-447***	17.5***	-0.4	0	-0.159***	0.000465***	0.00189***	-0.0003*
24 h	0.25 mM Pb2+ + aphids vs 0.325 mM Pb2++ aphids	3.5***	0.167***	-0.04	-0.4	-8.77***	2.5	-11	-4.6	-0.5	0.11	-0.077***	0.000425***	0.0007***	-0.00033**
48 h	control vs. 0.025mM Pb2+	-0.24	-0.028	0.17***	3.1***	-0.05	0.7	-30***	9.5***	0.02	-0.25*	0.004	-0.000153**	-0.00022	-0.00034**
48 h	control vs. 0.05mM Pb2+	-0.52	-0.049	-0.13***	3.7***	-0.13	-2.1	-300.1***	1.8	0.79	0.42***	0.004	-0.000121*	0.0002	-0.00036**
48 h	control vs. 0.0625mM Pb2+	-0.8	-0.044	-0.59***	-5***	0.37	-21.1***	-345.6***	-49.1***	0.83	-0.16	-0.008	-0.000075	-0.00578***	-0.00072***
48 h	control vs. 0.1 mM Pb2+	0.68	0.023	0.14***	6.1***	1.06**	-4.9**	-326.9***	-53.4***	0.79	-1.19***	-2.213***	0.00005	0.00048***	-0.00076***
48 h	control vs. 0.25 mM Pb2+	2.61**	0.141***	0.1**	7.3***	-3.3***	-5.8***	-318.9***	-127.7***	0.3	-0.48***	-0.728***	0.00005	-0.00062***	-0.00036**
48 h	control vs. 0.325 mM Pb2+	6.83***	0.293***	0.13***	19.1***	-0.24	-0.8	4.8	-77.9***	-0.74	-0.02	-0.271***	0.000055	0.00093***	-0.0009***
48 h	0.025mM Pb2+vs 0.05mM Pb2+	-0.28	-0.021	-0.3***	0.6	-0.08	-2.8	-270.1***	-7.7**	0.77	0.67***	0	0.000033	0.00042**	-0.00002
48 h	0.025mM Pb2+vs 0.0625mM Pb2+	-0.57	-0.015	-0.76***	-8.1***	0.43	-21.8***	-315.6***	-58.5***	0.81	0.09	-0.012	0.000078	-0.00556***	-0.00038**
48 h	0.025mM Pb2+vs 0.1 mM Pb2+	0.92	0.051	-0.03	3***	1.11**	-5.6***	-296.9***	-62.9***	0.77	-0.94***	-2.218***	0.000204***	0.0007***	-0.00042***
48 h	0.025mM Pb2+ vs. 0.25 mM Pb2+	2.85**	0.17***	-0.07*	4.2***	-3.25***	-6.5***	-288.9***	-137.2***	0.28	-0.23*	-0.732***	0.000203***	-0.0004**	-0.00002
48 h	0.025mM Pb2+ vs. 0.325 mM Pb2+	7.07***	0.321***	-0.04	16***	-0.19	-1.5	34.8***	-87.4***	-0.76	0.23*	-0.275***	0.000209***	0.00115***	-0.00056***
48 h	0.05mM Pb2+vs 0.0625mM Pb2+	-0.29	0.006	-0.46***	-8.7***	0.5	-19***	-45.5***	-50.8***	0.04	-0.59***	-0.012	0.000045	-0.00598***	-0.00036**
48 h	0.05mM Pb2+ vs 0.1 mM Pb2+	1.2	0.072*	0.27***	2.4***	1.19**	-2.9	-26.8***	-55.2***	0	-1.61***	-2.218***	0.000171**	0.00028*4	-0.0004**
48 h	0.05mM Pb2+ vs. 0.25 mM Pb2+	3.13**	0.191***	0.23***	3.6***	-3.17***	-3.7*	-18.8**	-129.5***	-0.49	-0.91***	-0.732***	0.000171**	-0.00082***	0
48 h	0.05mM Pb2+ vs. 0.325 mM Pb2+	7.35***	0.342***	0.26***	15.4***	-0.11	1.3	304.9***	-79.7***	-1.53**	-0.45***	-0.275***	0.000176**	0.00073***	-0.00054***
48 h	0.0625mM Pb2+vs 0.1 mM Pb2+	1.49	0.066*	0.73***	11.1***	0.68	16.2***	18.7**	-4.4	-0.04	-1.02***	-2.206***	0.000125*	0.00626***	-0.00004
48 h	0.0625mM vs. 0.25 mM Pb2+	3.42***	0.185***	0.69***	12.3***	-3.67***	15.3***	26.7***	-78.6***	-0.53	-0.32**	-0.72***	0.000125*	0.00516***	0.00035**
48 h	0.0625mM vs. 0.325 mM Pb2+	7.64***	0.336***	0.72***	24.1***	-0.61	20.3***	350.4***	-28.9***	-1.57**	0.14	-0.264***	0.000131*	0.00671***	-0.00018
48 h	0.1 mM Pb2+ vs 0.25 mM Pb2+	1.93	0.119***	-0.04	1.2*	-4.36***	-0.8	8	-74.3***	-0.49	0.7***	1.486***	0	-0.0011***	0.00039**
48 h	0.1 mM Pb2+ vs 0.325 mM Pb2+	6.15***	0.27***	-0.01	13***	-1.3***	4.2**	331.7***	-24.5***	-1.53**	1.16***	1.942***	0.000005	0.00044**	-0.00014
48 h	0.25 mM Pb2+vs 0.325 mM Pb2+	4.22***	0.151***	0.03	11.8***	3.06***	5***	323.7***	49.8***	-1.04	0.46***	0.457***	0.000005	0.00155***	-0.00053***
48 h	control +aphids vs. 0.025mM Pb2+ +aphids	-0.07	-0.018	0.473***	10.3***	0.11	3.7*	-281.4***	-17.7***	-0.07	0.05	-0.259***	-0.000057	-0.00029*	0.00029*
48 h	control +aphids vs. 0.05mM Pb2+ +aphids	-0.61	-0.002	0.493***	20.5***	-0.21	11.6***	-498.6***	3.4	-0.07	0.09	-0.01	-0.000064	-0.00067***	0.00023
48 h	control +aphids vs. 0.0625mM Pb2+ +aphids	-0.77	0	0.297***	0.7	0.26	-21.7***	-380.1***	21.4***	-0.39	-0.4***	0.001	0.000028	-0.00001	0.00016
48 h	control +aphids vs. 0.1 mM Pb2+ +aphids	1.32	0.006	0.347***	19.9***	0.22	-5.1***	-223.7***	-6.2*	-0.03	-0.46***	-0.017	-0.000211***	0.00048***	0.00032*
48 h	control +aphids vs. 0.25 mM Pb2+ +aphids	3.57***	0.105***	0.657***	31.8***	-4.64***	-19.6***	-244.1***	-19.4***	-4.1***	-0.54***	-0.144***	-0.00006	-0.00051***	0.00039**
48 h	control +aphids vs. 0.325 mM Pb2+ +aphids	7.14***	0.273***	0.617***	32.3***	-11.24***	0.6	-354.2***	12.1***	-56.12***	-0.21*	-0.091***	-0.000146**	0.00037**	0.0005***
48 h	0.025mM Pb2+ +aphids vs 0.05mM Pb2+ +aphids	-0.54	0.016	0.02	10.2***	-0.32	7.9***	-217.3***	21.1***	0	0.04	0.249***	-0.000007	-0.00038**	-0.00006
48 h	0.025mM Pb2+ +aphids vs 0.0625mM Pb2+ +aphids	-0.7	0.018	-0.177***	-9.6***	0.15	-25.5***	-98.7***	39.1***	-0.32	-0.45***	0.26***	0.000085	0.00027	-0.00012
48 h	0.025mM Pb2+ +aphids vs 0.1 mM Pb2+ +aphids	1.39	0.024	-0.127***	9.6***	0.11	-8.9***	57.7***	11.4***	0.04	-0.5***	0.242***	-0.000155**	0.00077***	0.00003
48 h	0.025mM Pb2+ +aphids vs. 0.25 mM Pb2+ +aphids	3.63***	0.123***	0.183***	21.5***	-4.75***	-23.3***	37.3***	-1.8	-4.03***	-0.59***	0.116***	-0.000003	-0.00022	0.00011

48 h	0.025mM Pb2+ +aphids vs. 0.325 mM Pb2+ +aphids	7.21***	0.291***	0.143***	22***	-11.35***	-3.2*	-72.8***	29.8***	-56.05***	-0.26*	0.168***	-0.00009	0.00065***	0.00021
48 h	0.05mM Pb2+ +aphids vs 0.0625mM Pb2+ +aphids	-0.16	0.001	-0.197***	-19.8***	0.47	-33.4***	118.5***	18***	-0.32	-0.49***	0.011	0.000092	0.00066***	-0.00006
48 h	0.05mM Pb2+ +aphids vs 0.1 mM Pb2+ +aphids	1.93*	0.007	-0.147***	-0.6	0.43	-16.8***	275***	-9.6***	0.04	-0.55***	-0.007	-0.000148**	0.00115***	0.00009
48 h	0.05mM Pb2+ +aphids vs. 0.25 mM Pb2+ +aphids	4.17***	0.107***	0.163***	11.3***	-4.43***	-31.2***	254.5***	-22.8***	-4.03***	-0.63***	-0.133***	0.000004	0.00016	0.00017
48 h	0.05mM Pb2+ +aphids vs. 0.325 mM Pb2+ +aphids	7.75***	0.275***	0.123***	11.8***	-11.03***	-11.1***	144.4***	8.7***	-56.05***	-0.3**	-0.081***	-0.000082	0.00103***	0.00027*
48 h	0.0625mM Pb2+ +aphids vs 0.1 mM Pb2+ +aphids	2.09*	0.006	0.05	19.2***	-0.04	16.6***	156.4***	-27.6***	0.36	-0.06	-0.018	-0.000239***	0.0005***	0.00015
48 h	0.0625mM +aphids vs. 0.25 mM Pb2+ +aphids	4.33***	0.106***	0.36***	31.1***	-4.9***	2.2	136***	-40.8***	-3.71***	-0.14	-0.144***	-0.000088	-0.0005***	0.00023
48 h	0.0625mM +aphids vs. 0.325 mM Pb2+ +aphids	7.91***	0.273***	0.32***	31.6***	-11.5***	22.3***	25.9***	-9.3***	-55.73***	0.19	-0.092***	-0.000174**	0.00038**	0.00033**
48 h	0.1 mM Pb2+ +aphids vs 0.25 mM Pb2+ +aphids	2.24*	0.1***	0.31***	11.9***	-4.86***	-14.4***	-20.4**	-13.2***	-4.07***	-0.08	-0.126***	0.000151**	-0.00099***	0.00008
48 h	0.1 mM Pb2+ +aphids vs 0.325 mM Pb2+ +aphids	5.82***	0.267***	0.27***	12.4***	-11.46***	5.7***	-130.5***	18.4***	-56.09***	0.24*	-0.074***	0.000065	-0.00012	0.00018
48 h	0.25 mM Pb2+ +aphids vs 0.325 mM Pb2+ +aphids	3.58***	0.167***	-0.04	0.5	-6.6***	20.1***	-110.1***	31.5***	-52.02***	0.33**	0.052**	-0.000086	0.00087***	0.0001
48 h	0.025mM Pb2+vs 0.05mM Pb2+ +aphids	-0.65	0.038	0.037	5***	2.13***	6.4***	-618.8***	3.7	-0.3	0.54***	-0.011	0.000133*	0.00027	0.00005
48 h	0.025mM Pb2+vs 0.0625mM Pb2++aphids	-0.81	0.039	-0.16***	-14.8***	2.6***	-26.9***	-500.3***	21.6***	-0.62	0.05	0	0.000225***	0.00093***	-0.00001
48 h	0.025mM Pb2+vs 0.1 mM Pb2++aphids	1.28	0.045	-0.11**	4.4***	2.56***	-10.4***	-343.8***	-6*	-0.26	-0.01	-0.018	-0.000014	0.00142***	0.00014
48 h	0.025mM Pb2+ vs. 0.25 mM Pb2++aphids	3.52***	0.145***	0.2***	16.3***	-2.3***	-24.8***	-364.3***	-19.2***	-4.33***	-0.09	-0.144***	0.000137*	0.00043**	0.00022
48 h	0.025mM Pb2+ vs. 0.325 mM Pb2++aphids	7.1***	0.313***	0.16***	16.8***	-8.9***	-4.6**	-474.3***	12.4***	-56.35***	0.24*	-0.092***	0.000051	0.0013***	0.00032**
48 h	0.05mM Pb2+vs 0.0625mM Pb2+ +aphids	-0.53	0.06*	0.14***	-15.4***	2.68***	-24.2***	-230.1***	29.3***	-1.39**	-0.62***	0	0.000193***	0.00051***	0.00001
48 h	0.05mM Pb2+ vs 0.1 mM Pb2++aphids	1.57	0.066*	0.19***	3.8***	2.64***	-7.6***	-73.7***	1.7	-1.03	-0.68***	-0.018	-0.000047	0.001***	0.00016
48 h	0.05mM Pb2+ vs. 0.25 mM Pb2++aphids	3.81***	0.166***	0.5***	15.7***	-2.22***	-22***	-94.1***	-11.5***	-5.1***	-0.76***	-0.144***	0.000105	0.00001	0.00024
48 h	0.05mM Pb2+ vs. 0.325 mM Pb2++aphids	7.38***	0.334***	0.46***	16.2***	-8.83***	-1.9	-204.2***	20.1***	-57.12***	-0.44***	-0.092***	0.000018	0.00088***	0.00034**
48 h	0.0625mM Pb2+vs 0.1 mM Pb2++aphids	1.85	0.061*	0.65***	12.5***	2.13***	11.4***	-28.2***	52.5***	-1.07*	-0.09	-0.006	-0.000092	0.00698***	0.00052***
48 h	0.0625mM vs. 0.25 mM Pb2++aphids	4.09***	0.161***	0.96***	24.4***	-2.73***	-3*	-48.7***	39.4***	-5.14***	-0.18	-0.132***	0.000059	0.00599***	0.00059***
48 h	0.0625mM vs. 0.325 mM Pb2++aphids	7.67***	0.328***	0.92***	24.9***	-9.33***	17.1***	-158.7***	70.9***	-57.16***	0.15	-0.08***	-0.000027	0.00686***	0.0007***
48 h	0.1 mM Pb2+ vs 0.25 mM Pb2+ +aphids	2.61**	0.094**	0.23***	13.3***	-3.41***	-19.2***	-67.3***	43.7***	-5.1***	0.85***	2.073***	-0.000066	-0.00027	0.00063***
48 h	0.1 mM Pb2+ vs 0.325 mM Pb2++aphids	6.18***	0.261***	0.19***	13.8***	-10.01***	1	-177.4***	75.3***	-57.12***	1.17***	2.126***	-0.000152**	0.0006***	0.00074***
48 h	0.25 mM Pb2+vs 0.325 mM Pb2++aphids	4.25***	0.143***	0.23***	12.6***	-5.66***	1.8	-185.4***	149.5***	-56.63***	0.47***	0.64***	-0.000152**	0.0017***	0.00034**
48 h	0.025mM Pb2++ aphids vs 0.05mM Pb2+ +aphids	-0.54	0.016	0.02	10.2***	-0.32	7.9***	-217.3***	21.1***	0	0.04	0.249***	-0.000007	-0.00038**	-0.00006
48 h	0.025mM Pb2+ + aphids vs 0.0625mM Pb2++aphids	-0.7	0.018	-0.177***	-9.6***	0.15	-25.5***	-98.7***	39.1***	-0.32	-0.45***	0.26***	0.000085	0.00027	-0.00012
48 h	0.025mM Pb2+ + aphids vs 0.1 mM Pb2++aphids	1.39	0.024	-0.127***	9.6***	0.11	-8.9***	57.7***	11.4***	0.04	-0.5***	0.242***	-0.000155**	0.00077***	0.00003
48 h	0.025mM Pb2+ + aphids vs. 0.25 mM Pb2++aphids	3.63***	0.123***	0.183***	21.5***	-4.75***	-23.3***	37.3***	-1.8	-4.03***	-0.59***	0.116***	-0.000003	-0.00022	0.00011
48 h	0.025mM Pb2+ + aphids vs. 0.325 mM Pb2++aphids	7.21***	0.291***	0.143***	22***	-11.35***	-3.2*	-72.8***	29.8***	-56.05***	-0.26*	0.168***	-0.00009	0.00065***	0.00021
48 h	0.05 mM Pb2+ + aphids vs 0.0625mM Pb2++ aphids	-0.16	0.001	-0.197***	-19.8***	0.47	-33.4***	118.5***	18***	-0.32	-0.49***	0.011	0.000092	0.00066***	-0.00006
48 h	0.05mM Pb2+ + aphids vs 0.1 mM Pb2+ + aphids	1.93*	0.007	-0.147***	-0.6	0.43	-16.8***	275***	-9.6***	0.04	-0.55***	-0.007	-0.000148**	0.00115***	0.00009
48 h	0.05mM Pb2+ + aphids vs. 0.25 mM Pb2++ aphids	4.17***	0.107***	0.163***	11.3***	-4.43***	-31.2***	254.5***	-22.8***	-4.03***	-0.63***	-0.133***	0.000004	0.00016	0.00017
48 h	0.05mM Pb2++ aphids vs. 0.325 mM Pb2++ aphids	7.75***	0.275***	0.123***	11.8***	-11.03***	-11.1***	144.4***	8.7***	-56.05***	-0.3**	-0.081***	-0.000082	0.00103***	0.00027*
48 h	0.0625mM Pb2++aphids vs 0.1 mM Pb2++aphids	2.09*	0.006	0.05	19.2***	-0.04	16.6***	156.4***	-27.6***	0.36	-0.06	-0.018	-0.000239***	0.0005***	0.00015
48 h	0.0625mM +aphids vs. 0.25 mM Pb2++aphids	4.33***	0.106***	0.36***	31.1***	-4.9***	2.2	136***	-40.8***	-3.71***	-0.14	-0.144***	-0.000088	-0.0005***	0.00023
48 h	0.0625mM +aphids vs. 0.325 mM Pb2++aphids	7.91***	0.273***	0.32***	31.6***	-11.5***	22.3***	25.9***	-9.3***	-55.73***	0.19	-0.092***	-0.000174**	0.00038**	0.00033**
48 h	0.1 mM Pb2+ + aphids vs 0.25 mM Pb2++ aphids	2.24*	0.1***	0.31***	11.9***	-4.86***	-14.4***	-20.4**	-13.2***	-4.07***	-0.08	-0.126***	0.000151**	-0.00099***	0.00008
48 h	0.1 mM Pb2+ + aphids vs 0.325 mM Pb2++ aphids	5.82***	0.267***	0.27***	12.4***	-11.46***	5.7***	-130.5***	18.4***	-56.09***	0.24*	-0.074***	0.000065	-0.00012	0.00018
48 h	0.25 mM Pb2+ + aphids vs 0.325 mM Pb2++ aphids	3.58***	0.167***	-0.04	0.5	-6.6***	20.1***	-110.1***	31.5***	-52.02***	0.33**	0.052**	-0.000086	0.00087***	0.0001
72 h	control vs. 0.025mM Pb2+	0.31	-0.01	0.29***	-0.2	-1.57***	5.2***	-39.3***	-3.3	-0.44	-0.75***	-0.002	-0.000171**	-0.00031*	0.00002
72 h	control vs. 0.05mM Pb2+	-0.93	-0.021	0.33***	5.7***	-0.18	0	-46.8***	-9.1***	-0.54	-0.6***	0	-0.000157**	-0.00408***	-0.00006
72 h	control vs. 0.0625mM Pb2+	-0.7	-0.025	0.12***	-4.5***	0.26	5.9***	-44.1***	4.2	0.84	-0.3**	0	-0.000146**	-0.00279***	-0.00018

72 h	control vs. 0.1 mM Pb2+	1.16	0.037	0.31***	-2.4***	2.37***	0.3	-12.2	1.1	-1.99***	-0.19	0	-0.000904***	-0.00337***	0.00018
72 h	control vs. 0.25 mM Pb2+	2.77**	0.108***	0.66***	23.7***	0.48	3.2*	-131.8***	-12.7***	0.23	-0.62***	-0.072***	-0.000327***	-0.00229***	0.00007
72 h	control vs. 0.325 mM Pb2+	7.47***	0.278***	0.51***	23***	-4.07***	-3.3*	-47.1***	-45.6***	-5.8***	-1.19***	-0.166***	-0.000297***	-0.00196***	0.00023
72 h	0.025mM Pb2+vs 0.05mM Pb2+	-1.24	-0.011	0.04	5.9***	1.38***	-5.2***	-7.5	-5.8*	-0.11	0.15	0.002	0.000014	-0.00377***	-0.00008
72 h	0.025mM Pb2+vs 0.0625mM Pb2+	-1	-0.015	-0.17***	-4.3***	1.83***	0.6	-4.8	7.5**	1.28*	0.45***	0.002	0.000024	-0.00248***	-0.00021
72 h	0.025mM Pb2+vs 0.1 mM Pb2+	0.85	0.047	0.02	-2.2***	3.94***	-4.9**	27.1***	4.4	-1.55**	0.56***	0.002	-0.000733***	-0.00306***	0.00015
72 h	0.025mM Pb2+ vs. 0.25 mM Pb2+	2.47*	0.118***	0.37***	23.9***	2.05***	-2	-92.5***	-9.4***	0.67	0.13	-0.07***	-0.000156**	-0.00198***	0.00004
72 h	0.025mM Pb2+ vs. 0.325 mM Pb2+	7.16***	0.287***	0.22***	23.2***	-2.5***	-8.5***	-7.9	-42.3***	-5.36***	-0.44***	-0.164***	-0.000126*	-0.00165***	0.00021
72 h	0.05mM Pb2+vs 0.0625mM Pb2+	0.24	-0.004	-0.21***	-10.2***	0.44	5.8***	2.7	13.3***	1.39**	0.3**	0	0.000011	0.00129***	-0.00013
72 h	0.05mM Pb2+ vs 0.1 mM Pb2+	2.09*	0.057	-0.02	-8.1***	2.56***	0.3	34.6***	10.2***	-1.44**	0.41***	0	-0.000747***	0.00071***	0.00024
72 h	0.05mM Pb2+ vs. 0.25 mM Pb2+	3.71***	0.128***	0.33***	18***	0.66	3.2*	-85***	-3.7	0.77	-0.02	-0.072***	-0.00017**	0.00179***	0.00013
72 h	0.05mM Pb2+ vs. 0.325 mM Pb2+	8.4***	0.298***	0.18***	17.3***	-3.89***	-3.3*	-0.4	-36.5***	-5.25***	-0.59***	-0.166***	-0.00014*	0.00212***	0.00029*
72 h	0.0625mM Pb2+vs 0.1 mM Pb2+	1.86	0.062*	0.19***	2.1***	2.11***	-5.6***	31.8***	-3.1	-2.83***	0.11	0	-0.000758***	-0.00058***	0.00036**
72 h	0.0625mM vs. 0.25 mM Pb2+	3.47***	0.133***	0.54***	28.2***	0.22	-2.7	-87.7***	-17***	-0.61	-0.31**	-0.072***	-0.000181***	0.0005***	0.00025*
72 h	0.0625mM vs. 0.325 mM Pb2+	8.16***	0.302***	0.39***	27.5***	-4.33***	-9.1***	-3.1	-49.9***	-6.64***	-0.88***	-0.166***	-0.000151**	0.00083***	0.00042***
72 h	0.1 mM Pb2+ vs 0.25 mM Pb2+	1.61	0.071*	0.35***	26.1***	-1.89***	2.9	-119.6***	-13.8***	2.22***	-0.43***	-0.072***	0.000577***	0.00108***	-0.00011
72 h	0.1 mM Pb2+ vs 0.325 mM Pb2+	6.31***	0.241***	0.2***	25.4***	-6.44***	-3.6*	-34.9***	-46.7***	-3.81***	-1***	-0.166***	0.000607***	0.00141***	0.00006
72 h	0.25 mM Pb2+vs 0.325 mM Pb2+	4.69***	0.17***	-0.15***	-0.7	-4.55***	-6.5***	84.7***	-32.9***	-6.03***	-0.57***	-0.094***	0.00003	0.00033*	0.00017
72 h	control +aphids vs. 0.025mM Pb2+ +aphids	0.62	-0.047	0.14***	6.1***	-0.08	-5.7***	-61.1***	-1.3	-0.25	0.37***	0.001	-0.000115*	-0.0026***	-0.00006
72 h	control +aphids vs. 0.05mM Pb2+ +aphids	-1.15	-0.041	0.16***	6.9***	-0.25	2.4	-18.4**	-13.4***	-1.36*	0.2	0.003	-0.00009	-0.00177***	-0.00009
72 h	control +aphids vs. 0.0625mM Pb2+ +aphids	-1.48	-0.031	-0.05	3.6***	-1.05**	0.7	-20.1**	-24.6***	-1.1*	0.13	0	-0.000144*	-0.00286***	0.00025*
72 h	control +aphids vs. 0.1 mM Pb2+ +aphids	0.35	0.012	-0.03	3.6***	-0.2	-2	0.4	1.4	0.06	0.15	0.002	-0.000758***	-0.00151***	-0.00017
72 h	control +aphids vs. 0.25 mM Pb2+ +aphids	2.58**	0.057	0.41***	29***	0	2.4	-30.5***	1	-0.34	-0.33**	-0.125***	-0.000401***	-0.00015	-0.00037**
72 h	control +aphids vs. 0.325 mM Pb2+ +aphids	6.64***	0.267***	0.507***	29.4***	-2.43***	0.4	-7	-41.1***	-1.96***	-0.62***	-0.153***	-0.000162**	0.00013	-0.00007
72 h	0.025mM Pb2+ +aphids vs 0.05mM Pb2+ +aphids	-1.77	0.005	0.02	0.8	-0.17	8.1***	42.7***	-12.2***	-1.11*	-0.17	0.002	0.000024	0.00083***	-0.00004
72 h	0.025mM Pb2+ +aphids vs 0.0625mM Pb2+ +aphids	-2.1*	0.015	-0.19***	-2.5***	-0.97*	6.5***	41***	-23.3***	-0.84	-0.24*	-0.001	-0.000029	-0.00026	0.00031*
72 h	0.025mM Pb2+ +aphids vs 0.1 mM Pb2+ +aphids	-0.28	0.058*	-0.17***	-2.5***	-0.13	3.7*	61.4***	2.7	0.32	-0.22*	0.001	-0.000643***	0.00109***	-0.00012
72 h	0.025mM Pb2+ +aphids vs. 0.25 mM Pb2+ +aphids	1.96*	0.104***	0.27***	22.9***	0.07	8.1***	30.6***	2.3	-0.09	-0.7***	-0.126***	-0.000287***	0.00245***	-0.00031*
72 h	0.025mM Pb2+ +aphids vs. 0.325 mM Pb2+ +aphids	6.02***	0.313***	0.367***	23.3***	-2.35***	6.1***	54***	-39.8***	-1.71**	-0.99***	-0.154***	-0.000047	0.00273***	-0.00001
72 h	0.05mM Pb2+ +aphids vs 0.0625mM Pb2+ +aphids	-0.33	0.01	-0.21***	-3.3***	-0.8*	-1.7	-1.7	-11.1***	0.26	-0.07	-0.003	-0.000053	-0.00109***	0.00034**
72 h	0.05mM Pb2+ +aphids vs 0.1 mM Pb2+ +aphids	1.5	0.053	-0.19***	-3.3***	0.05	-4.4**	18.8**	14.8***	1.43**	-0.05	-0.001	-0.000667***	0.00026	-0.00008
72 h	0.05mM Pb2+ +aphids vs. 0.25 mM Pb2+ +aphids	3.73***	0.098***	0.25***	22.1***	0.24	0	-12.1	14.4***	1.02	-0.53***	-0.128***	-0.000311***	0.00162***	-0.00028*
72 h	0.05mM Pb2+ +aphids vs. 0.325 mM Pb2+ +aphids	7.79***	0.308***	0.347***	22.5***	-2.18***	-2	11.4	-27.6***	-0.6	-0.82***	-0.156***	-0.000071	0.0019***	0.00002
72 h	0.0625mM Pb2+ +aphids vs 0.1 mM Pb2+ +aphids	1.83	0.043	0.02	0	0.84*	-2.7	20.4**	25.9***	1.16*	0.02	0.002	-0.000614***	0.00135***	-0.00042***
72 h	0.0625mM +aphids vs. 0.25 mM Pb2+ +aphids	4.06***	0.088**	0.46***	25.4***	1.04**	1.7	-10.4	25.6***	0.76	-0.46***	-0.125***	-0.000258***	0.00271***	-0.00062***
72 h	0.0625mM +aphids vs. 0.325 mM Pb2+ +aphids	8.12***	0.298***	0.557***	25.8***	-1.38***	-0.3	13*	-16.5***	-0.87	-0.75***	-0.153***	-0.000018	0.00299***	-0.00032*
72 h	0.1 mM Pb2+ +aphids vs 0.25 mM Pb2+ +aphids	2.23*	0.045	0.44***	25.4***	0.2	4.4**	-30.8***	-0.4	-0.41	-0.48***	-0.127***	0.000356***	0.00136***	-0.0002
72 h	0.1 mM Pb2+ +aphids vs 0.325 mM Pb2+ +aphids	6.3***	0.255***	0.537***	25.8***	-2.22***	2.4	-7.4	-42.5***	-2.03***	-0.77***	-0.155***	0.000596***	0.00164***	0.00011
72 h	0.25 mM Pb2+ +aphids vs 0.325 mM Pb2+ +aphids	4.06***	0.21***	0.097**	0.4	-2.42***	-2	23.4***	-42.1***	-1.62**	-0.29**	-0.028	0.00024***	0.00028*	0.0003*
72 h	0.025mM Pb2+vs 0.05mM Pb2+ +aphids	-1.09	0.004	0.09*	1.2*	5.45***	0.6	-16.6**	-6.1*	-0.32	0.51***	0.002	0.000101	-0.00358***	-0.00005
72 h	0.025mM Pb2+vs 0.0625mM Pb2++aphids	-1.42	0.014	-0.12***	-2.1***	4.65***	-1.1	-18.3**	-17.2***	-0.05	0.43***	-0.001	0.000048	-0.00467***	0.00029*
72 h	0.025mM Pb2+vs 0.1 mM Pb2++aphids	0.4	0.057	-0.1**	-2.1***	5.5***	-3.8*	2.1	8.7***	1.11*	0.46***	0.001	-0.000566***	-0.00332***	-0.00013
72 h	0.025mM Pb2+ vs. 0.25 mM Pb2++aphids	2.64**	0.102***	0.34***	23.3***	5.7***	0.6	-28.7***	8.3***	0.7	-0.02	-0.126***	-0.00021***	-0.00196***	-0.00033**
72 h	0.025mM Pb2+ vs. 0.325 mM Pb2++aphids	6.7***	0.312***	0.437***	23.7***	3.27***	-1.4	-5.3	-33.7***	-0.92	-0.32**	-0.154***	0.00003	-0.00168***	-0.00003

72 h	0.05mM Pb2+vs 0.0625mM Pb2+ +aphids	-0.18	0.025	-0.16***	-8***	3.27***	4.1**	-10.8	-11.4***	0.05	0.28**	-0.003	0.000034	-0.0009***	0.00037**
72 h	0.05mM Pb2+ vs 0.1 mM Pb2++aphids	1.64	0.068*	-0.14***	-8***	4.11***	1.4	9.6	14.5***	1.22*	0.3**	-0.001	-0.00058***	0.00045**	-0.00005
72 h	0.05mM Pb2+ vs. 0.25 mM Pb2++aphids	3.88***	0.113***	0.3***	17.4***	4.31***	5.8***	-21.2***	14.1***	0.81	-0.18	-0.128***	-0.000224***	0.00181***	-0.00025*
72 h	0.05mM Pb2+ vs. 0.325 mM Pb2++aphids	7.94***	0.323***	0.397***	17.8***	1.89***	3.8*	2.2	-28***	-0.81	-0.47***	-0.156***	0.000016	0.00209***	0.00006
72 h	0.0625mM Pb2+vs 0.1 mM Pb2++aphids	1.41	0.072*	0.07*	2.2***	3.67***	-4.4**	6.9	1.2	-0.17	0.01	-0.001	-0.000591***	-0.00084***	0.00007
72 h	0.0625mM vs. 0.25 mM Pb2++aphids	3.64***	0.117***	0.51***	27.6***	3.87***	-0.1	-24***	0.8	-0.58	-0.47***	-0.128***	-0.000235***	0.00052***	-0.00012
72 h	0.0625mM vs. 0.325 mM Pb2++aphids	7.7***	0.327***	0.607***	28***	1.45***	-2.1	-0.5	-41.3***	-2.2***	-0.77***	-0.156***	0.000005	0.0008***	0.00018
72 h	0.1 mM Pb2+ vs 0.25 mM Pb2+ +aphids	1.79	0.056	0.32***	25.5***	1.76***	5.5***	-55.8***	3.9	2.25***	-0.58***	-0.128***	0.000523***	0.0011***	-0.00048***
72 h	0.1 mM Pb2+ vs 0.325 mM Pb2++aphids	5.85***	0.266***	0.417***	25.9***	-0.67	3.5*	-32.4***	-38.1***	0.63	-0.88***	-0.156***	0.000763***	0.00138***	-0.00018
72 h	0.25 mM Pb2+vs 0.325 mM Pb2++aphids	4.23***	0.195***	0.067	-0.2	1.23**	0.6	87.2***	-24.3***	-1.59**	-0.45***	-0.084***	0.000186***	0.0003*	-0.00007
72 h	0.025mM Pb2++ aphids vs 0.05mM Pb2+ +aphids	-1.77	0.005	0.02	0.8	-0.17	8.1***	42.7***	-12.2***	-1.11*	-0.17	0.002	0.000024	0.00083***	-0.00004
72 h	0.025mM Pb2+ + aphids vs 0.0625mM Pb2++aphids	-2.1*	0.015	-0.19***	-2.5***	-0.97*	6.5***	41***	-23.3***	-0.84	-0.24*	-0.001	-0.000029	-0.00026	0.00031*
72 h	0.025mM Pb2+ + aphids vs 0.1 mM Pb2++aphids	-0.28	0.058*	-0.17***	-2.5***	-0.13	3.7*	61.4***	2.7	0.32	-0.22*	0.001	-0.000643***	0.00109***	-0.00012
72 h	0.025mM Pb2+ + aphids vs. 0.25 mM Pb2++aphids	1.96*	0.104***	0.27***	22.9***	0.07	8.1***	30.6***	2.3	-0.09	-0.7***	-0.126***	-0.000287***	0.00245***	-0.00031*
72 h	0.025mM Pb2+ + aphids vs. 0.325 mM Pb2++aphids	6.02***	0.313***	0.367***	23.3***	-2.35***	6.1***	54***	-39.8***	-1.71**	-0.99***	-0.154***	-0.000047	0.00273***	-0.00001
72 h	0.05 mM Pb2+ + aphids vs 0.0625mM Pb2++ aphids	-0.33	0.01	-0.21***	-3.3***	-0.8*	-1.7	-1.7	-11.1***	0.26	-0.07	-0.003	-0.000053	-0.00109***	0.00034**
72 h	0.05mM Pb2+ + aphids vs 0.1 mM Pb2+ + aphids	1.5	0.053	-0.19***	-3.3***	0.05	-4.4**	18.8**	14.8***	1.43**	-0.05	-0.001	-0.000667***	0.00026	-0.00008
72 h	0.05mM Pb2+ + aphids vs. 0.25 mM Pb2++ aphids	3.73***	0.098***	0.25***	22.1***	0.24	0	-12.1	14.4***	1.02	-0.53***	-0.128***	-0.000311***	0.00162***	-0.00028*
72 h	0.05mM Pb2++ aphids vs. 0.325 mM Pb2++ aphids	7.79***	0.308***	0.347***	22.5***	-2.18***	-2	11.4	-27.6***	-0.6	-0.82***	-0.156***	-0.000071	0.0019***	0.00002
72 h	0.0625mM Pb2++aphids vs 0.1 mM Pb2++aphids	1.83	0.043	0.02	0	0.84*	-2.7	20.4**	25.9***	1.16*	0.02	0.002	-0.000614***	0.00135***	-0.00042***
72 h	0.0625mM +aphids vs. 0.25 mM Pb2++aphids	4.06***	0.088**	0.46***	25.4***	1.04**	1.7	-10.4	25.6***	0.76	-0.46***	-0.125***	-0.000258***	0.00271***	-0.00062***
72 h	0.0625mM +aphids vs. 0.325 mM Pb2++aphids	8.12***	0.298***	0.557***	25.8***	-1.38***	-0.3	13*	-16.5***	-0.87	-0.75***	-0.153***	-0.000018	0.00299***	-0.00032*
72 h	0.1 mM Pb2+ + aphids vs 0.25 mM Pb2++ aphids	2.23*	0.045	0.44***	25.4***	0.2	4.4**	-30.8***	-0.4	-0.41	-0.48***	-0.127***	0.000356***	0.00136***	-0.0002
72 h	0.1 mM Pb2+ + aphids vs 0.325 mM Pb2++ aphids	6.3***	0.255***	0.537***	25.8***	-2.22***	2.4	-7.4	-42.5***	-2.03***	-0.77***	-0.155***	0.000596***	0.00164***	0.00011
72 h	0.25 mM Pb2+ + aphids vs 0.325 mM Pb2++ aphids	4.06***	0.21***	0.097**	0.4	-2.42***	-2	23.4***	-42.1***	-1.62**	-0.29**	-0.028	0.00024***	0.00028*	0.0003*

Table S2. Comparisons between particular levels of analyzed factor in the leaves of pea seedlings in different times (independently) using the two-sample *t*-test for equal means for all observed traits.

Contrasts		Length of epicotyl	Fresh weight	Semiquinone radical	Mn^{2+}	ABA	IAA	JA	SA	Pisatin	Glucose	Sucrose	Acid invertase	Alkaline/neutral invertase	Total soluble sugar
0 h	control vs. 0.025mM Pb2+	-0.48	-0.04	-0.51***	-2.7***	25*	1.7	-107***	2.4	-3.4	-0.51	-0.089	-0.00006	0.000031	17080***
0 h	control vs. 0.05mM Pb2+	-0.12	-0.003	-0.21***	-3.8***	4	0.9	-201***	0.4	-10.4***	-0.7*	0	-0.00013	-0.000025	-816
0 h	control vs. 0.0625mM Pb2+	-0.07	-0.037	-0.32	-6.2***	5	-2.4	-182***	4.9	-4.1*	0.02	0.012	-0.00003	-0.000079*	-14953***
0 h	control vs. 0.1 mM Pb2+	0.48	-0.01	-0.6***	-1.8***	0	1	-95***	1.4	-4*	-1.07***	-0.083	-0.00091***	-0.000234***	-10141*
0 h	control vs. 0.25 mM Pb2+	0.29	0.03	0.02	-2.2***	-56***	-2.2	-64***	2.8	-1.7	-1.82***	-0.409***	-0.00108***	-0.000116***	-22666***
0 h	control vs. 0.325 mM Pb2+	0.24	0.064	-0.86***	0.7*	-140***	-2.3	-75***	1.5	-5.2**	-1.93***	-0.378***	-0.00101***	-0.00024***	-46535***
0 h	0.025mM Pb2+vs 0.05mM Pb2+	0.36	0.037	0.3***	-1.1***	-21	-0.8	-95***	-2	-7.1***	-0.2	0.089	-0.00006	-0.000057	-17897***
0 h	0.025mM Pb2+vs 0.0625mM Pb2+	0.41	0.003	0.43***	-3.5***	-20	-4*	-76***	2.5	-0.7	0.52	0.101	0.00003	-0.00011***	-32033***
0 h	0.025mM Pb2+vs 0.1 mM Pb2+	0.96	0.03	-0.09*	0.9**	-25*	-0.7	11	-0.9	-0.7	-2.28	0.006	-0.00085***	-0.000265***	-27221***
0 h	0.025mM Pb2+ vs. 0.25 mM Pb2+	0.77	0.069	0.53***	0.5	-81***	-3.9*	43*	0.4	1.7	-1.31***	-0.32***	-0.00102***	-0.000147***	-39746***
0 h	0.025mM Pb2+ vs. 0.325 mM Pb2+	0.72	0.104	-0.35***	3.4***	-165***	-4*	32	-0.8	-1.9	-1.42***	-0.29***	-0.00095***	-0.000271***	-63616***
0 h	0.05mM Pb2+vs 0.0625mM Pb2+	0.05	-0.034	0.13***	-2.4***	1	-3.2	19	4.6	6.3***	0.72*	0.012	0.00009	-0.000053	-14137**
0 h	0.05mM Pb2+ vs 0.1 mM Pb2+	0.6	-0.007	-0.39***	2***	-5	0.1	106***	1.1	6.4***	-0.37	-0.083	-0.00079***	-0.000209***	-9324*
0 h	0.05mM Pb2+ vs. 0.25 mM Pb2+	0.4	0.033	0.23***	1.6***	-60***	-3.1	137***	2.4	8.7***	-1.12***	-0.409***	-0.00095***	-0.00009**	-21850***
0 h	0.05mM Pb2+ vs. 0.325 mM Pb2+	0.36	0.067	-0.65***	4.5***	-144***	-3.2	127***	1.2	5.2**	-1.22***	-0.379***	-0.00088***	-0.000214***	-45719***
0 h	0.0625mM Pb2+vs 0.1 mM Pb2+	0.55	0.027	-0.52***	4.4***	-5	3.4	87***	-3.5	0.1	-1.09***	-0.095	-0.00088***	-0.000155***	4813
0 h	0.0625mM vs. 0.25 mM Pb2+	0.35	0.067	0.1*	4***	-61***	0.2	118***	-2.1	2.4	-1.83***	-0.421***	-0.00104***	-0.000037	-7713
0 h	0.0625mM vs. 0.325 mM Pb2+	0.31	0.101	-0.78***	6.9***	-145***	0.1	108***	-3.4	-1.1	-1.94***	-0.391***	-0.00097***	-0.000161***	-31582***
0 h	0.1 mM Pb2+ vs 0.25 mM Pb2+	-0.2	0.039	0.62***	-0.4	-55***	-3.2	31	1.4	2.3	-0.75**	-0.326***	-0.00017	0.000118***	-12525**
0 h	0.1 mM Pb2+ vs 0.325 mM Pb2+	-0.24	0.074	-0.26***	2.5***	-139***	-3.3	21	0.1	-1.2	-0.86**	-0.296***	-0.0001	-0.000006	-36395***
0 h	0.25 mM Pb2+vs 0.325 mM Pb2+	-0.04	0.035	-0.88***	2.9***	-84***	-0.1	-10	-1.2	-3.5	-0.11	0.03	0.00007	-0.000124***	-23869***
24 h	control vs. 0.025mM Pb2+	-0.25	-0.051	-0.46***	-0.9**	-8	3.5	-133***	-30.3***	-1	0.13	0.068	-0.00056***	-0.000096**	3631
24 h	control vs. 0.05mM Pb2+	-0.49	-0.078	0.003	1.2***	41***	-0.2	-3	7*	-12***	-0.54	-0.101	-0.00055***	-0.000195***	10356*
24 h	control vs. 0.0625mM Pb2+	-0.16	-0.022	-0.007	0.7*	36**	2.8	-203***	-4	-12***	-0.27	0.019	-0.00069***	-0.000066*	15856***
24 h	control vs. 0.1 mM Pb2+	-0.16	0.019	0.183***	2.03***	41***	-11.4***	-1194***	5.7	-18***	-1.51***	-0.621***	-0.00064***	-0.000076*	4684
24 h	control vs. 0.25 mM Pb2+	0.23	0.048	0.913***	2.03***	33**	-21.9***	-1580***	5.6	-21***	-2.04***	0.007	-0.00064***	-0.000252***	-11580**
24 h	control vs. 0.325 mM Pb2+	1.1	0.114	0.673***	4.63***	-17	-4.3*	-1519***	-10.4***	-24***	-2.68***	-0.593***	-0.00102***	-0.000236***	-22279***
24 h	0.025mM Pb2+vs 0.05mM Pb2+	-0.24	-0.027	0.463***	2.1***	49***	-3.7*	130***	37.2***	-11***	-0.68*	-0.169**	0.00001	-0.0001**	6725
24 h	0.025mM Pb2+vs 0.0625mM Pb2+	0.09	0.029	0.453***	1.6***	43***	-0.7	-70***	26.3***	-11***	-0.4	-0.049	-0.00013	0.00003	12225**
24 h	0.025mM Pb2+vs 0.1 mM Pb2+	0.1	0.07	0.643***	2.93***	48***	-14.9***	-1060***	36***	-17***	-1.64***	-0.689***	-0.00008	0.00002	1053
24 h	0.025mM Pb2+ vs. 0.25 mM Pb2+	0.48	0.099	1.373***	2.93***	41***	-25.4***	-1446***	35.8***	-20***	-2.18***	-0.061	-0.00008	-0.000156***	-15211***
24 h	0.025mM Pb2+ vs. 0.325 mM Pb2+	1.35	0.165*	1.133***	5.53***	-9	-7.8***	-1386***	19.9***	-23***	-2.82***	-0.661***	-0.00046***	-0.00014***	-25910***
24 h	0.05mM Pb2+vs 0.0625mM Pb2+	0.32	0.057	-0.01	-0.5	-5	3	-200***	-10.9***	0	0.28	0.121*	-0.00014	0.000129***	5500
24 h	0.05mM Pb2+ vs 0.1 mM Pb2+	0.33	0.097	0.18***	0.83**	0	-11.2***	-1191***	-1.2	-6**	-0.96***	-0.519***	-0.00009	0.00012***	-5672
24 h	0.05mM Pb2+ vs. 0.25 mM Pb2+	0.72	0.126	0.91***	0.83**	-8	-21.7***	-1577***	-1.4	-9***	-1.5***	0.108	-0.00009	-0.000056	-21936***
24 h	0.05mM Pb2+ vs. 0.325 mM Pb2+	1.58*	0.192**	0.67***	3.43***	-58***	-4.1*	-1516***	-17.4***	-12***	-2.14***	-0.492***	-0.00047***	-0.000041	-32635***
24 h	0.0625mM Pb2+vs 0.1 mM Pb2+	0.01	0.04	0.19***	1.33***	5	-14.2***	-990***	9.7***	-6**	-1.24***	-0.64***	0.00005	-0.00001	-11172*
24 h	0.0625mM vs. 0.25 mM Pb2+	0.39	0.07	0.92***	1.33***	-2	-24.7***	-1376***	9.6**	-9***	-1.77***	-0.013	0.00005	-0.000186***	-27436***
24 h	0.0625mM vs. 0.325 mM Pb2+	1.26	0.135*	0.68***	3.93***	-52***	-7.1***	-1316***	-6.4*	-12***	-2.42***	-0.612***	-0.00033**	-0.00017***	-38135***

24 h	0.1 mM Pb2+ vs 0.25 mM Pb2+	0.38	0.029	0.73***	0	-7	-10.5***	-386***	-0.2	-3	-0.54	0.627***	0	-0.000176***	-16264***
24 h	0.1 mM Pb2+ vs 0.325 mM Pb2+	1.25	0.095	0.49***	2.6***	-57***	7.1***	-325***	-16.1***	-6**	-1.18***	0.028	-0.00038**	-0.00016***	-26963***
24 h	0.25 mM Pb2+vs 0.325 mM Pb2+	0.87	0.066	-0.24***	2.6***	-50***	17.6***	61***	-16***	-3	-0.64*	-0.6***	-0.00038**	0.000016	-10699*
24 h	control +aphids vs. 0.025mM Pb2+ +aphids	0.1	0.003	0.1*	-3***	-89***	-0.3	185***	86.1***	-13***	0.4	-0.038	-0.00037**	0.000005	-2900
24 h	control +aphids vs. 0.05mM Pb2+ +aphids	-0.77	-0.049	0.19***	1.3***	-47***	-6.3***	-137***	29.5***	-8***	0.13	-0.068	-0.00045***	-0.000088**	-36481***
24 h	control +aphids vs. 0.0625mM Pb2+ +aphids	-0.19	-0.015	0.32***	0.5	-66***	1.3	67***	79.1***	3	-0.32	-0.096	-0.00031*	-0.000218***	-17897***
24 h	control +aphids vs. 0.1 mM Pb2+ +aphids	0.06	0.065	0.48***	-3.1***	-22	-7.9***	-245***	75.2***	-22***	0	-0.21***	-0.00074***	-0.000435***	-2600
24 h	control +aphids vs. 0.25 mM Pb2+ +aphids	0.8	0.076	0.7***	-6.7***	-19	-4.5*	-379***	128.1***	-23***	-1.71***	-0.382***	-0.00071***	-0.000478***	-15297***
24 h	control +aphids vs. 0.325 mM Pb2+ +aphids	1.7*	0.142*	1.05***	-5.6***	-28*	-21.6***	-1173***	13.5***	-24***	-2.89***	-0.731***	-0.00104***	-0.000247***	-45053***
24 h	0.025mM Pb2+ +aphids vs 0.05mM Pb2+ +aphids	-0.86	-0.052	0.09*	4.3***	42***	-6.1***	-322***	-56.6***	5*	-0.26	-0.03	-0.00008	-0.000093**	-33580***
24 h	0.025mM Pb2+ +aphids vs 0.0625mM Pb2+ +aphids	-0.29	-0.019	0.22***	3.5***	24*	1.6	-118***	-7.1*	16***	-0.71*	-0.057	0.00007	-0.000223***	-14996***
24 h	0.025mM Pb2+ +aphids vs 0.1 mM Pb2+ +aphids	-0.03	0.062	0.38***	-0.1	67***	-7.7***	-430***	-10.9***	-9***	-0.39	-0.171**	-0.00036**	-0.00044***	301
24 h	0.025mM Pb2+ +aphids vs. 0.25 mM Pb2+ +aphids	0.7	0.073	0.6***	-3.7***	70***	-4.3*	-564***	42***	-10***	-2.1***	-0.343***	-0.00033**	-0.000483***	-12397**
24 h	0.025mM Pb2+ +aphids vs. 0.325 mM Pb2+ +aphids	1.6*	0.139*	0.95***	-2.6***	61***	-21.4***	-1358***	-72.6***	-11***	-3.29***	-0.692***	-0.00066***	-0.000251***	-42153***
24 h	0.05mM Pb2+ +aphids vs 0.0625mM Pb2+ +aphids	0.57	0.034	0.13***	-0.8**	-18	7.7***	204***	49.5***	11***	-0.45	-0.028	0.00014	-0.00013***	18584***
24 h	0.05mM Pb2+ +aphids vs 0.1 mM Pb2+ +aphids	0.83	0.114	0.29***	-4.4***	26*	-1.6	-108***	45.7***	-14***	-0.13	-0.142*	-0.00029*	-0.000347***	33881***
24 h	0.05mM Pb2+ +aphids vs. 0.25 mM Pb2+ +aphids	1.56*	0.125	0.51***	-8***	28*	1.8	-242***	98.6***	-15***	-1.84***	-0.314***	-0.00026*	-0.00039***	21184***
24 h	0.05mM Pb2+ +aphids vs. 0.325 mM Pb2+ +aphids	2.46**	0.191**	0.86***	-6.9***	19	-15.3***	-1035***	-16***	-16***	-3.03***	-0.663***	-0.00059***	-0.000158***	-8572
24 h	0.0625mM Pb2+ +aphids vs 0.1 mM Pb2+ +aphids	0.26	0.08	0.16***	-3.6***	44***	-9.3***	-312***	-3.8	-25***	0.32	-0.114	-0.00043***	-0.000218***	15297***
24 h	0.0625mM +aphids vs. 0.25 mM Pb2+ +aphids	0.99	0.091	0.38***	-7.2***	46***	-5.9***	-446***	49.1***	-26***	-1.39***	-0.286***	-0.0004***	-0.000261***	2600
24 h	0.0625mM +aphids vs. 0.325 mM Pb2+ +aphids	1.89*	0.157*	0.73***	-6.1***	37***	-23***	-1240***	-65.6***	-27***	-2.58***	-0.635***	-0.00073***	-0.000029	-27156***
24 h	0.1 mM Pb2+ +aphids vs 0.25 mM Pb2+ +aphids	0.73	0.011	0.22***	-3.6***	2	3.4	-134***	52.9***	-1	-1.71***	-0.172**	0.00003	-0.000043	-12697**
24 h	0.1 mM Pb2+ +aphids vs 0.325 mM Pb2+ +aphids	1.63*	0.077	0.57***	-2.5***	-7	-13.7***	-927***	-61.7***	-2	-2.9***	-0.521***	-0.0003*	0.000189***	-42453***
24 h	0.25 mM Pb2+ +aphids vs 0.325 mM Pb2+ +aphids	0.9	0.066	0.35***	1.1***	-9	-17.1***	-794***	-114.6***	-1	-1.19***	-0.349***	-0.00033**	0.000232***	-29756***
24 h	0.025mM Pb2+vs 0.05mM Pb2+ +aphids	-0.4	0.015	0.563***	1.73***	21	-8.2***	-149***	-126.7***	-25***	-0.21	-0.053	0.00044***	0.000092**	-19873***
24 h	0.025mM Pb2+vs 0.0625mM Pb2++aphids	0.17	0.049	0.693***	0.93**	3	-0.5	55***	-77.2***	-14***	-0.66*	-0.081	0.00058***	-0.000037	-1289
24 h	0.025mM Pb2+vs 0.1 mM Pb2++aphids	0.43	0.129*	0.853***	-2.67***	47***	-9.8***	-257***	-81***	-39***	-0.34	-0.195**	0.00015	-0.000255***	14008**
24 h	0.025mM Pb2+ vs. 0.25 mM Pb2++aphids	1.16	0.14*	1.073***	-6.27***	49***	-6.4***	-391***	-28.1***	-40***	-2.05***	-0.367***	0.00018	-0.000298***	1311
24 h	0.025mM Pb2+ vs. 0.325 mM Pb2++aphids	2.06**	0.206**	1.423***	-5.17***	40***	-23.5***	-1184***	-142.8***	-41***	-3.24***	-0.716***	-0.00015	-0.000066*	-28446***
24 h	0.05mM Pb2+vs 0.0625mM Pb2+ +aphids	0.41	0.076	0.23***	-1.17***	-45***	3.1	-75***	-114.5***	-3	0.02	0.088	0.00057***	0.000062	-8014
24 h	0.05mM Pb2+ vs 0.1 mM Pb2++aphids	0.67	0.157*	0.39***	-4.77***	-2	-6.1***	-387***	-118.3***	-28***	0.34	-0.026	0.00014	-0.000155***	7283
24 h	0.05mM Pb2+ vs. 0.25 mM Pb2++aphids	1.4	0.167*	0.61***	-8.37***	1	-2.7	-521***	-65.4***	-29***	-1.37***	-0.197***	0.00017	-0.000198***	-5414
24 h	0.05mM Pb2+ vs. 0.325 mM Pb2++aphids	2.3**	0.234***	0.96***	-7.27***	-8	-19.8***	-1315***	-180***	-30***	-2.56***	-0.546***	-0.00016	0.000034	-35170***
24 h	0.0625mM Pb2+vs 0.1 mM Pb2++aphids	0.34	0.1	0.4***	-4.27***	4	-9.1***	-187***	-107.3***	-28***	0.06	-0.146*	0.00028*	-0.000285***	1783
24 h	0.0625mM vs. 0.25 mM Pb2++aphids	1.07	0.111	0.62***	-7.87***	6	-5.8**	-321***	-54.4***	-29***	-1.65***	-0.318***	0.00031*	-0.000328***	-10914*
24 h	0.0625mM vs. 0.325 mM Pb2++aphids	1.97*	0.177**	0.97***	-6.77***	-3	-22.8***	-1115***	-169.1***	-30***	-2.84***	-0.667***	-0.00002	-0.000096**	-40670***
24 h	0.1 mM Pb2+ vs 0.25 mM Pb2+ +aphids	1.07	0.07	0.43***	-9.2***	1	8.4***	670***	-64.2***	-23***	-0.41	0.322***	0.00026*	-0.000318***	258
24 h	0.1 mM Pb2+ vs 0.325 mM Pb2++aphids	1.97*	0.137*	0.78***	-8.1***	-8	-8.6***	-124***	-178.8***	-24***	-1.6***	-0.027	-0.00007	-0.000086**	-29498***
24 h	0.25 mM Pb2+vs 0.325 mM Pb2++aphids	1.58*	0.107	0.05	-8.1***	-1	1.9	262***	-178.6***	-21***	-1.06***	-0.654***	-0.00007	0.00009**	-13234**
24 h	0.025mM Pb2++ aphids vs 0.05mM Pb2+ +aphids	-0.86	-0.052	0.09*	4.3***	42***	-6.1***	-322***	-56.6***	5*	-0.26	-0.03	-0.00008	-0.000093**	-33580***
24 h	0.025mM Pb2+ + aphids vs 0.0625mM Pb2++aphids	-0.29	-0.019	0.22***	3.5***	24*	1.6	-118***	-7.1*	16***	-0.71*	-0.057	0.00007	-0.000223***	-14996***
24 h	0.025mM Pb2+ + aphids vs 0.1 mM Pb2++aphids	-0.03	0.062	0.38***	-0.1	67***	-7.7***	-430***	-10.9***	-9***	-0.39	-0.171**	-0.00036**	-0.00044***	301
24 h	0.025mM Pb2+ + aphids vs. 0.25 mM Pb2++aphids	0.7	0.073	0.6***	-3.7***	70***	-4.3*	-564***	42***	-10***	-2.1***	-0.343***	-0.00033**	-0.000483***	-12397**
24 h	0.025mM Pb2+ + aphids vs. 0.325 mM Pb2++aphids	1.6*	0.139*	0.95***	-2.6***	61***	-21.4***	-1358***	-72.6***	-11***	-3.29***	-0.692***	-0.00066***	-0.000251***	-42153***

24 h	0.05 mM Pb2+ + aphids vs 0.0625mM Pb2++ aphids	0.57	0.034	0.13***	-0.8**	-18	7.7***	204***	49.5***	11***	-0.45	-0.028	0.00014	-0.00013***	18584***
24 h	0.05mM Pb2+ + aphids vs 0.1 mM Pb2+ + aphids	0.83	0.114	0.29***	-4.4***	26*	-1.6	-108***	45.7***	-14***	-0.13	-0.142*	-0.00029*	-0.000347***	33881***
24 h	0.05mM Pb2+ + aphids vs. 0.25 mM Pb2++ aphids	1.56*	0.125	0.51***	-8***	28*	1.8	-242***	98.6***	-15***	-1.84***	-0.314***	-0.00026*	-0.00039***	21184***
24 h	0.05mM Pb2++ aphids vs. 0.325 mM Pb2++ aphids	2.46**	0.191**	0.86***	-6.9***	19	-15.3***	-1035***	-16***	-16***	-3.03***	-0.663***	-0.00059***	-0.000158***	-8572
24 h	0.0625mM Pb2++aphids vs 0.1 mM Pb2++aphids	0.26	0.08	0.16***	-3.6***	44***	-9.3***	-312***	-3.8	-25***	0.32	-0.114	-0.00043***	-0.000218***	15297***
24 h	0.0625mM +aphids vs. 0.25 mM Pb2++aphids	0.99	0.091	0.38***	-7.2***	46***	-5.9***	-446***	49.1***	-26***	-1.39***	-0.286***	-0.0004***	-0.000261***	2600
24 h	0.0625mM +aphids vs. 0.325 mM Pb2++aphids	1.89*	0.157*	0.73***	-6.1***	37***	-23***	-1240***	-65.6***	-27***	-2.58***	-0.635***	-0.00073***	-0.000029	-27156***
24 h	0.1 mM Pb2+ + aphids vs 0.25 mM Pb2++ aphids	0.73	0.011	0.22***	-3.6***	2	3.4	-134***	52.9***	-1	-1.71***	-0.172**	0.00003	-0.000043	-12697**
24 h	0.1 mM Pb2+ + aphids vs 0.325 mM Pb2++ aphids	1.63*	0.077	0.57***	-2.5***	-7	-13.7***	-927***	-61.7***	-2	-2.9***	-0.521***	-0.0003*	0.000189***	-42453***
24 h	0.25 mM Pb2+ + aphids vs 0.325 mM Pb2++ aphids	0.9	0.066	0.35***	1.1***	-9	-17.1***	-794***	-114.6***	-1	-1.19***	-0.349***	-0.00033**	0.000232***	-29756***
48 h	control vs. 0.025mM Pb2+	-0.41	-0.04	0.34***	-0.2	-81***	-1.1	41*	-7.6*	0	0.6*	0.094	-0.00034**	-0.000224***	-12719**
48 h	control vs. 0.05mM Pb2+	-0.87	-0.079	0.23***	-1.9***	-17	4.7**	-138***	-4.1	-1	1.42***	0.3***	-0.00036**	-0.000352***	-12568**
48 h	control vs. 0.0625mM Pb2+	-0.93	-0.034	0.56***	0.97***	79***	-11.3***	-364***	-7.1*	-3.9*	1.11***	0.211***	-0.00072***	-0.000338***	-7606
48 h	control vs. 0.1 mM Pb2+	-0.06	0.047	0.16***	0.87**	111***	7.6***	-1602***	-3.4	-11***	-2.06***	-0.831***	-0.00076***	-0.000213***	-10484*
48 h	control vs. 0.25 mM Pb2+	0.15	0.109	0.8***	2.37***	101***	5.3**	-1013***	-5.8*	-10***	-2.42***	-0.056	-0.00036**	-0.000096**	-10850*
48 h	control vs. 0.325 mM Pb2+	1.59*	0.228***	0.18***	-0.23	-1	-5**	-1147***	-14.6***	-11***	-2.27***	0.091	-0.0009***	0.000031	-43141***
48 h	0.025mM Pb2+vs 0.05mM Pb2+	-0.46	-0.04	-0.11**	-1.7***	63***	5.8**	-179***	3.5	-1	0.82**	0.205***	-0.00002	-0.000128***	150
48 h	0.025mM Pb2+vs 0.0625mM Pb2+	-0.51	0.006	0.22***	1.17***	159***	-10.2***	-405***	0.5	-3.9*	0.51	0.117	-0.00038**	-0.000114***	5113
48 h	0.025mM Pb2+vs 0.1 mM Pb2+	0.36	0.087	-0.18***	1.07***	191***	8.7***	-1643***	4.2	-11***	-2.66***	-0.925***	-0.00042***	0.000011	2234
48 h	0.025mM Pb2+ vs. 0.25 mM Pb2+	0.56	0.149*	0.46***	2.57***	182***	6.4***	-1055***	1.8	-10***	-3.02***	-0.15*	-0.00002	0.000128***	1869
48 h	0.025mM Pb2+ vs. 0.325 mM Pb2+	2*	0.268***	-0.16***	-0.03	79***	-3.9*	-1189***	-7*	-11***	-2.87***	-0.003	-0.00056***	0.000255***	-30422***
48 h	0.05mM Pb2+vs 0.0625mM Pb2+	-0.06	0.045	0.33***	2.87***	96***	-16***	-226***	-3	-2.9	-0.32	-0.089	-0.00036**	0.000014	4963
48 h	0.05mM Pb2+ vs 0.1 mM Pb2+	0.81	0.126	-0.07	2.77***	128***	2.9	-1464***	0.7	-10***	-3.48***	-1.131***	-0.0004**	0.000138***	2084
48 h	0.05mM Pb2+ vs. 0.25 mM Pb2+	1.02	0.188**	0.57***	4.27***	119***	0.6	-875***	-1.7	-9***	-3.84***	-0.355***	0	0.000256***	1719
48 h	0.05mM Pb2+ vs. 0.325 mM Pb2+	2.46**	0.308***	-0.05	1.67***	16	-9.7***	-1009***	-10.5***	-10***	-3.7***	-0.209***	-0.00054***	0.000383***	-30572***
48 h	0.0625mM Pb2+vs 0.1 mM Pb2+	0.87	0.081	-0.4***	-0.1	32**	18.9***	-1238***	3.7	-7.1***	-3.17***	-1.042***	-0.00004	0.000125***	-2879
48 h	0.0625mM vs. 0.25 mM Pb2+	1.08	0.143*	0.24***	1.4***	23*	16.6***	-649***	1.3	-6.1**	-3.53***	-0.266***	0.00035**	0.000242***	-3244
48 h	0.0625mM vs. 0.325 mM Pb2+	2.51**	0.262***	-0.38***	-1.2***	-80***	6.3***	-783***	-7.5*	-7.1***	-3.38***	-0.12*	-0.00018	0.000369***	-35535***
48 h	0.1 mM Pb2+ vs 0.25 mM Pb2+	0.2	0.062	0.64***	1.5***	-9	-2.3	589***	-2.4	1	-0.36	0.776***	0.00039**	0.000118***	-365
48 h	0.1 mM Pb2+ vs 0.325 mM Pb2+	1.64*	0.181**	0.02	-1.1***	-112***	-12.6***	455***	-11.2***	0	-0.21	0.922***	-0.00014	0.000244***	-32656***
48 h	0.25 mM Pb2+vs 0.325 mM Pb2+	1.44	0.119	-0.62***	-2.6***	-103***	-10.3***	-134***	-8.8**	-1	0.15	0.147*	-0.00053***	0.000127***	-32291***
48 h	control +aphids vs. 0.025mM Pb2+ +aphids	-0.43	-0.013	0.34***	-1.5***	-4	-26.2***	-222***	-57.4***	-0.2	-0.32	-0.031	0.00029*	-0.000013	-602
48 h	control +aphids vs. 0.05mM Pb2+ +aphids	-0.61	-0.011	0.47***	-6.9***	0	-21.3***	-46**	-89.6***	1.3	-0.06	0.081	0.00023	-0.000095**	-7391
48 h	control +aphids vs. 0.0625mM Pb2+ +aphids	-0.92	-0.008	-0.57***	-8.1***	-1	-3.6*	172***	-24.6***	-4.7*	-0.41	-0.105	0.00016	-0.00008*	-12611**
48 h	control +aphids vs. 0.1 mM Pb2+ +aphids	-0.19	0.047	-0.47***	-3.23***	-2	-2.4	-250***	-44.2***	-8.2***	-0.08	-0.026	0.00032*	0.000098**	-1332
48 h	control +aphids vs. 0.25 mM Pb2+ +aphids	-0.19	0.139*	-0.25***	-0.53	-5	-1.9	-610***	-90.1***	0.3	-1.77***	-0.215***	0.00039**	0.000065	-10592*
48 h	control +aphids vs. 0.325 mM Pb2+ +aphids	1.28	0.217***	-0.09*	-3.93***	-12	-34.7***	-145***	-155.6***	-2.7	-3.34***	-0.501***	0.0005***	-0.000114***	-32936***
48 h	0.025mM Pb2+ +aphids vs 0.05mM Pb2+ +aphids	-0.17	0.003	0.13***	-5.4***	4	4.9**	176***	-32.2***	1.4	0.26	0.112	-0.00006	-0.000082*	-6789
48 h	0.025mM Pb2+ +aphids vs 0.0625mM Pb2+ +aphids	-0.48	0.005	-0.91***	-6.6***	3	22.6***	394***	32.8***	-4.6*	-0.09	-0.074	-0.00012	-0.000067*	-12010**
48 h	0.025mM Pb2+ +aphids vs 0.1 mM Pb2+ +aphids	0.24	0.06	-0.81***	-1.73***	1	23.8***	-28	13.2***	-8.1***	0.23	0.005	0.00003	0.000111***	-730
48 h	0.025mM Pb2+ +aphids vs. 0.25 mM Pb2+ +aphids	0.25	0.152*	-0.59***	0.97***	-1	24.3***	-388***	-32.7***	0.4	-1.46***	-0.184**	0.00011	0.000077*	-9990*
48 h	0.025mM Pb2+ +aphids vs. 0.325 mM Pb2+ +aphids	1.72*	0.231***	-0.43***	-2.43***	-8	-8.5***	77***	-98.2***	-2.6	-3.02***	-0.469***	0.00021	-0.000101**	-32334***
48 h	0.05mM Pb2+ +aphids vs 0.0625mM Pb2+ +aphids	-0.31	0.003	-1.04***	-1.2***	-2	17.8***	218***	65***	-6**	-0.35	-0.185**	-0.00006	0.000015	-5221
48 h	0.05mM Pb2+ +aphids vs 0.1 mM Pb2+ +aphids	0.42	0.058	-0.94***	3.67***	-3	18.9***	-204***	45.4***	-9.5***	-0.02	-0.106	0.00009	0.000193***	6059

48 h	0.05mM Pb2+ +aphids vs. 0.25 mM Pb2+ +aphids	0.42	0.15*	-0.72***	6.37***	-5	19.4***	-564***	-0.5	-1	-1.71***	-0.296***	0.00017	0.000159***	-3201
48 h	0.05mM Pb2+ +aphids vs. 0.325 mM Pb2+ +aphids	1.89*	0.228***	-0.56***	2.97***	-12	-13.4***	-99***	-66***	-4*	-3.28***	-0.581***	0.00027*	-0.000019	-25545***
48 h	0.0625mM Pb2+ +aphids vs 0.1 mM Pb2+ +aphids	0.72	0.055	0.1*	4.87***	-1	1.1	-422***	-19.6***	-3.5	0.32	0.079	0.00015	0.000178***	11279*
48 h	0.0625mM +aphids vs. 0.25 mM Pb2+ +aphids	0.73	0.147*	0.32***	7.57***	-4	1.7	-782***	-65.5***	5*	-1.37***	-0.11	0.00023	0.000144***	2020
48 h	0.0625mM +aphids vs. 0.325 mM Pb2+ +aphids	2.2**	0.225***	0.48***	4.17***	-11	-31.1***	-317***	-131***	2	-2.93***	-0.396***	0.00033**	-0.000034	-20324***
48 h	0.1 mM Pb2+ +aphids vs 0.25 mM Pb2+ +aphids	0.01	0.092	0.22***	2.7***	-2	0.5	-360***	-45.9***	8.5***	-1.69***	-0.189**	0.00008	-0.000034	-9260*
48 h	0.1 mM Pb2+ +aphids vs 0.325 mM Pb2+ +aphids	1.48	0.17**	0.38***	-0.7*	-10	-32.3***	105***	-111.4***	5.5**	-3.25***	-0.475***	0.00018	-0.000212***	-31604***
48 h	0.25 mM Pb2+ +aphids vs 0.325 mM Pb2+ +aphids	1.47	0.078	0.16***	-3.4***	-7	-32.8***	465***	-65.5***	-3	-1.56***	-0.286***	0.0001	-0.000178***	-22344***
48 h	0.025mM Pb2+vs 0.05mM Pb2+ +aphids	0.18	0.037	0.53***	-3.93***	223***	-15.8***	-680***	-241.6***	-11***	0.48	0.261***	0.00005	-0.000055	5522
48 h	0.025mM Pb2+vs 0.0625mM Pb2++aphids	-0.12	0.039	-0.51***	-5.13***	221***	1.9	-462***	-176.6***	-17***	0.14	0.075	-0.00001	-0.00004	301
48 h	0.025mM Pb2+vs 0.1 mM Pb2++aphids	0.6	0.094	-0.41***	-0.27	220***	3.1	-884***	-196.3***	-20.5***	0.46	0.154*	0.00014	0.000138***	11580**
48 h	0.025mM Pb2+ vs. 0.25 mM Pb2++aphids	0.6	0.186**	-0.19***	2.43***	217***	3.6*	-1244***	-242.1***	-12***	-1.23***	-0.035	0.00022	0.000104**	2320
48 h	0.025mM Pb2+ vs. 0.325 mM Pb2++aphids	2.08**	0.265***	-0.03	-0.97***	210***	-29.2***	-779***	-307.7***	-15***	-2.79***	-0.32***	0.00032**	-0.000074*	-20024***
48 h	0.05mM Pb2+vs 0.0625mM Pb2+ +aphids	0.33	0.079	-0.4***	-3.43***	158***	-3.9*	-283***	-180.1***	-16***	-0.69*	-0.13*	0.00001	0.000088**	150
48 h	0.05mM Pb2+ vs 0.1 mM Pb2++aphids	1.05	0.134*	-0.3***	1.43***	157***	-2.8	-704***	-199.8***	-19.5***	-0.36	-0.051	0.00016	0.000266***	11430*
48 h	0.05mM Pb2+ vs. 0.25 mM Pb2++aphids	1.06	0.226***	-0.32	4.13***	154***	-2.2	-1065***	-245.6***	-11***	-2.05***	-0.24***	0.00024	0.000232***	2170
48 h	0.05mM Pb2+ vs. 0.325 mM Pb2++aphids	2.53**	0.304***	0.08*4	0.73*	147***	-35***	-599***	-311.2***	-14***	-3.62***	-0.526***	0.00034**	0.000053	-20174***
48 h	0.0625mM Pb2+vs 0.1 mM Pb2++aphids	1.11	0.089	-0.63***	-1.43***	60***	13.3***	-479***	-196.8***	-16.6***	-0.05	0.038	0.00052***	0.000252***	6467
48 h	0.0625mM vs. 0.25 mM Pb2++aphids	1.12	0.181**	-0.41***	1.27***	58***	13.8***	-839***	-242.6***	-8.1***	-1.74***	-0.152*	0.00059***	0.000218***	-2793
48 h	0.0625mM vs. 0.325 mM Pb2++aphids	2.59***	0.259***	-0.25***	-2.13***	51***	-19***	-374***	-308.2***	-11.1***	-3.3***	-0.437***	0.0007***	0.00004	-25137***
48 h	0.1 mM Pb2+ vs 0.25 mM Pb2+ +aphids	0.24	0.1	-0.01	1.37***	26*	-5.1**	399***	-246.3***	-1	1.43***	0.89***	0.00063***	0.000093**	86
48 h	0.1 mM Pb2+ vs 0.325 mM Pb2++aphids	1.72*	0.178**	0.15***	-2.03***	19	-37.9***	864***	-311.9***	-4*	-0.13	0.605***	0.00074***	-0.000085*	-22258***
48 h	0.25 mM Pb2+vs 0.325 mM Pb2++aphids	1.51	0.116	-0.49***	-3.53***	28*	-35.6***	276***	-309.5***	-5*	0.23	-0.171**	0.00034**	-0.000203***	-21893***
48 h	0.025mM Pb2++ aphids vs 0.05mM Pb2+ +aphids	-0.17	0.003	0.13***	-5.4***	4	4.9**	176***	-32.2***	1.4	0.26	0.112	-0.00006	-0.000082*	-6789
48 h	0.025mM Pb2+ + aphids vs 0.0625mM Pb2++aphids	-0.48	0.005	-0.91***	-6.6***	3	22.6***	394***	32.8***	-4.6*	-0.09	-0.074	-0.00012	-0.000067*	-12010**
48 h	0.025mM Pb2+ + aphids vs 0.1 mM Pb2++aphids	0.24	0.06	-0.81***	-1.73***	1	23.8***	-28	13.2***	-8.1***	0.23	0.005	0.00003	0.000111***	-730
48 h	0.025mM Pb2+ + aphids vs. 0.25 mM Pb2++aphids	0.25	0.152*	-0.59***	0.97***	-1	24.3***	-388***	-32.7***	0.4	-1.46***	-0.184**	0.00011	0.000077*	-9990*
48 h	0.025mM Pb2+ + aphids vs. 0.325 mM Pb2++aphids	1.72*	0.231***	-0.43***	-2.43***	-8	-8.5***	77***	-98.2***	-2.6	-3.02***	-0.469***	0.00021	-0.000101**	-32334***
48 h	0.05 mM Pb2+ + aphids vs 0.0625mM Pb2++ aphids	-0.31	0.003	-1.04***	-1.2***	-2	17.8***	218***	65***	-6**	-0.35	-0.185**	-0.00006	0.000015	-5221
48 h	0.05mM Pb2+ + aphids vs 0.1 mM Pb2+ + aphids	0.42	0.058	-0.94***	3.67***	-3	18.9***	-204***	45.4***	-9.5***	-0.02	-0.106	0.00009	0.000193***	6059
48 h	0.05mM Pb2+ + aphids vs. 0.25 mM Pb2++ aphids	0.42	0.15*	-0.72***	6.37***	-5	19.4***	-564***	-0.5	-1	-1.71***	-0.296***	0.00017	0.000159***	-3201
48 h	0.05mM Pb2++ aphids vs. 0.325 mM Pb2++ aphids	1.89*	0.228***	-0.56***	2.97***	-12	-13.4***	-99***	-66***	-4*	-3.28***	-0.581***	0.00027*	-0.000019	-25545***
48 h	0.0625mM Pb2++aphids vs 0.1 mM Pb2++aphids	0.72	0.055	0.1*	4.87***	-1	1.1	-422***	-19.6***	-3.5	0.32	0.079	0.00015	0.000178***	11279*
48 h	0.0625mM +aphids vs. 0.25 mM Pb2++aphids	0.73	0.147*	0.32***	7.57***	-4	1.7	-782***	-65.5***	5*	-1.37***	-0.11	0.00023	0.000144***	2020
48 h	0.0625mM +aphids vs. 0.325 mM Pb2++aphids	2.2**	0.225***	0.48***	4.17***	-11	-31.1***	-317***	-131***	2	-2.93***	-0.396***	0.00033**	-0.000034	-20324***
48 h	0.1 mM Pb2+ + aphids vs 0.25 mM Pb2++ aphids	0.01	0.092	0.22***	2.7***	-2	0.5	-360***	-45.9***	8.5***	-1.69***	-0.189**	0.00008	-0.000034	-9260*
48 h	0.1 mM Pb2+ + aphids vs 0.325 mM Pb2++ aphids	1.48	0.17**	0.38***	-0.7*	-10	-32.3***	105***	-111.4***	5.5**	-3.25***	-0.475***	0.00018	-0.000212***	-31604***
48 h	0.25 mM Pb2+ + aphids vs 0.325 mM Pb2++ aphids	1.47	0.078	0.16***	-3.4***	-7	-32.8***	465***	-65.5***	-3	-1.56***	-0.286***	0.0001	-0.000178***	-22344***
72 h	control vs. 0.025mM Pb2+	0.4	0.001	0.04	-0.8**	-271***	-0.3	-6	-0.8	-0.5	-1.29***	-0.157**	0.00002	-0.000099**	-8486
72 h	control vs. 0.05mM Pb2+	-0.06	-0.047	0.16***	-0.1	-100***	-7.7***	-118***	-14.4***	-3.5	-0.87**	-0.182**	-0.00006	-0.000146***	-10012*
72 h	control vs. 0.0625mM Pb2+	-0.27	-0.014	0.59***	-0.8**	-45***	-6.4***	-60***	-2.2	-6.7***	-0.26	-0.11	-0.00018	-0.0001**	-14395**
72 h	control vs. 0.1 mM Pb2+	0.08	0.02	-0.03	-0.4	50***	-3.3	-125***	0	-6.9***	-1.19***	0.208***	0.00018	-0.000075*	-33924***
72 h	control vs. 0.25 mM Pb2+	0.67	0.075	0.7***	6.4***	46***	-3.2	-281***	-1.8	-9.9***	-3.12***	-0.043	0.00007	-0.000061	-9668*
72 h	control vs. 0.325 mM Pb2+	1.69*	0.147*	0.94***	7***	22	-6.9***	-254***	-3.6	-9.6***	-3.54***	-0.138*	0.00023	-0.000016	-34547***

72 h	0.025mM Pb2+vs 0.05mM Pb2+	-0.45	-0.047	0.12**	0.7*	171***	-7.4***	-112***	-13.6***	-3	0.42	-0.026	-0.00008	-0.000048	-1525
72 h	0.025mM Pb2+vs 0.0625mM Pb2+	-0.67	-0.015	0.55***	0	227***	-6.1***	-53**	-1.4	-6.2**	1.04***	0.046	-0.00021	-0.000002	-5908
72 h	0.025mM Pb2+vs 0.1 mM Pb2+	-0.32	0.019	-0.07	0.4	321***	-3.1	-119***	0.8	-6.4***	0.11	0.364***	0.00015	0.000024	-25438***
72 h	0.025mM Pb2+ vs. 0.25 mM Pb2+	0.27	0.074	0.66***	7.2***	317***	-2.9	-275***	-1	-9.5***	-1.82***	0.113	0.00004	0.000037	-1182
72 h	0.025mM Pb2+ vs. 0.325 mM Pb2+	1.29	0.146*	0.9***	7.8***	293***	-6.7***	-248***	-2.8	-9.1***	-2.25***	0.019	0.00021	0.000082*	-26061***
72 h	0.05mM Pb2+vs 0.0625mM Pb2+	-0.21	0.032	0.43***	-0.7*	56***	1.3	58***	12.2***	-3.2	0.61*	0.072	-0.00013	0.000046	-4383
72 h	0.05mM Pb2+ vs 0.1 mM Pb2+	0.14	0.066	-0.19***	-0.3	151***	4.3*	-7	14.4***	-3.4	-0.31	0.39***	0.00024	0.000071*	-23912***
72 h	0.05mM Pb2+ vs. 0.25 mM Pb2+	0.73	0.121	0.54***	6.5***	146***	4.5*	-163***	12.6***	-6.5***	-2.25***	0.139*	0.00013	0.000085*	344
72 h	0.05mM Pb2+ vs. 0.325 mM Pb2+	1.75*	0.193**	0.78***	7.1***	122***	0.7	-136***	10.9***	-6.1**	-2.67***	0.044	0.00029*	0.00013***	-24535***
72 h	0.0625mM Pb2+vs 0.1 mM Pb2+	0.35	0.034	-0.62***	0.4	95***	3	-65***	2.2	-0.2	-0.93***	0.318***	0.00036**	0.000025	-19529***
72 h	0.0625mM vs. 0.25 mM Pb2+	0.94	0.089	0.11**	7.2***	91***	3.2	-222***	0.4	-3.3	-2.86***	0.067	0.00025*	0.000039	4727
72 h	0.0625mM vs. 0.325 mM Pb2+	1.96*	0.161*	0.35***	7.8***	66***	-0.6	-195***	-1.3	-2.9	-3.28***	-0.028	0.00042***	0.000084*	-20152***
72 h	0.1 mM Pb2+ vs 0.25 mM Pb2+	0.59	0.055	0.73***	6.8***	-4	0.2	-156***	-1.8	-3.1	-1.93***	-0.251***	-0.00011	0.000014	24256***
72 h	0.1 mM Pb2+ vs 0.325 mM Pb2+	1.61*	0.127	0.97***	7.4***	-28*	-3.6*	-129***	-3.5	-2.7	-2.36***	-0.346***	0.00006	0.000059	-623
72 h	0.25 mM Pb2+vs 0.325 mM Pb2+	1.02	0.072	0.24***	0.6*	-24*	-3.8*	27	-1.8	0.3	-0.42	-0.095	0.00017	0.000045	-24879***
72 h	control +aphids vs. 0.025mM Pb2+ +aphids	-0.02	-0.011	0.22***	-3.7***	4	-4.6*	21	13.8***	-9.3***	0.55*	0.09	-0.00006	0.000067*4	-8465
72 h	control +aphids vs. 0.05mM Pb2+ +aphids	-0.13	-0.024	0.52***	0.9**	1	-0.1	-29	17.2***	-8.3***	0.31	0.08	-0.00009	0.000007	7391
72 h	control +aphids vs. 0.0625mM Pb2+ +aphids	-0.28	-0.069	0.47***	0.77*	2	2.5	-79***	9.9***	-1.8	0.71*	0.137*	0.00025*	-0.000031	-2449
72 h	control +aphids vs. 0.1 mM Pb2+ +aphids	-0.05	0.024	-0.027	-0.63*	1	-0.2	6	0.3	-2.6	0	0.15*	-0.00017	-0.000045	-6682
72 h	control +aphids vs. 0.25 mM Pb2+ +aphids	0.8	0.053	0.173***	4.37***	3	2.7	29	-16.2***	-10***	-1.08***	0.082	-0.00037**	-0.000241***	-20410***
72 h	control +aphids vs. 0.325 mM Pb2+ +aphids	2.23**	0.203**	2.503***	4.57***	-20	-6.6***	42*	-45.3***	-3.5	-1.3***	-0.024	-0.00007	-0.000094**	-50338***
72 h	0.025mM Pb2+ +aphids vs 0.05mM Pb2+ +aphids	-0.11	-0.013	0.3***	4.6***	-3	4.5*	-50**	3.4	1	-0.24	-0.009	-0.00004	-0.00006	15856***
72 h	0.025mM Pb2+ +aphids vs 0.0625mM Pb2+ +aphids	-0.25	-0.058	0.25***	4.47***	-2	7.1***	-100***	-3.9	7.5***	0.16	0.047	0.00031*	-0.000099**	6016
72 h	0.025mM Pb2+ +aphids vs 0.1 mM Pb2+ +aphids	-0.03	0.035	-0.247***	3.07***	-2	4.4*	-15	-13.5***	6.7***	-0.56*	0.06	-0.00012	-0.000112***	1783
72 h	0.025mM Pb2+ +aphids vs. 0.25 mM Pb2+ +aphids	0.83	0.064	-0.047	8.07***	-1	7.3***	8	-30***	-0.7	-1.63***	-0.008	-0.00031*	-0.000309***	-11945**
72 h	0.025mM Pb2+ +aphids vs. 0.325 mM Pb2+ +aphids	2.25**	0.214***	2.283***	8.27***	-24*	-2	21	-59.1***	5.7**	-1.85***	-0.114	-0.00001	-0.000162***	-41873***
72 h	0.05mM Pb2+ +aphids vs 0.0625mM Pb2+ +aphids	-0.14	-0.045	-0.05	-0.13	1	2.6	-50**	-7.3*	6.5***	0.41	0.057	0.00034**	-0.000039	-9840*
72 h	0.05mM Pb2+ +aphids vs 0.1 mM Pb2+ +aphids	0.08	0.048	-0.547***	-1.53***	1	0	35*	-16.8***	5.7**	-0.31	0.07	-0.00008	-0.000052	-14072**
72 h	0.05mM Pb2+ +aphids vs. 0.25 mM Pb2+ +aphids	0.94	0.077	-0.347***	3.47***	2	2.8	58***	-33.3***	-1.7	-1.39***	0.001	-0.00028*	-0.000249***	-27801***
72 h	0.05mM Pb2+ +aphids vs. 0.325 mM Pb2+ +aphids	2.36**	0.226***	1.983***	3.67***	-21	-6.5***	71***	-62.4***	4.7*	-1.61***	-0.105	0.00002	-0.000102**	-57729***
72 h	0.0625mM Pb2+ +aphids vs 0.1 mM Pb2+ +aphids	0.23	0.094	-0.497***	-1.4***	-1	-2.6	85***	-9.6***	-0.8	-0.72*	0.013	-0.00042***	-0.000013	-4232
72 h	0.0625mM +aphids vs. 0.25 mM Pb2+ +aphids	1.08	0.122	-0.297***	3.6***	1	0.2	107***	-26.1***	-8.2***	-1.79***	-0.055	-0.00062***	-0.00021***	-17961***
72 h	0.0625mM +aphids vs. 0.325 mM Pb2+ +aphids	2.51**	0.272***	2.033***	3.8***	-22	-9.1***	121***	-55.2***	-1.8	-2.01***	-0.162**	-0.00032*	-0.000063	-47889***
72 h	0.1 mM Pb2+ +aphids vs 0.25 mM Pb2+ +aphids	0.85	0.029	0.2***	5***	1	2.9	23	-16.5***	-7.4***	-1.07***	-0.068	-0.0002	-0.000197***	-13729**
72 h	0.1 mM Pb2+ +aphids vs 0.325 mM Pb2+ +aphids	2.28**	0.178**	2.53***	5.2***	-22	-6.4***	36*	-45.6***	-1	-1.3***	-0.175**	0.00011	-0.00005	-43657***
72 h	0.25 mM Pb2+ +aphids vs 0.325 mM Pb2+ +aphids	1.43	0.149*	2.33***	0.2	-92	-9.3***	13	-29.1***	6.4***	-0.22	-0.106	0.0003*	0.000147***	-29928***
72 h	0.025mM Pb2+vs 0.05mM Pb2+ +aphids	-0.43	0.003	0.67***	2.5***	363***	-5**	-139***	-16.1***	-2.5	0.79**	0.191**	-0.00005	0.000182***	3910
72 h	0.025mM Pb2+vs 0.0625mM Pb2++aphids	-0.58	-0.043	0.62***	2.37***	365***	-2.4	-188***	-23.4***	4*	1.2***	0.248***	0.00029*	0.000143***	-5930
72 h	0.025mM Pb2+vs 0.1 mM Pb2++aphids	-0.35	0.051	0.123**	0.97***	364***	-5**	-103***	-33***	3.2	0.48	0.261***	-0.00013	0.00013***	-10162*
72 h	0.025mM Pb2+ vs. 0.25 mM Pb2++aphids	0.5	0.08	0.323***	5.97***	365***	-2.1	-81***	-49.4***	-4.2*	-0.6*	0.192**	-0.00033**	-0.000067*	-23891***
72 h	0.025mM Pb2+ vs. 0.325 mM Pb2++aphids	1.93*	0.229***	2.653***	6.17***	342***	-11.4***	-67***	-78.6***	2.2	-0.82**	0.086	-0.00003	0.00008*	-53819***
72 h	0.05mM Pb2+vs 0.0625mM Pb2+ +aphids	-0.12	0.004	0.5***	1.67***	194***	5**	-76***	-9.7***	7***	0.77**	0.273***	0.00037**	0.000191***	-4404
72 h	0.05mM Pb2+ vs 0.1 mM Pb2++aphids	0.11	0.098	0.003	0.27	193***	2.4	8	-19.3***	6.2**	0.06	0.286***	-0.00005	0.000178***	-8637
72 h	0.05mM Pb2+ vs. 0.25 mM Pb2++aphids	0.96	0.127	0.203***	5.27***	195***	5.3**	31	-35.8***	-1.2	-1.02***	0.218***	-0.00025*	-0.000019	-22365***

72 h	0.05mM Pb2+ vs. 0.325 mM Pb2++aphids	2.39**	0.276***	2.533***	5.47***	172***	-4*	44**	-64.9***	5.2**	-1.24***	0.112	0.00006	0.000128***	-52293***
72 h	0.0625mM Pb2+vs 0.1 mM Pb2++aphids	0.32	0.066	-0.427***	0.97***	138***	1.1	-50**	-31.5***	9.4***	-0.56*	0.215***	0.00007	0.000132***	-4254
72 h	0.0625mM vs. 0.25 mM Pb2++aphids	1.17	0.095	-0.227***	5.97***	139***	4*	-27	-48***	2	-1.63***	0.146*	-0.00012	-0.000065	-17983***
72 h	0.0625mM vs. 0.325 mM Pb2++aphids	2.6***	0.244***	2.103***	6.17***	116***	-5.3**	-14	-77.1***	8.4***	-1.85***	0.04	0.00018	0.000082*	-47910***
72 h	0.1 mM Pb2+ vs 0.25 mM Pb2+ +aphids	0.82	0.061	0.393***	5.57***	44***	0.9	38*	-50.2***	2.2	-0.71*	-0.172**	-0.00048***	-0.00009**	1547
72 h	0.1 mM Pb2+ vs 0.325 mM Pb2++aphids	2.25**	0.21**	2.723***	5.77***	21	-8.4***	51**	-79.3***	8.6***	-0.93***	-0.278***	-0.00018	0.000057	-28381***
72 h	0.25 mM Pb2+vs 0.325 mM Pb2++aphids	1.66*	0.155*	1.993***	-1.03***	25*	-8.5***	208***	-77.6***	11.7***	1.01***	-0.027	-0.00007	0.000043	-52637***
72 h	0.025mM Pb2++ aphids vs 0.05mM Pb2+ +aphids	-0.11	-0.013	0.3***	4.6***	-3	4.5*	-50**	3.4	1	-0.24	-0.009	-0.00004	-0.00006	15856***
72 h	0.025mM Pb2+ + aphids vs 0.0625mM Pb2++aphids	-0.25	-0.058	0.25***	4.47***	-2	7.1***	-100***	-3.9	7.5***	0.16	0.047	0.00031*	-0.000099**	6016
72 h	0.025mM Pb2+ + aphids vs 0.1 mM Pb2++aphids	-0.03	0.035	-0.247***	3.07***	-2	4.4*	-15	-13.5***	6.7***	-0.56*	0.06	-0.00012	-0.000112***	1783
72 h	0.025mM Pb2+ + aphids vs. 0.25 mM Pb2++aphids	0.83	0.064	-0.047	8.07***	-1	7.3***	8	-30***	-0.7	-1.63***	-0.008	-0.00031*	-0.000309***	-11945**
72 h	0.025mM Pb2+ + aphids vs. 0.325 mM Pb2++aphids	2.25**	0.214***	2.283***	8.27***	-24*	-2	21	-59.1***	5.7**	-1.85***	-0.114	-0.00001	-0.000162***	-41873***
72 h	0.05 mM Pb2+ + aphids vs 0.0625mM Pb2++ aphids	-0.14	-0.045	-0.05	-0.13	1	2.6	-50**	-7.3*	6.5***	0.41	0.057	0.00034**	-0.000039	-9840*
72 h	0.05mM Pb2+ + aphids vs 0.1 mM Pb2+ + aphids	0.08	0.048	-0.547***	-1.53***	1	0	35*	-16.8***	5.7**	-0.31	0.07	-0.00008	-0.000052	-14072**
72 h	0.05mM Pb2+ + aphids vs. 0.25 mM Pb2++ aphids	0.94	0.077	-0.347***	3.47***	2	2.8	58***	-33.3***	-1.7	-1.39***	0.001	-0.00028*	-0.000249***	-27801***
72 h	0.05mM Pb2++ aphids vs. 0.325 mM Pb2++ aphids	2.36**	0.226***	1.983***	3.67***	-21	-6.5***	71***	-62.4***	4.7*	-1.61***	-0.105	0.00002	-0.000102**	-57729***
72 h	0.0625mM Pb2++aphids vs 0.1 mM Pb2++aphids	0.23	0.094	-0.497***	-1.4***	-1	-2.6	85***	-9.6***	-0.8	-0.72*	0.013	-0.00042***	-0.000013	-4232
72 h	0.0625mM +aphids vs. 0.25 mM Pb2++aphids	1.08	0.122	-0.297***	3.6***	1	0.2	107***	-26.1***	-8.2***	-1.79***	-0.055	-0.00062***	-0.00021***	-17961***
72 h	0.0625mM +aphids vs. 0.325 mM Pb2++aphids	2.51**	0.272***	2.033***	3.8***	-22	-9.1***	121***	-55.2***	-1.8	-2.01***	-0.162**	-0.00032*	-0.000063	-47889***
72 h	0.1 mM Pb2+ + aphids vs 0.25 mM Pb2++ aphids	0.85	0.029	0.2***	5***	1	2.9	23	-16.5***	-7.4***	-1.07***	-0.068	-0.0002	-0.000197***	-13729**
72 h	0.1 mM Pb2+ + aphids vs 0.325 mM Pb2++ aphids	2.28**	0.178**	2.53***	5.2***	-22	-6.4***	36*	-45.6***	-1	-1.3***	-0.175**	0.00011	-0.00005	-43657***
72 h	0.25 mM Pb2+ + aphids vs 0.325 mM Pb2++ aphids	1.43	0.149*	2.33***	0.2	-92	-9.3***	13	-29.1***	6.4***	-0.22	-0.106	0.0003*	0.000147***	-29928***

Table S3. Mean squares from two-way analysis of variance of observed traits in roots.

Source of variation	Time	Variant	Time × Variant	Residual
The number of degrees of freedom	3	13	32	98
Length of epicotyl	21.07***	56.19***	1.32	1.427
Fresh weight	0.0183***	0.1036***	0.0013	0.00126
Semiquinone radical	1.996***	0.1414***	0.0889***	0.00179
Mn^{2+} ions	1902***	546***	113***	0.43
ABA	2.12***	53.1***	11.1***	0.227
IAA	4465***	349.79***	238.3***	3.459
JA	1367017***	93772***	76443***	60.65
SA	32246***	3305***	2371***	9.277
Pisatin	170***	272***	195***	0.418
Glucose	0.3857***	0.3807***	0.4756***	0.0165
Sucrose	0.5001***	0.3357***	0.3092***	0.00043
Acid invertase	0.000009***	0.00000047***	0.00000039***	0.000000005
Alkaline invertase	0.000114***	0.0000299***	0.000022***	0.000000029
Total soluble sugar	0.00000167***	0.00000042***	0.00000026***	0.00000002

*** P<0.001

Table S4. Mean squares from two-way analysis of variance of observed traits in leaves.

Source of variation	Time	Variant	Time × Variant	Residual
The number of degrees of freedom	3	13	32	98
Length of epicotyl	52.35***	4.178***	0.304	0.913
Fresh weight	0.259***	0.053***	0.0027	0.006
Semiquinone radical	19.8***	1.127***	0.518***	0.0023
Mn^{2+} ions	1.98***	27.06***	23.30***	0.131
ABA	6435***	28164***	11291***	190.4
IAA	248***	308***	134***	4.826
JA	5172738***	836475***	390415***	421
SA	116281***	41743***	7320***	12.86
Pisatin	2517***	334***	171***	5.512
Glucose	11.79***	12.75***	0.858***	0.1157
Sucrose	0.288***	0.248***	0.134***	0.0053
Acid invertase	0.0000017***	0.0000004***	0.00000026***	0.000000022
Alkaline invertase	0.000000066***	0.00000005***	0.00000004***	0.000000002
Total soluble sugar	1532000000***	1773000000***	291500000***	29500000

** *P*<0.01; *** *P*<0.001

Table S5. The correlation coefficients and *p*-values for the values of individual traits in roots and leaves.

Trait	Correlation coefficient	*p*-value
1. Length of epicotyl	0.688 ***	<0.001
2. Fresh weight	0.784 ***	<0.001
3. Semiquinone radical	0.628 ***	<0.001
4. Mn^{2+} ions	0.293 *	0.0409
5. ABA	0.337 *	0.0179
6. IAA	0.146	0.3184
7. JA	0.754 ***	<0.001
8. SA	0.175	0.2304
9. Pisatin	-0.032	0.8249
10. Glucose	0.398 **	0.0046
11. Sucrose	0.636 ***	<0.001
12. Acid invertase	-0.080	0.5827
13. Alkaline invertase	0.073	0.6172
14. Total soluble sugar	0.223	0.1239

* *P*<0.05; ** *P*<0.01; *** *P*<0.001

Table S6. Effect of Pb and *A. pisum* on concentrations of semiquinone radicals and the Mn^{2+} ions in organs of pea seedlings detected by EPR in the roots and leaves. The data were obtained in three independent experiments and statistically analysed using ANOVA (at α = 0.05).

Time	Variant	Semiquinone radicals						Mn^{2+}					
		Leaves			Roots			Leaves			Roots		
		Mean		SE	Mean		SE	Mean		SE	Mean		SE
0 h	Control	1.237	f*	0.0219	0.3533	a	0.024	7.367	e	0.2186	5.7	b	0.4041
	0.025 mM Pb $(NO_3)_2$	1.747	c	0.0219	0.2433	bc	0.024	10.067	c	0.2186	6.2	ab	0.4041
	0.050 mM Pb $(NO_3)_2$	1.447	d	0.0219	0.2533	b	0.024	11.167	b	0.2186	7.3	a	0.4041
	0.0625 mM Pb $(NO_3)_2$	1.317	e	0.0219	0.1733	c	0.024	13.567	a	0.2186	2.4	c	0.4041
	0.1 mM Pb $(NO_3)_2$	1.837	b	0.0219	0.1833	bc	0.024	9.167	d	0.2186	5.6	b	0.4041
	0.25 mM Pb $(NO_3)_2$	1.217	f	0.0219	0.3433	a	0.024	9.567	cd	0.2186	1.6	c	0.4041
	0.325 mM Pb $(NO_3)_2$	2.097	a	0.0219	0.4033	a	0.024	6.667	f	0.2186	1.2	c	0.4041
	$LSD_{0.05}$	0.066			0.073			0.663			1.23		

	Control	1.657	c	0.0219	0.3533	def	0.024	9.467	e	0.2186	11.1	b	0.4041
	0.025 mM Pb $(NO_3)_2$	2.117	a	0.0219	0.4933	ab	0.024	10.367	d	0.2186	18.5	a	0.4041
	0.050 mM Pb $(NO_3)_2$	1.653	c	0.024	0.3333	def	0.024	8.267	f	0.2186	5.1	d	0.4041
	0.0625 mM Pb $(NO_3)_2$	1.663	c	0.024	0.3233	f	0.024	8.767	f	0.2186	2.2	gh	0.4041
	0.1 mM Pb $(NO_3)_2$	1.473	e	0.024	0.2833	f	0.024	7.433	g	0.2028	3.9	ef	0.4041
	0.25 mM Pb $(NO_3)_2$	0.743	h	0.024	0.4033	cd	0.024	7.433	g	0.2028	1.8	h	0.4041
	0.325 mM Pb $(NO_3)_2$	0.983	g	0.024	0.5233	a	0.024	4.833	h	0.2028	1.8	h	0.4041
24 h	Control + aphids	1.743	b	0.024	0.45	bc	0.0265	9.933	de	0.2028	12.3	b	0.4041
	0.025 mM Pb $(NO_3)_2$ + aphids	1.643	c	0.024	0.32	f	0.0265	12.933	c	0.2028	6.8	c	0.4041
	0.050 mM Pb $(NO_3)_2$ + aphids	1.553	d	0.024	0.21	g	0.0265	8.633	f	0.2028	3	fg	0.4041
	0.0625 mM Pb $(NO_3)_2$ + aphids	1.423	e	0.024	0.29	f	0.0265	9.433	e	0.2028	4.2	de	0.4041
	0.1 mM Pb $(NO_3)_2$ + aphids	1.263	f	0.024	0.33	ef	0.0265	13.033	c	0.2028	4.2	de	0.4041
	0.25 mM Pb $(NO_3)_2$ + aphids	1.043	g	0.024	0.4	cde	0.0265	16.633	a	0.2028	1.5	h	0.4041
	0.325 mM Pb $(NO_3)_2$ + aphids	0.693	h	0.0291	0.44	bc	0.0265	15.533	b	0.2028	1.9	gh	0.4041
	$LSD_{0.05}$	0.07			0.073			0.601			1.17		
	Control	2.073	b	0.0291	0.6933	d	0.0203	9.533	e	0.2028	20.7	d	0.4041
	0.025 mM Pb $(NO_3)_2$	1.733	ef	0.0291	0.5233	gh	0.0203	9.733	e	0.2028	17.6	e	0.4041
	0.050 mM Pb $(NO_3)_2$	1.843	d	0.0291	0.8233	c	0.0203	11.433	c	0.2028	17	e	0.4041
	0.0625 mM Pb $(NO_3)_2$	1.513	g	0.0291	1.2833	a	0.0203	8.567	f	0.2028	25.7	b	0.4041
	0.1 mM Pb $(NO_3)_2$	1.913	cd	0.024	0.5533	fgh	0.0203	8.667	f	0.2028	14.6	f	0.4041
	0.25 mM Pb $(NO_3)_2$	1.273	hi	0.024	0.5933	ef	0.0203	7.167	g	0.2028	13.4	g	0.4041
	0.325 mM Pb $(NO_3)_2$	1.893	cd	0.024	0.5633	fg	0.0203	9.767	e	0.2028	1.6	h	0.4041
48 h	Control + aphids	1.673	f	0.024	0.98	b	0.0231	6.767	g	0.2028	33.1	a	0.4041
	0.025 mM Pb $(NO_3)_2$ + aphids	1.333	h	0.024	0.5067	gh	0.0291	8.267	f	0.2028	22.8	c	0.4041
	0.050 mM Pb $(NO_3)_2$ + aphids	1.203	i	0.024	0.4867	h	0.0291	13.667	b	0.2028	12.6	g	0.4041
	0.0625 mM Pb $(NO_3)_2$ + aphids	2.243	a	0.024	0.6833	d	0.026	14.867	a	0.2028	32.4	a	0.4041
	0.1 mM Pb $(NO_3)_2$ + aphids	2.143	b	0.024	0.6333	de	0.026	10	e	0.2082	13.2	g	0.1732
	0.25 mM Pb $(NO_3)_2$ + aphids	1.923	c	0.024	0.3233	i	0.026	7.3	g	0.2082	1.3	h	0.1732
	0.325 mM Pb $(NO_3)_2$ + aphids	1.763	e	0.024	0.3633	i	0.026	10.7	d	0.2082	0.8	h	0.1732
	$LSD_{0.05}$	0.074			0.068			0.59			1.06		
	Control	3.493	a	0.024	1.1533	a	0.026	11.6	c	0.2082	24.8	de	0.1732
	0.025 mM Pb $(NO_3)_2$	3.453	a	0.024	0.8633	de	0.026	12.4	b	0.2082	25	d	0.1732
	0.050 mM Pb $(NO_3)_2$	3.333	b	0.024	0.8233	ef	0.026	11.7	c	0.2082	19.1	f	0.4041
	0.0625 mM Pb $(NO_3)_2$	2.903	d	0.0406	1.0333	b	0.026	12.4	b	0.2082	29.3	b	0.4041
	0.1 mM Pb $(NO_3)_2$	3.523	a	0.0406	0.8433	ef	0.024	12	bc	0.2082	27.2	c	0.3464
	0.25 mM Pb $(NO_3)_2$	2.793	e	0.0406	0.4933	i	0.024	5.2	g	0.2082	1.1	g	0.3464
	0.325 mM Pb $(NO_3)_2$	2.553	f	0.0406	0.6433	g	0.024	4.6	g	0.2082	1.8	g	0.3464
72 h	Control + aphids	3.303	b	0.0406	0.9333	cd	0.024	10.8	d	0.2082	30.7	a	0.3464
	0.025 mM Pb $(NO_3)_2$ + aphids	3.083	c	0.0406	0.7933	ef	0.024	14.5	a	0.2082	24.6	de	0.3464
	0.050 mM Pb $(NO_3)_2$ + aphids	2.783	e	0.0406	0.7733	f	0.024	9.9	e	0.2082	23.8	e	0.3464
	0.0625 mM Pb $(NO_3)_2$ + aphids	2.833	de	0.0406	0.9833	bc	0.024	10.033	e	0.2186	27.1	c	0.3464
	0.1 mM Pb $(NO_3)_2$ + aphids	3.33	b	0.0265	0.9633	bc	0.024	11.433	c	0.2186	27.1	c	0.4041
	0.25 mM Pb $(NO_3)_2$ + aphids	3.13	c	0.0265	0.5233	h	0.024	6.433	f	0.2186	1.7	g	0.4041
	0.325 mM Pb $(NO_3)_2$ + aphids	0.8	g	0.0265	0.4267	i	0.0219	6.233	f	0.2186	1.3	g	0.4041

LSD$_{0.05}$	0.1009	0.071	0.612	1.014

* In the columns, for individual times, the means denoted by the same letters are not significantly different. SE – standard error.

Table S7. Effect of lead and *A. pisum* on content of phytohormones in the organs of pea seedlings' roots and leaves. The data were obtained in three independent experiments and statistically analysed using ANOVA (α = 0.05).

Time	Variant	ABA						SA						JA						[illegible]		
		Leaves			Roots			Leaves			Roots			Leaves			Roots			Leaves		
		Mean		SE	Mean		SE	Mean		SE	Mean		SE	Mean		SE	Mean		SE	Mean		S[illegible]
0 h	Control	45.75	c*	12.124	2.199	c	0.346	8.18	a	1.27	9.6	c	1.27	57.6	d	12.3	12.7	d	3.002	6.88	abc	0.5[illegible]
	0.025 mM Pb $(NO_3)_2$	20.7	c	6.928	2.435	c	0.231	5.81	ab	1.905	10.19	c	1.905	164.3	b	12.87	94	c	2.078	5.22	c	1.1[illegible]
	0.050 mM Pb $(NO_3)_2$	41.52	c	10.525	1.857	c	0.133	7.82	ab	1.443	11.47	c	1.443	258.8	a	14.15	81.6	c	5.889	6.02	abc	0.9[illegible]
	0.0625 mM Pb $(NO_3)_2$	40.69	c	8.216	2.341	c	0.098	3.27	b	1.501	8.29	c	1.501	239.8	a	13.45	88.1	c	6.062	9.26	abc	0.8[illegible]
	0.1 mM Pb $(NO_3)_2$	46.13	c	10.311	4.121	b	0.254	6.75	ab	1.328	11.48	c	1.328	153	bc	14.15	136.2	b	5.485	5.9	bc	0.9[illegible]
	0.25 mM Pb $(NO_3)_2$	101.63	b	8.216	3.656	b	0.075	5.38	ab	1.501	16.59	b	1.501	121.8	c	14.03	132.2	b	3.637	9.11	ab	0.6[illegible]
	0.325 mM Pb $(NO_3)_2$	185.63	a	10.756	6.785	a	0.092	6.63	ab	1.617	41.6	a	1.617	132.2	bc	7.1	198.2	a	3.118	9.19	a	1.8[illegible]
	LSD$_{0.05}$	29.51			0.606			4.6			4.62			38.8			13.46			3.246		
24 h	Control	67.22	bc	6.928	4.931	c	0.098	26.2	i	1.386	33.68	i	1.425	188.1	k	12.87	345	e	2.021	9.47	de	1.3[illegible]
	0.025 mM Pb $(NO_3)_2$	74.74	b	7.506	5.087	c	0.254	56.48	g	1.501	71.81	d	1.41	321.5	i	14.15	231.5	i	5.889	5.98	e	1.3[illegible]
	0.050 mM Pb $(NO_3)_2$	26.22	e	8.66	0.889	j	0.075	19.24	j	1.386	50.24	h	1.395	191.3	k	13.45	289.8	gh	6.062	9.66	de	0.6[illegible]
	0.0625 mM Pb $(NO_3)_2$	31.68	e	5.774	3.436	e	0.092	30.19	i	1.617	23.77	k	1.38	391.4	h	14.15	169.2	k	6.004	6.64	e	1.2[illegible]
	0.1 mM Pb $(NO_3)_2$	26.71	e	6.928	2.825	f	0.081	20.47	j	1.328	93.02	b	1.365	1381.8	d	14.03	377.1	d	6.293	20.84	b	1.3[illegible]
	0.25 mM Pb $(NO_3)_2$	34.15	e	6.351	4.36	d	0.098	20.64	j	1.674	88.88	c	1.35	1767.9	a	7.1	698.6	c	5.658	31.33	a	1.3[illegible]
	0.325 mM Pb $(NO_3)_2$	84.06	ab	7.506	9.782	b	0.254	36.61	h	2.425	55.58	g	1.336	1707.3	b	12.87	720	b	4.907	13.76	c	1.3[illegible]
	Control + aphids	6.19	f	1.155	0.945	ij	0.075	212.75	a	2.078	55.95	g	1.321	333.1	i	14.15	298.1	g	3.233	7.86	e	1.4[illegible]
	0.025 mM Pb $(NO_3)_2$ + aphids	95.33	a	4.619	2.237	h	0.092	126.62	e	1.617	47.26	h	1.306	148.2	l	13.45	199.6	j	4.33	8.12	e	1.5[illegible]
	0.050 mM Pb $(NO_3)_2$ + aphids	53.69	cd	8.66	1.321	i	0.098	183.22	c	4.157	122.55	a	1.291	470.5	g	14.15	220.3	i	2.598	14.18	c	1.2[illegible]
	0.0625 mM Pb $(NO_3)_2$ + aphids	71.71	bc	6.928	2.731	fg	0.254	133.7	d	1.386	29.37	j	1.276	266	j	14.03	280	h	2.714	6.52	e	1.7[illegible]
	0.1 mM Pb $(NO_3)_2$ + aphids	27.91	e	6.928	3.987	d	0.075	137.53	d	1.501	85.12	c	1.261	578.5	f	7.1	325.2	f	3.464	15.79	c	1.3[illegible]
	0.25 mM Pb $(NO_3)_2$ + aphids	25.61	e	6.351	2.394	gh	0.092	84.63	f	1.386	63.04	f	1.247	712.1	e	12.87	761.1	a	3.233	12.4	cd	1.2[illegible]
	0.325 mM Pb $(NO_3)_2$ + aphids	34.66	de	5.774	11.161	a	0.081	199.26	b	1.617	67.66	e	1.232	1505.9	c	14.15	772.1	a	3.349	29.47	a	1.4[illegible]
	LSD$_{0.05}$	19.35			0.408			5.587			3.851			37.57			13.05			3.93		
48 h	Control	147.36	b	12.702	3.579	d	0.098	16.63	i	1.328	63.45	f	1.217	193.6	l	13.45	131.3	l	2.021	9.64	fg	0.5[illegible]
	0.025 mM Pb $(NO_3)_2$	228.15	a	13.279	3.63	d	0.254	24.23	h	1.674	53.99	h	1.202	152.1	m	14.15	161.3	k	5.889	10.79	f	1.1[illegible]
	0.050 mM Pb $(NO_3)_2$	164.86	b	16.166	3.706	d	0.075	20.74	hi	2.425	61.69	fg	1.187	331.6	k	14.03	431.4	i	6.062	4.95	hi	0.9[illegible]
	0.0625 mM Pb $(NO_3)_2$	68.69	c	6.928	3.204	d	0.092	23.73	h	2.078	112.52	d	1.172	557.4	j	7.1	476.9	g	6.004	20.96	d	0.8[illegible]
	0.1 mM Pb $(NO_3)_2$	36.72	de	5.774	2.52	e	0.098	20.04	hi	1.617	116.88	c	1.157	1795.5	a	12.87	458.2	h	6.293	2.08	i	0.9[illegible]
	0.25 mM Pb $(NO_3)_2$	46.07	d	7.506	6.876	b	0.254	22.44	hi	4.157	191.14	a	1.143	1206.7	d	14.15	450.2	h	5.658	4.34	hi	0.6[illegible]
	0.325 mM Pb $(NO_3)_2$	148.82	b	8.66	3.819	d	0.075	31.26	g	1.386	141.38	b	1.128	1340.8	c	13.45	126.5	l	4.907	14.66	e	1.8[illegible]
	Control + aphids	5.86	f	1.155	1.29	f	0.092	176.26	f	1.501	53.73	h	1.113	786.2	h	14.15	281.5	j	3.233	5.28	hi	1.3[illegible]
	0.025 mM Pb $(NO_3)_2$ + aphids	9.69	f	0.577	1.182	f	0.081	233.67	c	1.386	71.4	e	1.098	1007.9	e	14.03	562.8	d	4.33	31.49	b	1.3[illegible]
	0.050 mM Pb $(NO_3)_2$ + aphids	5.57	f	1.155	1.499	f	0.185	265.88	b	1.617	50.33	i	1.083	832.3	g	7.1	780.1	a	2.598	26.61	c	0.6[illegible]
	0.0625 mM Pb $(NO_3)_2$ + aphids	7.18	f	1.155	1.03	f	0.554	200.88	e	1.328	32.34	k	1.068	614.1	i	12.87	661.6	b	2.714	8.85	fg	1.2[illegible]
	0.1 mM Pb $(NO_3)_2$ + aphids	8.34	f	1.155	1.07	f	0.45	220.5	d	1.674	59.98	g	1.054	1036.1	e	14.15	505.1	f	3.464	7.7	fgh	1.3[illegible]
	0.25 mM Pb $(NO_3)_2$ + aphids	10.83	f	1.155	5.93	c	0.185	266.38	b	2.425	73.15	e	1.039	1396.3	b	13.45	525.6	e	3.233	7.17	gh	1.3[illegible]
	0.325 mM Pb $(NO_3)_2$ + aphids	17.97	ef	3.464	12.532	a	0.554	331.9	a	2.078	41.6	j	1.617	931	f	14.15	635.7	c	3.349	39.97	a	1.3[illegible]
	LSD$_{0.05}$	22.31			0.8			5.9			3.391			37.69			13.05			3.41		

72 h	Control	100.66	d	10.121	5.361	c	0.185	6.14	f	1.386	16.93	fg	3.118	19.1	j	8.26	27.7	g	2.021	6.28	g	1.3[illegible]
	0.025 mM Pb $(NO_3)_2$	371.72	a	11.738	6.928	b	0.554	6.97	f	1.501	20.24	ef	1.501	25.5	j	12.47	66.9	f	5.889	6.55	g	1.3[illegible]
	0.050 mM Pb $(NO_3)_2$	200.8	b	17.448	5.545	c	0.45	20.58	e	1.386	26.02	de	1.386	137.2	ef	10.74	74.4	def	6.062	13.95	bc	0.6[illegible]
	0.0625 mM Pb $(NO_3)_2$	145.18	c	8.227	5.1	c	0.306	8.37	f	1.617	12.69	g	1.617	78.9	i	8.37	71.7	ef	6.004	12.66	bcde	1.2[illegible]
	0.1 mM Pb $(NO_3)_2$	50.29	fg	9.959	2.988	de	0.237	6.17	f	1.328	15.84	fg	1.328	144.2	de	8.26	39.9	g	6.293	9.61	defg	1.3[illegible]
	0.25 mM Pb $(NO_3)_2$	54.56	ef	8.868	4.882	c	0.364	7.93	f	1.674	29.68	d	1.674	300.5	a	8.49	159.5	a	5.658	9.45	defg	1.3[illegible]
	0.325 mM Pb $(NO_3)_2$	78.77	de	8.216	9.433	a	0.3	9.72	f	2.425	62.56	a	2.425	273.5	b	7.62	74.8	def	4.907	13.22	bcd	1.3[illegible]
	Control + aphids	8.96	h	3.167	1.228	f	0.335	40.23	c	2.078	12.9	fg	2.078	135	ef	8.2	65.2	f	3.233	11.39	cdef	1.4[illegible]
	0.025 mM Pb $(NO_3)_2$ + aphids	5.42	h	2.656	1.304	f	0.312	26.45	de	1.617	14.2	fg	1.617	113.9	fgh	6.52	126.3	b	4.33	15.99	ab	1.5[illegible]
	0.050 mM Pb $(NO_3)_2$ + aphids	8.44	h	1.328	1.477	f	0.549	23.07	e	4.157	26.35	de	4.157	164	d	6.35	83.6	cde	2.598	11.51	cdef	1.2[illegible]
	0.0625 mM Pb $(NO_3)_2$ + aphids	6.98	h	3.58	2.275	ef	0.185	30.33	d	1.328	37.47	c	1.328	213.5	c	9.01	85.3	cd	2.714	8.93	efg	1.7[illegible]
	0.1 mM Pb $(NO_3)_2$ + aphids	7.56	h	3.406	1.432	f	0.433	39.92	c	2.598	11.53	g	2.598	128.9	efg	9.01	64.8	f	3.464	11.55	cdef	1.3[illegible]
	0.25 mM Pb $(NO_3)_2$ + aphids	6.25	h	1.732	1.233	f	0.491	56.4	b	2.656	11.9	g	2.656	106.3	gh	7.22	95.7	c	3.233	8.69	fg	1.2[illegible]
	0.325 mM Pb $(NO_3)_2$ + aphids	29.22	gh	9.353	3.655	d	0.433	85.52	a	4.965	53.98	b	4.965	92.9	hi	5.89	72.2	ef	3.349	17.99	a	1.4[illegible]
	$LSD_{0.05}$	24.38			1.116			7.078			7.401			24.56			13.05			3.9		

* In the columns, for individual times, the means denoted by the same letters are not significantly different. SE – standard error.

Table S8. Effect of lead and *A. pisum* on concentration of pisatin in the organs of pea seedlings' roots and leaves. The data were obtained in three independent experiments and statistically analysed using ANOVA (α = 0.05).

Time	Variant	Pisatin					
		Leaves			Roots		
		Mean		SE	Mean		SE
0 h	Control	13.55	b*	0.116	0.985	c	0.231
	0.025 mM Pb $(NO_3)_2$	16.92	b	0.231	1.515	c	0.173
	0.050 mM Pb $(NO_3)_2$	23.97	a	3.002	1.592	c	0.346
	0.0625 mM Pb $(NO_3)_2$	17.66	b	3.175	1.043	c	0.231
	0.1 mM Pb $(NO_3)_2$	17.58	b	2.598	1.193	c	0.116
	0.25 mM Pb $(NO_3)_2$	15.23	b	0.751	5.683	b	0.808
	0.325 mM Pb $(NO_3)_2$	18.77	ab	0.231	19.259	a	0.693
	$LSD_{0.05}$	5.91			1.358		
24 h	Control	4	j	0.116	0.177	ef	0.01
	0.025 mM Pb $(NO_3)_2$	5	j	0.15	0.428	c	0.025
	0.050 mM Pb $(NO_3)_2$	16	i	0.219	0.249	de	0.008
	0.0625 mM Pb $(NO_3)_2$	16	i	0.139	0.76	b	0.009
	0.1 mM Pb $(NO_3)_2$	22	g	0.139	0.235	de	0.008
	0.25 mM Pb $(NO_3)_2$	25	f	0.156	0.112	fg	0.01
	0.325 mM Pb $(NO_3)_2$	28	e	0.167	4.351	a	0.025
	Control + aphids	22	g	0.185	0.5	c	0.008
	0.025 mM Pb $(NO_3)_2$ + aphids	35	c	0.127	0.243	de	0.092
	0.050 mM Pb $(NO_3)_2$ + aphids	30	d	0.577	0.062	g	0.01
	0.0625 mM Pb $(NO_3)_2$ + aphids	19	h	1.155	0.3	d	0.025
	0.1 mM Pb $(NO_3)_2$ + aphids	44	b	1.155	0.3	d	0.008
	0.25 mM Pb $(NO_3)_2$ + aphids	45	ab	0.173	0.2	e	0.009
	0.325 mM Pb $(NO_3)_2$ + aphids	46	a	0.173	0.7	b	0.008
	$LSD_{0.05}$	1.4			0.082		
48 h	Control	1.95	h	0.173	0.98	ef	0.01
	0.025 mM Pb $(NO_3)_2$	2	h	0.116	0.96	ef	0.025
	0.050 mM Pb $(NO_3)_2$	3	h	0.173	0.19	g	0.008
	0.0625 mM Pb $(NO_3)_2$	5.9	g	0.173	0.15	g	0.009
	0.1 mM Pb $(NO_3)_2$	13	ef	0.289	0.19	g	0.01
	0.25 mM Pb $(NO_3)_2$	12	f	0.058	0.68	f	0.025
	0.325 mM Pb $(NO_3)_2$	13	ef	0.173	1.721	c	0.008
	Control + aphids	14.25	d	0.346	1.19	de	0.009
	0.025 mM Pb $(NO_3)_2$ + aphids	14.44	d	0.289	1.26	cde	0.081
	0.050 mM Pb $(NO_3)_2$ + aphids	13	ef	0.381	1.26	cde	0.019
	0.0625 mM Pb $(NO_3)_2$ + aphids	19	b	0.058	1.58	cd	0.055
	0.1 mM Pb $(NO_3)_2$ + aphids	22.5	a	0.173	1.22	de	0.045
	0.25 mM Pb $(NO_3)_2$ + aphids	14	de	0.346	5.288	b	0.185
	0.325 mM Pb $(NO_3)_2$ + aphids	17	c	1.155	57.308	a	0.554
	$LSD_{0.05}$	1.11			0.461		
72 h	Control	26.86	defg	1.732	1.948	cd	0.116
	0.025 mM Pb $(NO_3)_2$	27.32	defg	3.002	2.384	bcd	0.577
	0.050 mM Pb $(NO_3)_2$	30.32	cde	3.175	2.492	bcd	0.751

0.0625 mM Pb $(NO_3)_2$	33.54	abc	3.118	1.106	d	0.231
0.1 mM Pb $(NO_3)_2$	33.72	abc	3.406	3.936	b	0.635
0.25 mM Pb $(NO_3)_2$	36.79	a	2.771	1.718	cd	0.693
0.325 mM Pb $(NO_3)_2$	36.46	ab	2.021	7.745	a	0.751
Control + aphids	21.57	g	0.346	1.341	d	0.52
0.025 mM Pb $(NO_3)_2$ + aphids	30.84	bcde	1.443	1.595	cd	0.52
0.050 mM Pb $(NO_3)_2$ + aphids	29.85	cdef	0.289	2.703	bcd	0.751
0.0625 mM Pb $(NO_3)_2$ + aphids	23.32	g	0.173	2.439	bcd	0.058
0.1 mM Pb $(NO_3)_2$ + aphids	24.13	fg	0.577	1.276	d	0.173
0.25 mM Pb $(NO_3)_2$ + aphids	31.53	abcd	0.346	1.682	cd	0.808
0.325 mM Pb $(NO_3)_2$ + aphids	25.1	efg	0.462	3.305	bc	0.924
$LSD_{0.05}$	5.9			1.74		

* In the columns, for individual times, the means denoted by the same letters are not significantly different. SE – standard error.

Table S9. Effect of lead and *A. pisum* on sucrose and glucose contents as well as total soluble sugar content in the roots and leaves of pea seedlings. The data were obtained in three independent experiments and statistically analysed using ANOVA (α = 0.05).

Time	Variant	Sucrose						Glucose							
		Leaves			Roots			Leaves			Roots			Leaves	
		Mean		SE	Mean		SE	Mean		SE	Mean		SE	Mean	
0 h	Control	0.375	b	0.0145	0.0205	d	0.0029	3.609	d	0.1494	0.543	d	0.0301	60287	d
	0.025 mM Pb $(NO_3)_2$	0.4635	b	0.0462	0.0033	d	0.0018	4.114	c	0.0251	1.06	b	0.0178	43206	e
	0.050 mM Pb $(NO_3)_2$	0.3747	b	0.0028	0.0201	d	0.0094	4.312	bc	0.0485	1.74	a	0.0935	61103	d
	0.0625 mM Pb $(NO_3)_2$	0.3625	b	0.0741	0.0042	d	0.0003	3.594	d	0.3176	0.786	c	0.0103	75240	bc
	0.1 mM Pb $(NO_3)_2$	0.4577	b	0.0146	0.1497	b	0.0201	4.68	b	0.0147	0.575	d	0.0507	70427	cd
	0.25 mM Pb $(NO_3)_2$	0.7838	a	0.0781	0.0807	c	0.0017	5.428	a	0.1137	1.146	b	0.0366	82953	b
	0.325 mM Pb $(NO_3)_2$	0.7534	a	0.0146	0.202	a	0.0199	5.537	a	0.0773	0.522	d	0.0372	106822	a
	$LSD_{0.05}$	0.1374			0.034			0.437			0.1421			11846	
24 h	Control	0.3361	ef	0.0292	0.004	c	0.002	3.472	fg	0.1445	0.547	f	0.0516	60287	d
	0.025 mM Pb $(NO_3)_2$	0.2679	ef	0.0026	0.0164	c	0.001	3.338	fg	0.2084	0.419	f	0.0149	56656	de
	0.050 mM Pb $(NO_3)_2$	0.4374	cd	0.0285	0.0241	c	0.0088	4.016	e	0.0549	1.702	a	0.0813	49931	ef
	0.0625 mM Pb $(NO_3)_2$	0.3167	ef	0.0362	0.003	c	0.0025	3.739	ef	0.1674	1.113	c	0.0157	44431	fg
	0.1 mM Pb $(NO_3)_2$	0.9566	a	0.0376	0.0759	b	0.0174	4.977	d	0.0525	1.209	b	0.1538	55603	de
	0.25 mM Pb $(NO_3)_2$	0.3294	ef	0.0425	0.0263	c	0.0017	5.514	c	0.3484	0.402	f	0.0244	71867	c
	0.325 mM Pb $(NO_3)_2$	0.9291	a	0.0054	0.1857	a	0.0259	6.156	b	0.1153	0.448	f	0.0072	82566	ab
	Control + aphids	0.2531	f	0.0092	0	c	0	3.682	efg	0.0136	0.861	de	0.0651	40048	g
	0.025 mM Pb $(NO_3)_2$ + aphids	0.2916	ef	0.0242	0.0018	c	0.0015	3.286	g	0.0103	1.023	cd	0.0310	42949	fg
	0.050 mM Pb $(NO_3)_2$ + aphids	0.3211	ef	0.0302	0.01	c	0.0033	3.549	fg	0.0137	0.544	f	0.0182	76529	bc
	0.0625 mM Pb $(NO_3)_2$ + aphids	0.3491	de	0.0338	0.0143	c	0.0021	4	e	0.0284	1.145	c	0.0340	57945	de
	0.1 mM Pb $(NO_3)_2$ + aphids	0.4629	c	0.022	0.0022	c	0.0018	3.678	efg	0.1058	0.791	e	0.0222	42648	fg
	0.25 mM Pb $(NO_3)_2$ + aphids	0.6347	b	0.0361	0.0834	b	0.0005	5.389	c	0.1468	0.903	de	0.1335	55345	de
	0.325 mM Pb $(NO_3)_2$ + aphids	0.9837	a	0.0557	0.1608	a	0.0211	6.575	a	0.1101	0.79	e	0.0011	85101	a
	$LSD_{0.05}$	0.091			0.03			0.41			0.19			8083	
48 h	Control	0.5322	cd	0.0414	0.0042	f	0.0014	4.166	c	0.0594	0.861	def	0.0783	42411	e
	0.025 mM Pb $(NO_3)_2$	0.4381	def	0.0754	0	f	0	3.569	d	0.0441	1.11	bcd	0.0422	55130	c
	0.050 mM Pb $(NO_3)_2$	0.2327	hi	0.0149	0	f	0	2.744	f	0.0925	0.437	g	0.0228	54980	c
	0.0625 mM Pb $(NO_3)_2$	0.3214	fgh	0.003	0.0118	f	0.0013	3.06	ef	0.0593	1.023	cd	0.0768	50017	cde
	0.1 mM Pb $(NO_3)_2$	1.3633	a	0.0387	2.2176	a	0.0507	6.227	a	0.0711	2.047	a	0.2636	52896	cd
	0.25 mM Pb $(NO_3)_2$	0.5877	c	0.0457	0.732	b	0.021	6.588	a	0.1308	1.343	b	0.0879	53261	c
	0.325 mM Pb $(NO_3)_2$	0.4412	def	0.0087	0.2753	c	0.0305	6.44	a	0.0724	0.884	de	0.0207	85552	a
	Control + aphids	0.258	ghi	0.0721	0.0006	f	0.0005	3.024	ef	0.1299	0.66	efg	0.0423	42218	e
	0.025 mM Pb $(NO_3)_2$ + aphids	0.2891	ghi	0.0464	0.2598	c	0.0225	3.34	de	0.0847	0.614	efg	0.0350	42820	e
	0.050 mM Pb $(NO_3)_2$ + aphids	0.1773	i	0.0763	0.0108	f	0.0026	3.084	def	0.4307	0.571	fg	0.0432	49609	cde
	0.0625 mM Pb $(NO_3)_2$ + aphids	0.3627	efg	0.021	0	f	0	3.43	de	0.0683	1.061	bcd	0.0204	54829	c
	0.1 mM Pb $(NO_3)_2$ + aphids	0.2837	ghi	0.0175	0.0179	f	0.0006	3.106	def	0.1431	1.117	bcd	0.1106	43550	de
	0.25 mM Pb $(NO_3)_2$ + aphids	0.4729	cde	0.0311	0.1442	d	0.0066	4.796	b	0.0242	1.2	bc	0.0266	52810	cd
	0.325 mM Pb $(NO_3)_2$ + aphids	0.7585	b	0.0041	0.0919	e	0.0068	6.36	a	0.3659	0.874	def	0.2072	75154	b
	$LSD_{0.05}$	0.125			0.05			0.5			0.303			9536.5	
72 h	Control	0.3014	bcde	0.0621	0	e	0	2.084	f	0.4827	0.267	h	0.0116	36568	g
	0.025 mM Pb $(NO_3)_2$	0.458	a	0.0754	0.0022	e	0.0018	3.379	cd	0.0206	1.017	bc	0.0143	45054	ef

0.050 mM Pb $(NO_3)_2$	0.4835	a	0.0316	0	e	0	2.956	de	0.3599	0.865	cd	0.0215	46579	ef
0.0625 mM Pb $(NO_3)_2$	0.4116	ab	0.0228	0	e	0	2.343	ef	0.0294	0.568	ef	0.0343	50962	cde
0.1 mM Pb $(NO_3)_2$	0.0937	f	0.0091	0	e	0	3.269	cd	0.1348	0.456	fg	0.0352	70492	b
0.25 mM Pb $(NO_3)_2$	0.3446	abcd	0.0652	0.0721	d	0.0015	5.203	a	0.1271	0.882	bc	0.0033	46236	ef
0.325 mM Pb $(NO_3)_2$	0.4393	ab	0.0018	0.1664	a	0.0074	5.627	a	0.0383	1.452	a	0.0140	71115	b
Control + aphids	0.3474	abcd	0.089	0.003	e	0.0024	2.898	de	0.6122	0.71	de	0.0418	48535	def
0.025 mM Pb $(NO_3)_2$ + aphids	0.2575	cde	0.0323	0.0022	e	0.0002	2.344	ef	0.2706	0.341	gh	0.0248	56999	c
0.050 mM Pb $(NO_3)_2$ + aphids	0.2669	cde	0.0368	0	e	0	2.589	def	0.1644	0.51	f	0.0110	41144	fg
0.0625 mM Pb $(NO_3)_2$ + aphids	0.2101	def	0.0339	0.0034	e	0.0002	2.183	ef	0.0383	0.584	ef	0.0039	50984	cde
0.1 mM Pb $(NO_3)_2$ + aphids	0.1971	ef	0.0211	0.0014	e	0.0012	2.9	de	0.2891	0.561	ef	0.1322	55216	cd
0.25 mM Pb $(NO_3)_2$ + aphids	0.2655	cde	0.0719	0.1284	c	0.005	3.975	bc	0.0512	1.041	b	0.0374	68945	b
0.325 mM Pb $(NO_3)_2$ + aphids	0.3716	abc	0.0199	0.1564	b	0.005	4.195	b	0.2975	1.334	a	0.1315	98873	a
$LSD_{0.05}$	0.14			0.0084			0.8			0.16			8282	

* In the columns, for individual times, the means denoted by the same letters are not significantly different. SE – standard error.

Table S10. Effect of lead and *A. pisum* on acid invertase and alkaline invertase activities in the roots and leaves of pea seedlings. The data were obtained in three independent experiments and statistically analysed using ANOVA ($\alpha = 0.05$).

**Time**	**Variant**	**Acid invertase**						**Alkaline/neutral invertase**				
		Leaves			Roots			Leaves			Roots	
		Mean		SE	Mean		SE	Mean		SE	Mean	
0 h	Control	0.00001	b*	0	0.00026	g	0.000020	0.0001107	d	0.0000088	0.000048	cd
	0.025 mM Pb $(NO_3)_2$	0.00007	b	0.000006	0.00133	c	0.000012	0.0000793	d	0.0000154	0.000031	d
	0.050 mM Pb $(NO_3)_2$	0.00014	b	0.000056	0.00180	b	0.000023	0.0001361	cd	0.0000189	0	d
	0.0625 mM Pb $(NO_3)_2$	0.00005	b	0.000013	0.00194	a	0.000053	0.0001892	bc	0.0000208	0.000167	c
	0.1 mM Pb $(NO_3)_2$	0.00092	b	0.000016	0.00108	e	0.000010	0.0003446	a	0.0000440	0.000044	cd
	0.25 mM Pb $(NO_3)_2$	0.00109	a	0.000205	0.00121	d	0.000011	0.0002263	b	0.0000084	0.000736	a
	0.325 mM Pb $(NO_3)_2$	0.00102	a	0.000150	0.00054	f	0.000040	0.0003505	a	0.0000124	0.000342	b
	LSD$_{0.05}$	0.00030			0.00009			0.000065			0.0001306	
24 h	Control	0.00063	d	0.000055	0.00062	hi	0.000022	0.0001233	f	0.0000191	0.00461	e
	0.025 mM Pb $(NO_3)_2$	0.00119	bc	0.000076	0.00123	c	0.000020	0.0002189	de	0.0000190	0.01105	b
	0.050 mM Pb $(NO_3)_2$	0.00118	bc	0.000183	0.00099	def	0.000023	0.0003188	bc	0.0000521	0.00745	c
	0.0625 mM Pb $(NO_3)_2$	0.00132	b	0.000013	0.00105	de	0.000072	0.0001893	ef	0.0000092	0.01719	a
	0.1 mM Pb $(NO_3)_2$	0.00127	b	0.000043	0.00098	ef	0.000015	0.0001989	e	0.0000105	0.00331	g
	0.25 mM Pb $(NO_3)_2$	0.00127	b	0.000138	0.00166	b	0.000008	0.0003749	b	0.0000143	0.000204	i
	0.325 mM Pb $(NO_3)_2$	0.00165	a	0.000049	0.00069	ghi	0.000018	0.0003593	b	0.0000096	0.000094	i
	Control + aphids	0.00030	e	0.000027	0.00059	i	0.000044	0.0000387	g	0.0000051	0.000214	i
	0.025 mM Pb $(NO_3)_2$ + aphids	0.00068	d	0.000013	0.00116	cd	0.000045	0.0000338	g	0.0000065	0.000243	i
	0.050 mM Pb $(NO_3)_2$ + aphids	0.00075	d	0.000095	0.00201	a	0.000013	0.0001268	f	0.0000075	0.00123	h
	0.0625 mM Pb $(NO_3)_2$ + aphids	0.00061	d	0.000035	0.00177	b	0.000010	0.0002564	cde	0.0000489	0.00493	de
	0.1 mM Pb $(NO_3)_2$ + aphids	0.00104	c	0.000018	0.00083	fg	0.000185	0.000474	a	0.0000117	0.00509	d
	0.25 mM Pb $(NO_3)_2$ + aphids	0.00101	c	0.000010	0.00079	gh	0.000079	0.0005171	a	0.0000338	0.0039	f
	0.325 mM Pb $(NO_3)_2$ + aphids	0.00134	b	0.000066	0.00037	j	0.000023	0.0002852	cd	0.0000022	0.0032	g
	LSD$_{0.05}$	0.00022			0.00018			0.000068			0.00038	
48 h	Control	0.00048	e	0.000011	0.00012	def	0.000020	0.0000953	i	0.0000000	0.00125	cd
	0.025 mM Pb $(NO_3)_2$	0.00082	cd	0.000174	0.00027	a	0.000056	0.0003193	def	0.0000065	0.00147	c
	0.050 mM Pb $(NO_3)_2$	0.00084	cd	0.000147	0.00024	ab	0.000054	0.000447	a	0.0000129	0.00105	de
	0.0625 mM Pb $(NO_3)_2$	0.00120	ab	0.000153	0.00020	bc	0.000018	0.0004334	ab	0.0000138	0.00703	a
	0.1 mM Pb $(NO_3)_2$	0.00124	ab	0.000091	0.00007	def	0.000011	0.0003086	def	0.0000226	0.000766	ef
	0.25 mM Pb $(NO_3)_2$	0.00085	cd	0.000122	0.00007	def	0.000011	0.0001911	h	0.0000504	0.00187	b
	0.325 mM Pb $(NO_3)_2$	0.00138	a	0.000045	0.00006	ef	0.000006	0.0000643	i	0.0000067	0.000322	gh
	Control + aphids	0.00100	bc	0.000004	0.00008	def	0.000021	0.0002798	fg	0.0000119	0.000532	fg
	0.025 mM Pb $(NO_3)_2$ + aphids	0.00071	cde	0.000137	0.00013	cde	0.000036	0.0002925	ef	0.0000458	0.000818	ef
	0.050 mM Pb $(NO_3)_2$ + aphids	0.00077	cde	0.000043	0.00014	cd	0.000023	0.0003747	bcd	0.0000445	0.0012	cd
	0.0625 mM Pb $(NO_3)_2$ + aphids	0.00084	cd	0.000031	0.00005	f	0.000013	0.0003593	cde	0.0000042	0.000545	fg
	0.1 mM Pb $(NO_3)_2$ + aphids	0.00068	de	0.000054	0.00029	a	0.000008	0.0001815	h	0.0000139	0.000048	h
	0.25 mM Pb $(NO_3)_2$ + aphids	0.00061	de	0.000189	0.00014	cde	0.000005	0.0002153	gh	0.0000086	0.00104	de
	0.325 mM Pb $(NO_3)_2$ + aphids	0.00050	e	0.000013	0.00022	ab	0.000004	0.0003937	abc	0.0000282	0.000165	h
	LSD$_{0.05}$	0.00031			0.00008			0.00007			0.0003499	
72 h	Control	0.00069	cd	0.000074	0.00006	h	0.000006	0.0002941	ef	0.0000097	0.000114	l
	0.025 mM Pb $(NO_3)_2$	0.00066	cd	0.000004	0.00023	e	0.000032	0.0003927	bc	0.0000365	0.000421	k
	0.050 mM Pb $(NO_3)_2$	0.00075	bc	0.000002	0.00022	ef	0.000040	0.0004403	ab	0.0000138	0.00419	c

0.0625 mM Pb $(NO_3)_2$	0.00087	ab	0.000063	0.00021	ef	0.000041	0.0003946	abc	0.0000542	0.0029	g
0.1 mM Pb $(NO_3)_2$	0.00051	def	0.000094	0.00097	a	0.000032	0.0003691	cd	0.0000066	0.00348	f
0.25 mM Pb $(NO_3)_2$	0.00062	cde	0.000064	0.00039	cd	0.000000	0.0003553	cde	0.0000209	0.0024	h
0.325 mM Pb $(NO_3)_2$	0.00045	ef	0.000023	0.00036	d	0.000005	0.0003103	def	0.0000073	0.00207	j
Control + aphids	0.00062	cde	0.000006	0.00004	h	0.000005	0.0002181	g	0.0000163	0.00223	i
0.025 mM Pb $(NO_3)_2$ + aphids	0.00068	cd	0.000067	0.00016	fg	0.000012	0.0001507	h	0.0000072	0.00483	b
0.050 mM Pb $(NO_3)_2$ + aphids	0.00072	bc	0.000041	0.00013	g	0.000018	0.0002108	gh	0.0000355	0.004	d
0.0625 mM Pb $(NO_3)_2$ + aphids	0.00037	f	0.000070	0.00019	efg	0.000012	0.0002493	fg	0.0000060	0.00509	a
0.1 mM Pb $(NO_3)_2$ + aphids	0.00080	bc	0.000054	0.00080	b	0.000008	0.0002627	fg	0.0000071	0.00374	e
0.25 mM Pb $(NO_3)_2$ + aphids	0.00099	a	0.000039	0.00044	c	0.000008	0.0004595	a	0.0000084	0.00238	h
0.325 mM Pb $(NO_3)_2$ + aphids	0.00069	c	0.000127	0.00021	ef	0.000033	0.0003124	def	0.0000117	0.0021	j
$LSD_{0.05}$	0.00018			0.00007			0.000065			0.000084	

* In the columns, for individual times, the means denoted by the same letters are not significantly different. SE – standard error.

Table S11. The effect of the *HMM* and *IFS* in the leaves and roots. The data were obtained in three independent experiments and statistically analyzed using ANOVA (*p*-values <0.001).

Time	Variant	Leave						Root					
		HMM			*IFS*			*HMM*			*IFS*		
		Mean		s.e.	Mean		s.e.	Mean		s.e.	Mean		s.e.
0 h	Control	0.059	b	0.004	0.034	b	0.002	0.473	b	0.026	0.110	b	0.006
	0.075 mM $Pb(NO_3)_2$	0.052	b	0.003	0.027	b	0.001	0.315	b	0.017	0.024	b	0.001
	0.5 mM $Pb(NO_3)_2$	1.213	a	0.072	1.168	a	0.037	5.275	a	0.286	3.518	a	0.180
	$LSD_{0.05}$	0.145			0.075			0.574			0.361		
24 h	Control	0.083	c	0.006	0.067	c	0.002	0.831	c	0.045	0.505	c	0.026
	+ aphids	0.253	c	0.016	0.180	c	0.008	0.732	c	0.040	0.295	cd	0.017
	0.075 mM $Pb(NO_3)_2$	0.209	c	0.017	0.173	c	0.010	0.193	c	0.012	0.014	d	0.001
	0.075 mM $Pb(NO3)_2$ + aphids	1.875	b	0.115	2.179	b	0.062	0.273	c	0.016	0.021	d	0.001
	0.5 mM mM $Pb(NO_3)_2$	0.207	c	0.015	0.140	c	0.005	3.842	b	0.209	2.730	b	0.140
	0.5 mM $Pb(NO3)_2$ + aphids	8.448	a	0.499	9.839	a	0.292	10.060	a	0.562	5.646	a	0.288
	$LSD_{0.05}$	0.645			0.376			0.758			0.405		
48 h	Control	0.098	c	0.008	0.164	c	0.009	2.148	c	0.118	1.706	b	0.089
	+ aphids	0.331	c	0.024	0.662	b	0.023	1.862	c	0.103	1.725	b	0.091
	0.075 mM $Pb(NO_3)_2$	0.191	c	0.014	0.498	b	0.027	0.358	d	0.022	0.156	d	0.009
	0.075 mM $Pb(NO3)_2$ + aphids	2.335	b	0.137	5.112	a	0.140	0.704	d	0.039	0.819	c	0.043
	0.5 mM mM $Pb(NO_3)_2$	0.152	c	0.010	0.135	c	0.009	6.217	b	0.353	6.590	a	0.351
	0.5 mM $Pb(NO3)_2$ + aphids	3.508	a	0.215	5.333	a	0.184	7.502	a	0.412	7.121	a	0.366
	$LSD_{0.05}$	0.323			0.295			0.712			0.660		
72 h	Control	0.093	c	0.006	0.217	e	0.018	1.010	c	0.078	1.588	c	0.120
	+ aphids	1.884	b	0.151	4.791	c	0.277	1.057	c	0.063	1.130	cd	0.060
	0.075 mM $Pb(NO_3)_2$	0.240	c	0.016	1.115	d	0.051	0.606	cd	0.039	0.755	cd	0.044
	0.075 mM $Pb(NO3)_2$ + aphids	2.154	b	0.149	11.040	b	0.301	0.331	d	0.018	0.472	d	0.025
	0.5 mM mM $Pb(NO_3)_2$	0.368	c	0.023	0.401	de	0.013	4.847	b	0.273	6.313	b	0.336
	0.5 mM $Pb(NO3)_2$ + aphids	11.830	a	0.691	18.940	a	0.561	8.530	a	0.465	12.050	a	0.614
	$LSD_{0.05}$	0.909			0.876			0.692			0.899		
